# Preemptive use of anti-inflammatories and analgesics in oral surgery: a review of systematic reviews

**DOI:** 10.3389/fphar.2023.1303382

**Published:** 2024-01-24

**Authors:** Régis Penha Pimenta, Cristiane Midori Takahashi, Silvio Barberato-Filho, Delaine Cristina Ferreira McClung, Fabio da Silva Moraes, Isabela Muniz de Souza, Cristiane de Cássia Bergamaschi

**Affiliations:** ^1^ Pharmaceutical Sciences Graduate Course, University of Sorocaba, Sorocaba, São Paulo, Brazil; ^2^ Dental School, University of Sorocaba, Sorocaba, São Paulo, Brazil

**Keywords:** analgesics, oral surgical procedures, preoperative period, postoperative pain, anti-inflammatory

## Abstract

**Objectives:** This review of systematic reviews evaluated the effectiveness and safety of the preemptive use of anti-inflammatory and analgesic drugs in the management of postoperative pain, edema, and trismus in oral surgery.

**Materials and methods:** The databases searched included the Cochrane Library, MEDLINE, EMBASE, Epistemonikos, Scopus, Web of Science, and Virtual Health Library, up to March 2023. Pairs of reviewers independently selected the studies, extracted the data, and rated their methodological quality using the AMSTAR-2 tool.

**Results:** All of the 19 studies reviewed had at least two critical methodological flaws. Third molar surgery was the most common procedure (*n* = 15) and the oral route the most frequent approach (*n* = 14). The use of betamethasone (10, 20, and 60 mg), dexamethasone (4 and 8 mg), methylprednisolone (16, 20, 40, 60, 80, and 125 mg), and prednisolone (10 and 20 mg) by different routes and likewise of celecoxib (200 mg), diclofenac (25, 30, 50, 75, and 100 mg), etoricoxib (120 mg), ibuprofen (400 and 600 mg), ketorolac (30 mg), meloxicam (7.5, 10, and 15 mg), nimesulide (100 mg), and rofecoxib (50 mg) administered by oral, intramuscular, and intravenous routes were found to reduce pain, edema, and trismus in patients undergoing third molar surgery. Data on adverse effects were poorly reported.

**Conclusion:** Further randomized clinical trials should be conducted to confirm these findings, given the wide variety of drugs, doses, and routes of administration used.

## Introduction

Postoperative responses such as pain, swelling, and trismus resulting from a physiological inflammatory process following surgical tissue injury have been widely investigated in dentistry. Intense prolonged deleterious effects can occur if adequate management of the post-surgical inflammatory process is not carried out (Cetira Filho et al., 2020). Psychological, anatomical, and neurophysiological aspects, as well as the type of dental surgery and surgical technique, number among the variables that can influence immediate and late postoperative responses (Ashley et al., 2016; Fonseca et al., 2017). A greater degree of tissue trauma leads to more intense inflammatory signs and symptoms. Excessive inflammation often causes exacerbated pain, swelling, and postoperative trismus. This ultimately impairs the quality of life of patients, directly impacting their daily routine (Varvara et al., 2017).

Opioid and non-opioid analgesics, non-steroidal anti-inflammatory drugs (NSAIDs), and steroidal anti-inflammatory drugs (corticosteroids) are analgesics used to control the physiological effects of dental surgical procedures, attenuating inflammatory reactions (Fonseca et al., 2017). Knowing the ideal timing of the action of these drugs can improve effectiveness in reducing postoperative responses (Thieme et al., 2020).

The literature has shown that analgesia in oral surgery can be effective if administered to the patient prior to the start of the procedure. This concept is referred to as preemptive analgesia and aims to control postoperative pain, swelling, and trismus (Penprase et al., 2015).

Preventive drugs are those administered before the onset of tissue damage and maintained for a given period after the surgical procedure has been completed. By contrast, preemptive drugs are administered before the start of surgery and not continued after the intervention. Both of these protocols involve drug administration prior to surgical incision in a bid to minimize intraoperative nociception and postoperative inflammatory responses (Doleman et al., 2018).

NSAIDs and corticosteroids are commonly used agents after dental surgery, administered alone or in combination (Au et al., 2015; Cetira Filho et al., 2020). Although long-term steroid therapy can lead to adverse effects, such as adrenal insufficiency, increased risk of infection, hyperglycemia, hypertension, osteoporosis, and development of diabetes mellitus, these effects are unlikely to occur at the low doses commonly used in dental surgical procedures (Larsen et al., 2018).

There is a dearth of systematic reviews investigating the effectiveness and safety of the preemptive use of anti-inflammatory and analgesic drugs after oral surgeries. A previous overview of systematic reviews provided a summary of the available evidence on the effectiveness and safety of using opioid and non-opioid analgesics in acute dental pain (Moore et al., 2018). However, the study failed to address the preemptive use of corticosteroids in dental surgery, and the search only included studies published up until 2017. The present review synthesized the available evidence from findings of published systematic reviews on the effectiveness and safety of the preemptive use of analgesic and anti-inflammatory drugs in the management of postoperative pain, edema, and trismus in oral surgery. More specifically, the review question was as follows: is the preemptive use of anti-inflammatory and analgesic drugs effective and safe for the management of postoperative pain, edema, and trismus in patients undergoing dental surgery aged 12 years or older?

## Methods

### Study design and protocol registration

This review of systematic reviews followed the protocol recommended by the Cochrane Handbook for Systematic Reviews of Interventions (Higgins et al., 2022) and was described according to the Preferred Reporting Items for Systematic Reviews and Meta-Analyses (PRISMA) (Page et al., 2021). The protocol was registered on PROSPERO, under number CRD42022352803.

### Eligibility criteria

Eligibility criteria were described using the population, intervention, comparison, outcome, and type of study (PICOT) framework.

#### Inclusion criteria

**Population:** Patients, aged 12 years or older, undergoing dental surgery (third molar surgery, dental implant, periodontal surgery, dental extraction, soft and hard tissue grafts, or biopsies).

**Intervention:** Preemptive use of anti-inflammatory drugs (non-steroidal anti-inflammatory drugs/NSAIDs and corticosteroids) and analgesic drugs (opioids and non-opioids) at any dose.

**Outcomes:** Effectiveness and safety.

**Comparator:** Placebo or other active control.

**Type of study:** Systematic review of randomized clinical trials (RCTs).

#### Exclusion criteria

**Population:** Patients undergoing dental surgery procedures performed under general anesthesia; patients diagnosed with malignant neoplasms, diabetes *mellitus*, hypertension, liver and kidney diseases; and immunosuppressed patients.

**Intervention:** Studies in which the time of drug use was uncertain or the drug was administered via the rectal route.

**Type of study:** Systematic review with all the RCTs included in another review. Abstracts presented at scientific events were also excluded.

### Outcomes evaluated

*Primary outcomes*: Relief of inflammatory events such as pain, swelling/edema, and trismus. The description of symptoms may be by self-report, validated questionnaires, or clinical diagnosis.

*Secondary outcomes*: Improved quality of life, rescue medication, and the presence of adverse drug events.

### Research sources for primary studies

#### Electronic searches

The databases searched included the Cochrane Library, MEDLINE (via PubMed), EMBASE, Epistemonikos, Scopus, Web of Science, and Virtual Health Library. The search applied no language restrictions or time limits on the studies included. The information sources were searched to identify all relevant studies published up to 23 March 2023.

#### Search of other resources

The reference lists of the reviews included were also checked by the reviewers to identify other relevant studies. When necessary, the corresponding authors of the studies were contacted for additional information.

### Search strategy

The search strategy was devised using search terms based on Medical Subject Headings (MeSH) descriptors and is described in Supplementary Material SA.

### Eligibility determination

Pairs of reviewers (RP and IM; CT and FM) independently assessed potentially relevant titles and abstracts and applied the eligibility criteria. The full text of systematic reviews was obtained. The same reviewers independently assessed the eligibility of each full text and settled any disagreements by consensus. Calibration exercises were performed for data extraction by using a standardized Excel form. A third reviewer (CB or SB-F) helped reach a final decision, when necessary.

### Risk of bias

The quality of systematic reviews was assessed independently by the same pairs of reviewers using the Assessing the Methodological Quality of Systematic Reviews-2 (AMSTAR-2) tool (Shea et al., 2017). This tool assesses the methodological aspects of systematic reviews using 16 items and classifies the overall confidence in the review results as follows: high (no or one non-critical weakness), moderate (more than one non-critical weakness), low (one critical flaw with or without non-critical weaknesses), and critically low (more than one critical flaw with or without non-critical weaknesses). Any disagreements were settled by consensus.

### Data extraction and review

A data extraction worksheet was previously developed to record the information collected. Calibration exercises were conducted to ensure consistency across reviewers.

Extraction was carried out in accordance with the instruction manual prepared. Pairs of reviewers (CB and IM, CT and FM, and RP and DM) independently extracted the data and recorded information about patients, interventions, and comparators (drug, dose and route of administration, or placebo), follow-up time, and outcomes. Data were collected from systematic reviews, and, when necessary, RCTs were read to collect information.

In cases of missing information, the corresponding author of the study was contacted. Disagreements were settled by consensus, and any unresolved issues were referred to a third reviewer (CB or SB-F).

### Data synthesis

The results of the systematic reviews included were summarized by narrative synthesis and grouped by the type of surgery, drug class, and route of administration. The results of meta-analyses were collected based on outcomes assessed, according to the measures presented in the reviews [odds ratio, relative risk, weighted mean difference, 95% confidence interval (95% CI), and others].

The measure of heterogeneity was described mainly by I-square (I^2^), where 0%–25% indicated low heterogeneity, 50% moderate heterogeneity, and 75% high heterogeneity (Higgins et al., 2003).

The quality of evidence, when available, was collected for each outcome according to Grading of Recommendations Assessment, Development, and Evaluation (GRADE). This tool grades evidence as follows: *high quality* (confidence true effect is close to the estimate of effect); *moderate quality* (moderate confidence in effect estimate: true effect is likely to be close to the estimate of effect, but there is possibility it is substantially different); *low quality* (confidence in effect estimate is limited: true effect may be substantially different from the estimate of effect); and *very low quality* (very little confidence in effect estimate: true effect is likely to be substantially different from the estimate of effect) (Guyatt et al., 2013).

### Ethical aspects

Ethical approval was not required for the present review of systematic reviews as this type of study does not involve the presentation of individual patient data.

### Study selection

**FIGURE 1:**
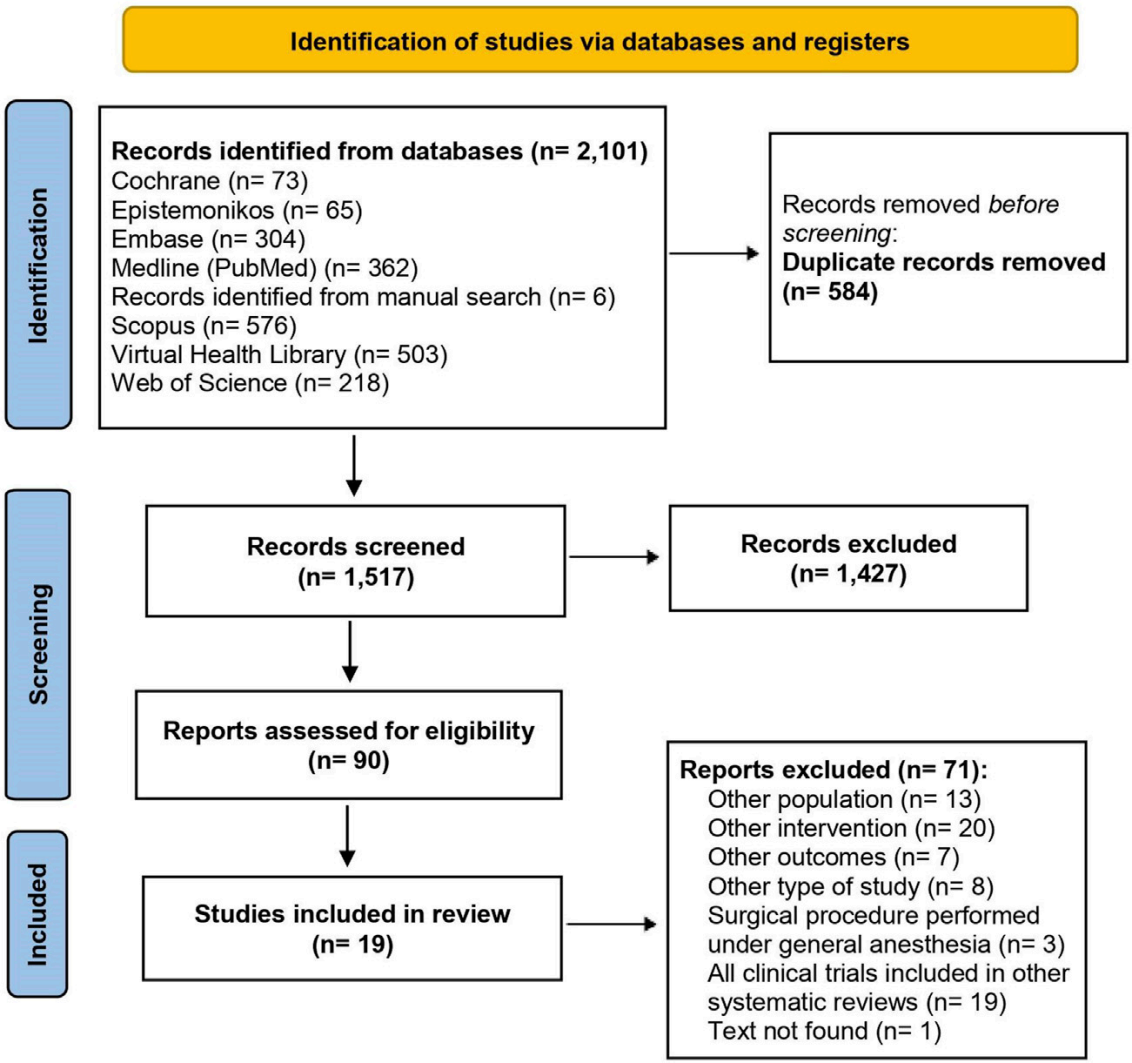
Flowchart of the included studies.

A total of 2,101 records were initially identified. After the removal of duplicate records, 1,517 studies remained for title and abstract screening. Of the 90 studies eligible for full text reading, 19 systematic reviews were selected for data extraction (Figure 1). The list of excluded studies and reasons for exclusion are given in Supplementary Material SB.

### Characteristics of studies included

Nineteen systematic reviews, encompassing a total of 203 clinical trials (with overlap), published between 2008 and 2022, were included. Regarding procedures performed, third molar surgery (*n* = 16) predominated, followed by periodontal (*n* = 2), and implant (*n* = 1) surgery. In general, the reviews included RCTs with different routes of drug administration, with the oral route being the most investigated (*n* = 12). Of the reviews about third molar surgery, eight compared the use of corticosteroids versus placebo, four compared corticosteroids versus other drugs or placebo, and six evaluated the use of NSAIDs versus NSAIDs or placebo Table 1.

**TABLE 1:** Characteristics of systematic reviews on preemptive use of anti-inflammatory drugs and analgesics in dental procedures (*n* = 19).

References (search period)	Number of preemptive RCT	Age of population (years)	Intervention vs. comparator (route of administration)	Main objective of review	Conclusions of review based on findings of preemptive use of drugs
Third Molar Surgery (Corticosteroids VS. Corticosteroids, Placebo, Or Other Drugs)					
^a^ Falci et al., 2017 (up to Apr/2015)	7	15–41 (Min-Max)	Corticosteroids **vs.** corticosteroids or NSAIDS or placebo (*Oral route*)	To assess the effectiveness of preemptive oral use of dexamethasone in lower third-molar extractions in terms of reducing pain, swelling and/or trismus compared with other oral anti-inflammatories	There is insufficient evidence through meta-analysis to conclude that oral dexamethasone is better than methylprednisolone or NSAIDS as a preemptive analgesic. Dexamethasone may be more effective than methylprednisolone for reducing swelling and trismus. The study collected no data on adverse effects
Almadhoon et al., 2022 (up to Sep/2021)	34	18–69 (Min-Max)	Corticosteroids **vs.** corticosteroids or placebo (*Oral, submucosal, intramuscular, and intravenous routes*)	To assess the comparative effects of different dexamethasone routes and doses on reducing postoperative sequelae after impacted mandibular third molars surgery	Dexamethasone, in different doses and routes, was superior to placebo in reducing pain, edema, and trismus, 1 day after surgical extraction. Submucosal dexamethasone 4 mg reduced pain until 3 days after extraction. No noteworthy difference was found between route and dose of dexamethasone. The study collected no data on adverse effects
^a^ Canellas et al., 2022 (up to 2021)	42	16–50 (Min-Max)	Corticosteroids **vs.** corticosteroids or placebo (*Oral, submucosal, intramuscular, intravenous, pterygomandibular routes*)	To compare the effects of different corticosteroids to minimize postoperative inflammatory complications after surgical extraction of the third molar by applying a network meta-analysis approach	Corticosteroids reduced inflammatory complications after third molar surgery. Dexamethasone 8 mg could be the best preoperative option to control inflammatory complications; however, more RCTs should be conducted to increase the quality of direct evidence. Few RCTs reported data on safety, but no serious adverse effects were observed. Surgeons should consider the use of dexamethasone submucosal, pterygomandibular, or oral routes to control these inflammatory complications
Parhizkar et al., 2022 (up to May/2021)	2	>15	Corticosteroids **vs.** placebo (*Submucosal and intravenous routes*)	To evaluate the efficacy of adjunctive corticosteroid therapy in improving patient-centered outcomes following third molar surgery	Dexamethasone 8 mg and methylprednisolone 40 mg (both via intravenous route) improved quality of life compared to placebo. Dexamethasone 4 mg (submucosal route) improved pain and swelling compared to placebo. No adverse effects have been reported
^a^ Almeida et al., 2019 (up to Apr/2017)	9	15–45 (Min-Max)	Corticosteroids **vs.** placebo (*Oral, submucosal, intramuscular, and intravenous routes*)	To examine the effectiveness of corticosteroids in the control of pain, edema, and trismus following third molar surgery	Corticosteroids had a positive effect with regard to the control of the pain, edema, and trismus in the surgical removal of impacted third molars. The study collected no data on adverse effects. Route of administration appeared not to influence the results, making the oral route an easy excellent option
Varvara et al., 2017 (up to Apr/2017)	6	Not reported	Corticosteroids **vs.** corticosteroids or placebo (*Oral, submucosal and intramuscular routes*)	To examine the different corticosteroids used in oral surgery procedures to define the time and route of administration	The use of corticosteroids in oral surgery is promising for the reduction of postoperative morbidity, edema, and trismus. The drugs’ effects on pain reduction remain a topic for further investigation. The study collected no data on adverse effects
Nagori et al., 2019 (up to Jan/2018)	11	22 ± 4.2 (Mean ± standard deviation)	Corticosteroids **vs.** placebo (*Oral, submucosal, and intravenous routes*)	To investigate the available evidence on whether methylprednisolone improves postoperative outcomes following impacted third molar surgery	Methylprednisolone improved pain and edema in the early postoperative period but had no effect on edema in late postoperative. The study collected no data on adverse effects. More high-quality RCTs are required to provide stronger evidence for the use of this corticosteroid
Herrera-Briones et al., 2013 (up to Sep/2011)	11	Not reported	Corticosteroids **vs.** corticosteroids or placebo (*Oral and intravenous routes*)	To conduct a systematic review on the preemptive use of corticosteroids in third molar surgery	The use of dexamethasone, methylprednisolone, and prednisolone improved the postoperative experience of patients, with a significant impact on trismus and inflammation. The study collected no data on adverse effects. Greater effects appear to be achieved by using the parenteral route
Markiewicz et al., 2008 (up to Mar/2007)	5	23.2 (Mean)	Corticosteroids **vs.** placebo (*Intramuscular and intravenous routes*)	To evaluate the use of perioperative corticosteroids compared to placebo for reducing postoperative edema, trismus, and pain in patients undergoing removal of the third molar	Betamethasone, dexamethasone, and methylprednisolone promoted mild-to-moderate reduction in edema and improvement in range of motion after undergoing removal of third molar compared to placebo. The study collected no data on adverse effects. These findings need to be confirmed
Third Molar Surgery (Nsaids VS Placebo Or Other Drugs)					
^a^ Cetera Filho et al., 2020 (up to Mar/2019)	20	22.0 ± 2.9 (Mean ± standard deviation)	NSAIDS or acetaminophen or corticosteroids **vs.** NSAIDS or opioids or placebo (*Oral route*)	To investigate the effectiveness of preemptive analgesia with NSAIDS for the relief of inflammatory events after surgical removal of third molars	Some NSAIDS were effective in controlling pain, edema, and trismus. Preemptive analgesia in the removal of third molars lowered average pain scores, especially in the first 6 h after surgery, and reduced average consumption of medication and the number of patients who needed it postoperatively. In general, the authors of the studies selected reported the main adverse effects as drowsiness, dizziness, headache, nausea, vomiting, trembling, sleepiness, allergy, syncope, and dyspnea
Magesty et al., 2021 (up to Aug/2020)	31	15–45 (Min-Max)	NSAIDS or corticosteroids **vs**. NSAIDS or placebo (*Oral route*)	To compare the effectiveness of oral pre-emptive analgesia administered for mandibular third molar surgery through a network meta-analysis	Reduction in average consumption of analgesics was observed with the use of nimesulide 100mg, dexamethasone 4 mg and 8mg, etoricoxib 120 mg and ibuprofen 600 mg compared to placebo. Reduction in pain was observed with the use of nimesulide 100 mg and reduction in edema with the use of dexamethasone 8 mg and diclofenac 50 mg and reduction in trismus with the use of ampiroxicam 27 mg and diclofenac 25 mg. The study collected no data on adverse effects
Isiordia-Espinosa et al., 2022 (up to Apr/2021)	3	Not reported	NSAIDS vs. NSAIDS vs. placebo (*Oral route*)	To evaluate the analgesic efficacy and adverse effects of celecoxib compared to non-opioid drugs after third molar surgery	Celecoxib 200 mg showed better analgesic efficacy than ibuprofen 400mg, acetaminophen 500 mg and placebo after 4, 8, and 24 postoperative hours following the third molar removal. The number of patients who required rescue analgesia was lower for celecoxib compared to non-opioid drugs. Lower number of gastrointestinal adverse effects with celecoxib compared with non-opioid treatments was observed
Khosraviani et al., 2020 (up to Jun/2018)	6	20–26 (Min-Max)	NSAIDS **vs**. NSAIDS or opioids or placebo (*Oral or intramuscular routes*)	To evaluate the effectiveness of meloxicam on post-operative pain management in patients who have undergone orofacial surgeries	Meloxicam had similar analgesic effects to naproxen 550mg, diflunisal 500mg, acetaminophen 500mg, rofecoxib 12.5 mg and nimesulide 100 mg and superior effects to ampiroxicam 27mg, diclofenac 100 mg and tramadol. One study reported mild nausea and vomiting and allergy as meloxicam-related complications
Silva et al., 2021 (up to Mar/2021)	1	18–35 (Min-Max)	NSAIDS **vs**. placebo (*Intravenous route*)	To assess the effect of preemptive intravenous ibuprofen on pain reduction after lower third molar surgery	Intravenous ibuprofen 800 mg, alone or combined with dexketoprofen, had the same perioperative analgesic efficacy and both were superior to placebo. No adverse effects were reported in the study
Romsing et al., 2004 (up to Jun/2003)	1	Not reported	NSAIDS **vs**. placebo (*Intravenous route*)	To review the analgesic efficacy of COX-2 inhibitor for post-operative pain relief after surgical removal of third molars	Intravenous administration of parecoxib 20mg, 40mg, 80 mg before oral surgery was effective and safe in providing preventive management of postoperative pain compared to placebo. No adverse effects were reported in the study
Tirupathi et al., 2021 (1980 to Jul/2020)	6	16–35 (Min-Max)	NSAIDS **vs**. corticosteroids or NSAIDS or tramadol or placebo (*Intramuscular and intravenous routes*)	To evaluate preemptive injected ketorolac in comparison to other agents for surgical removal of third molars	The studies showed better outcomes efficacy, but definitive conclusions cannot be made regarding the use of injected ketorolac for control of pain and edema. Thus, more clinical trials are needed to make definitive conclusions. In one RCT, four patients had complaints of nausea after receiving tramadol
Periodontal Surgery					
^a^ Caporossi et al., 2020 ^a^ (up to Sep/2019)	5	18–56 (Min-Max)	Corticosteroids and NSAIDS **vs.** corticosteroids or NSAIDS or placebo (*Oral, submucosal, intramuscular, and intravenous routes*)	To systematically review the literature on the pharmacological effect of different drugs on pain relief after periodontal surgeries	Dexamethasone (4mg and 8 mg), etoricoxib (120 mg), celecoxib (200 mg), or ketorolac (10mg and 20 mg) compared to placebo reduced postoperative pain, up to 8 h after the procedure. There is not enough evidence to suggest a standard treatment. The side effects observed were generally mild and equally distributed among treatment groups
Nir et al., 2016 ^b^ (up to Jan/2015)	1	17–85 (Min-Max)	NSAIDS **vs.** placebo (*Oral route*)	To evaluate the effectiveness of preoperative oral use of ketorolac for reducing analgesic consumption and minimizing postoperative pain	Ketorolac 20 mg reduced pain compared to placebo. No difference was observed between the groups regarding the use of rescue medication. No adverse effects related to preoperative ketorolac use were observed
Implant Surgery					
Melini et al., 2021 (up to May/2020)	2	17–85 (Min-Max)	NSAIDS **vs.** placebo (*Oral route*)	To summarize the available evidence on analgesics in the management of postoperative pain after dental implant placement	Dexketoprofen 25 mg and ibuprofen 600 mg were superior to placebo for reducing pain. One RCT reported bleeding due to the use of dexketoprofen. Further RCTs are needed to inform best practices in this domain

NSAIDS, Non-Steroidal Anti-Inflammatory Drugs. RCT, Randomized Clinical Trial.

^a^ Systematic reviews with specific meta-analysis data on preemptive drug use.

^b^ Non-specific reviews of dental studies.

^c^ Information available only in the review method.

### Risk of bias in studies

Most reviews had at least two critical flaws, mainly related to the fact that they failed to explain the selection criteria for the designs of the studies included (*n* = 15), failed to provide the list of excluded studies (*n* = 13), and failed to report on funding sources for clinical trials (*n* = 17) Table 2.

**TABLE 2:** Risk of bias of systematic reviews included (*n* = 19).

Systematic review	1	*2	3	*4	5	6	*7	8	*9	10	*11	12	*13	14	*15	16
Almadhoon et al. (2022)																
Almeida et al. (2019)																
Canellas et al. (2022)																
Caporossi et al. (2020)																
Cetira Filho et al. (2020)																
Falci et al. (2017)																
Herrera-Briones et al. (2013)																
Isiordia-Espinosa et al. (2022)																
Khosraviani et al. (2020)																
Magesty (2021)																
Markiewicz et al. (2008)																
Melini et al. (2021)																
Nagori et al. (2019)																
Nir et al. (2016)																
Romsing and Moiniche (2004)																
Parhizkar et al. (2022)																
Silva et al. (2021)																
Tirupathi et al. (2021)																
Varvara et al. (2017)																
Yes	Partial Yes	No	Not Applied													

*Issues considered by AMSTAR 2 to be critically important. 1. Did the research questions and inclusion criteria for the review include the components of PICO?

*2. Did the report of the review contain an explicit statement that the review methods were established prior to the conduct it, and did the report justify any significant deviations from the protocol?

3. Did the review authors explain their selection of the study designs for inclusion in review?

*4. Did the review authors use a comprehensive literature search strategy?

5. Did the review authors perform study selection in duplicate?

6. Did the review authors perform data extraction in duplicate?

*7. Did the review authors provide a list of excluded studies and justify the exclusions?

8. Did the review authors describe the included studies in adequate detail?

*9. Did the review authors use a satisfactory technique for assessing the risk of bias (RoB) in individual studies that were included in the review?

10. Did the review authors report on the sources of funding for the studies included in the review?

*11. If meta-analysis was performed, did the review authors use appropriate methods for statistical combination of results?

12. If meta-analysis was performed, did the review authors assess the potential impact of the RoB in individual studies on the results of the meta-analysis or other evidence synthesis?

13. Did the review authors account for the RoB in individual studies when interpreting/discussing the results of the review?

14. Did the review authors provide a satisfactory explanation for, and discussion of, any heterogeneity observed in the results of the review?

15. If they performed quantitative synthesis, did the review authors carry out an adequate investigation of publication bias (small study bias) and discuss its likely impact on the results of the review?

16. Did the review authors report any potential sources of conflict of interest, including any funding they received for conducting the review?

### Results of individual studies and meta-analyses and quality of evidence

For reviews in which it was not possible to perform meta-analysis, or where the meta-analysis included RCTs not addressing preemptive use only, the results were described based on the main findings of the studies (Table 1).

The results of meta-analyses are presented in Table 3. Falci et al. (2017), Almeida et al. (2019), Caporossi et al. (2020), Almadhoon et al. (2022), and Canellas et al. (2022) evaluated the use of corticosteroids, while Caporossi et al. (2020) and Cetira Filho et al. (2020) assessed the use of NSAIDs.

**TABLE 3:** Results of meta-analysis on the use of corticosteroids (*n* = 5 reviews) and NSAIDs (*n* = 2 reviews).

Reference	Outcome (scales)	Number of RCT (Number of participants)	Meta-analysis (95% CI) I^2^	Study results
Corticosteroids vs. corticosteroids (oral route) undergoing third molar surgery				
Falci et al. (2017)	Trismus reduction	RCT = 2 (*n* = 41)	SMD = 0.29 (−0.04–0.73)	Dexamethasone was not superior to methylprednisolone
	Dexamethasone (8 mg) vs. methylprednisolone (40 mg)			Publication bias = NR
	Follow-up: 2 days		I^2^ = 0%	GRADE = NR
	Trismus reduction	RCT = 2 (*n* = 41)	SMD = 0.62 (0.18–1.07)	Dexamethasone was superior to methylprednisolone
	Dexamethasone (8 mg) vs. methylprednisolone (40 mg)			Publication bias = NR
	Follow-up: 4 days		I^2^ = 0%	GRADE = NR
	Edema/swelling reduction	RCT = 2 (*n* = 41)	SMD = −0.83 (−1.28 to −0.38)	Dexamethasone was superior to methylprednisolone
	Dexamethasone (8 mg) vs. methylprednisolone (40 mg)		I^2^ = 0%	Publication bias = NR
	Follow-up: 2 days			GRADE = NR
	Edema/swelling reduction	RCT = 2 (*n* = 41)	SMD = −1.18 (−1.65 to −0.71)	Dexamethasone was superior to methylprednisolone
	Dexamethasone (8 mg) vs. methylprednisolone (40 mg)		I^2^ = 0%	Publication bias = NR
	Follow-up: 4 days			GRADE = NR
Corticosteroids vs. placebo (oral route) undergoing third molar surgery				
Magesty (2021)	Edema/swelling reduction	NR	DM = −0.65 (−1.24 to −0.06)	Dexamethasone was superior to methylprednisolone
	Dexamethasone (8 mg) vs. placebo		I^2^ = NR	Publication bias = NR
	Follow-up: 1 day			GRADE = high
	Edema/swelling reduction	NR	DM = −1.23 (−2.23 to −0.23)	Dexamethasone was superior to methylprednisolone
	Dexamethasone (8 mg) + nimesulide (100 mg) vs. placebo		I^2^ = NR	Publication bias = NR
	Follow-up: 1 day			GRADE = low
	Reduction in average consumption of analgesics	NR	DM = −2.40 (−4.69 to −0.11)	Dexamethasone was superior to methylprednisolone
	Dexamethasone (4 mg) vs. placebo		I^2^ = NR	Publication bias = NR
				GRADE = NR
	Reduction in average consumption of analgesics	NR	DM = −2.10 (−3.54 to −0.65)	Dexamethasone was superior to methylprednisolone
	Dexamethasone (8 mg) vs. placebo		I^2^ = NR	Publication bias = NR
				GRADE = NR
	Reduction in average consumption of analgesics	NR	DM = −3.10 (−5.02 to −1.17)	
	Dexamethasone (8 mg) + nimesulide (100 mg) vs. placebo		I^2^ = NR	
Corticosteroids vs. placebo (oral, submucosal, intramuscular, and intravenous routes) undergoing third molar surgery				
Almeida et al. (2019)	Postoperative pain relief (VAS scale)	RCT = 4	SMD = −19.58	Corticosteroids were superior to placebo
	Dexamethasone (4 and 8 mg) or methylprednisolone (40, 60 and 80 mg) vs. placebo	(*n* = 194)	(−34.36 to −4.81)	Publication bias = NR
	Follow-up: 1 day		I^2^ = 91%	GRADE = NR
	Trismus reduction	RCT = 5	SMD = 5.58 (2.96–8.20)	Corticosteroids were superior to placebo
	Dexamethasone (4 and 8 mg) or methylprednisolone (40, 60 and 80 mg) vs. placebo	(*n* = 234)	I^2^ = 73%	Publication bias **=** NR
	Follow-up: 2–3 days			GRADE **=** NR
Almadhoon et al. (2022)	Postoperative pain relief (VAS scale)	RCT = 14	MD = −2.95 (−3.58 to −0.42)	Dexamethasone 4 mg was superior to placebo
	Dexamethasone (4 mg, IM) vs. placebo		I^2^ = NR	Publication bias = none
	Follow-up: 1 day	(*n* = 1,980)		GRADE = low
	Postoperative pain relief (VAS scale)		MD = −1.68 (−3.02 to −0.33)	Dexamethasone 4 mg was superior to placebo
	Dexamethasone (4 mg, submucosal) vs. placebo		I^2^ = NR	Publication bias = none
	Follow-up: 1 day			GRADE = low
	Postoperative pain relief (VAS scale)		MD = −1.36 (−2.49 to −0.24)	Dexamethasone 4 mg was superior to placebo
	Dexamethasone (4 mg, admixed with local anesthetic (twin-mix) vs. placebo		I^2^ = NR	Publication bias = none
	Follow-up: 1 day			GRADE = low
	Postoperative pain relief (VAS scale)	RCT = 10	MD = −2.40 (−3.37 to −1.43)	Dexamethasone 4 mg was superior to placebo
	Dexamethasone (4 mg, submucosal) vs. placebo		I^2^ = NR	Publication bias = none
	Follow-up: 3 days	(*n* = 718)		GRADE = low
	Postoperative pain relief (VAS scale)		MD = −2.09 (−3.24 to −0.93)	Dexamethasone 4 mg was superior to placebo
	Dexamethasone (4 mg, IM) vs. placebo		I^2^ = NR	Publication bias = none
	Follow-up: 3 days			GRADE = low
	Postoperative pain relief (VAS scale)		MD = −1.89 (−3.40 to −0.38)	Dexamethasone 4 mg was superior to placebo
	Dexamethasone (4 mg, IV) vs. placebo		I^2^ = NR	Publication bias = none
	Follow-up: 3 days			GRADE **=** low
	Postoperative pain relief (VAS scale)		MD = −1.69; (−3.17 to −0.21)	Dexamethasone 4 mg was superior to placebo
	Dexamethasone (4 mg, oral) vs. placebo		I^2^ = NR	Publication bias = none
	Follow-up: 3 days			GRADE = low
	Trismus reduction	RCT = 6	MD = 2.86 (2.07–3.66)	Corticosteroids were superior to placebo
	Dexamethasone (4 mg, IM) vs. placebo		I^2^ = NR	Publication bias = none
	Follow-up: 1 day	(*n* = 480)		GRADE = moderate
	Trismus reduction		MD = 2.84 (1.89–3.79)	Dexamethasone 4 mg was superior to placebo
	Dexamethasone (4 mg, IV) vs. placebo		I^2^ = NR	Publication bias = none
	Follow-up: 1 day			GRADE = moderate
	Trismus reduction		MD = 2.46 (1.46–3.46)	Dexamethasone 4 mg was superior to placebo
	Dexamethasone (4 mg, submucosal) vs. placebo		I^2^ = NR	Publication bias = none
	Follow-up: 1 day			GRADE = low
	Trismus reduction		MD = 2.45 (1.91–2.99)	Dexamethasone 4 mg was superior to placebo
	Dexamethasone (4 mg, admixed with local anesthetic) vs. placebo		I^2^ = NR	Publication bias = none
	Follow-up: 1 day			GRADE = low
	Trismus reduction		MD = 2.31 (0.02–4.60)	Dexamethasone 4 mg was superior to placebo
	Dexamethasone (4 mg, oral) vs. placebo		I^2^ = NR	Publication bias = none
	Follow-up: 1 day			GRADE = low
	Trismus reduction		MD = 1.70 (0.32–3.07)	Dexamethasone 4 mg was superior to placebo
	Dexamethasone (4 mg, oral) vs. placebo		I^2^ = NR	Publication bias = none
	Follow-up: 3 days			GRADE = low
	Trismus reduction	RCT = 5	MD = 1.63 (0.13–3.12)	Dexamethasone 4 mg was superior to placebo
	Dexamethasone (4 mg, admixed with local anesthetic) vs. placebo		I^2^ = NR	Publication bias = none
	Follow-up: 3 days			GRADE = very low
	Trismus reduction	(*n* = 202)	MD = 2.84 (0.69–4.99)	Dexamethasone 4 mg was superior to placebo
	Dexamethasone (4 mg, submucosal) vs. placebo		I^2^ = NR	Publication bias = none
	Follow-up: 3 days			GRADE = low
	Trismus reduction		MD = 2.40 (0.03–4.77)	Dexamethasone 4 mg was superior to placebo
	Dexamethasone (4 mg, IV) vs. placebo		I^2^ = NR	Publication bias = none
	Follow-up: 3 days			GRADE = low
	Edema/swelling reduction	RCT = 6	MD = −4.15 (−5.46 to −2.84)	Dexamethasone 4 mg was superior to placebo
	Dexamethasone (4 mg, admixed with local anesthetic) vs. placebo		I^2^ = NR	Publication bias = none
	Follow-up: 1 day			GRADE = low
	Edema/swelling reduction		MD = −4.96 (−7.01 to −2.91)	Dexamethasone 4 mg was superior to placebo
	Dexamethasone (4 mg, IV) vs. placebo		I^2^ = NR	Publication bias = none
	Follow-up: 1 day			GRADE = low
	Edema/swelling reduction	(*n* = 480)	MD = −4.88 (−6.96 to −2.80)	Dexamethasone 4 mg was superior to placebo
	Dexamethasone (4 mg, submucosal) vs. placebo		I^2^ = NR	Publication bias = none
	Follow-up: 1 day			GRADE = low
	Edema/swelling reduction		MD = −4.68 (−6.90 to −2.46)	Dexamethasone 4 mg was superior to placebo
	Dexamethasone (4 mg, IM) vs. placebo		I^2^ = NR	Publication bias = none
	Follow-up: 1 day			GRADE = low
	Edema/swelling reduction		MD = −4.41 (−6.51 to −2.30)	Dexamethasone 4 mg was superior to placebo
	Dexamethasone (4 mg, oral) vs. placebo		I^2^ = NR	Publication bias = none
	Follow-up: 1 day			GRADE = low
	Edema/swelling reduction		MD = −5.86 (−8.40 to −3.33)	Dexamethasone 8 mg was superior to placebo
	Dexamethasone (8 mg, oral) vs. placebo		I^2^ = NR	Publication bias = none
	Follow-up: 1 day			GRADE = low
	Edema/swelling reduction	RCT = 5	MD = −6.39 (−8.60 to −4.19)	Dexamethasone 4 mg was superior to placebo
	Dexamethasone (4 mg, admixed with local anesthetic) vs. placebo	(*n* = 202)	I^2^ = NR	Publication bias = none
	Follow-up: 3 days			GRADE = low
	Edema/swelling reduction		MD = −6.50 (−9.70 to −3.30)	Dexamethasone 4 mg was superior to placebo
	Dexamethasone (4 mg, IM) vs. placebo		I^2^ = NR	Publication bias = none
	Follow-up: 3 days			GRADE = low
	Edema/swelling reduction		MD = −6.50 (−9.67 to −3.33)	Dexamethasone 4 mg was superior to placebo
	Dexamethasone (4 mg, IV) vs. placebo		I^2^ = NR	Publication bias = none
	Follow-up: 3 days			GRADE = low
	Edema/swelling reduction		MD = −7.12 (−10.09 to −4.16)	Dexamethasone 4 mg was superior to placebo
	Dexamethasone (4 mg, submucosal) vs. placebo		I^2^ = NR	Publication bias = none
	Follow-up: 3 days			GRADE = low
	Edema/swelling reduction		MD = −6.30 (−9.51 to −3.09)	Dexamethasone 4 mg was superior to placebo
	Dexamethasone (4 mg, oral) vs. placebo		I^2^ = NR	Publication bias = none
	Follow-up: 3 days			GRADE = low
Corticosteroids vs. placebo (oral, submucosal, intramuscular, and intravenous routes) undergoing periodontal surgery				
Caporossi et al. (2020)	Postoperative pain relief (VAS scale)	RCT = 4 (*n* = 254)	SMD = −0.56 (−0.97 to −0.16)	Dexamethasone was superior to placebo
	Dexamethasone 4 mg or 8 mg vs. placebo		I^2^ = 0%	Publication bias = NR
	Follow-up: 1 h			GRADE = moderate
	Postoperative pain relief (VAS scale)	RCT = 4 (*n* = 254)	SMD = −0.39 (−0.87 to 0.08)	Dexamethasone was not superior to placebo
	Dexamethasone 4 mg or 8 mg vs. placebo		I^2^ = 18%	Publication bias = NR
	Follow-up: 4 h			GRADE = moderate
	Postoperative pain relief (VAS scale)	RCT = 4 (*n* = 254)	SMD = −0.51 (−0.88 to −0.14)	Dexamethasone was superior to placebo
	Dexamethasone 4 mg or 8 mg vs. placebo		I^2^ = 3%	Publication bias = NR
	Follow-up: 8 h			GRADE = moderate
NSAIDs vs. placebo or corticosteroids (oral route) undergoing third molar surgery				
Cetira Filho et al. (2020) ^a^	Postoperative pain relief (VAS scale)	RCT = 2 (*n* = 196)	SMD = −1.40 (−2.57 to −0.23)	NSAIDs were superior to placebo or dexamethasone
	Ibuprofen (400 mg) or rofecoxib (50 mg) or diclofenac (50 mg) vs. placebo or dexamethasone 8 mg		I^2^ = 42%	Publication bias = low
	Follow-up: 1 h			GRADE = moderate
	Postoperative pain relief (VAS scale)	RCT = 4 (*n* = 151)	SMD = −1.89 (−2.81 to 0.97)	NSAIDs were not superior to placebo or dexamethasone
	Ibuprofen (400 mg) or etoricoxib (50 and 120 mg) or diclofenac (5 mg) vs. placebo or dexamethasone (8 mg)		I^2^ = 28%	Publication bias = low
	Follow-up: 6 h			GRADE = moderate
	Postoperative pain relief (VAS scale)	RCT = 4 (*n* = 133)	SMD = −0.39 (−1.26 to 0.49)	NSAIDs were not superior to placebo or dexamethasone
	Ibuprofen (400 mg) or etoricoxib (120 mg) or diclofenac (50 mg) or ibuprofen + arginine (400 mg) vs. placebo or dexamethasone (8 mg)		I^2^ = 52%	Publication bias = low
	Follow-up: 1 day			GRADE = moderate
NSAIDs vs. placebo or corticosteroids (oral route) undergoing third molar surgery				
Magesty (2021)	Postoperative pain relief (VAS scale)	NR	MD = −3.95 (−6.63 to −1.27)	Nimesulide was superior to methylprednisolone
	Nimesulide 100 mg vs. placebo		I^2^ = NR	Publication bias = NR
	Follow-up: 12 h			GRADE = high
	Reduction in consumption of rescue medication	NR	MD = −2.40 (−4.22 to −0.58)	Dexamethasone was superior to methylprednisolone
	Etoricoxib (120 mg) vs. placebo		I^2^ = NR	Publication bias = NR
				GRADE = NR
	Reduction in consumption of rescue medication	NR	MD = −5.75 (−7.92 to −3.58)	Dexamethasone was superior to methylprednisolone
	Ibuprofen (600 mg) vs. placebo		I^2^ = NR	Publication bias = NR
				GRADE = NR
	Reduction in consumption of rescue medication	NR	MD = −6.90 (−9.10 to −4.70)	Dexamethasone was superior to methylprednisolone
	Nimesulide (100 mg) vs. placebo		I^2^ = NR	Publication bias = NR
				GRADE = NR
	Edema/swelling reduction	NR	MD = −1.06 (−1.78 to −0.34	Dexamethasone was superior to methylprednisolone
	Diclofenac (50 mg) + codeine (50 mg) vs. placebo		I^2^ = NR	Publication bias = NR
	Follow-up: 1 day			GRADE = low
	Trismus reduction	NR	MD = −6.30 (−7.79 to −4.81)	Dexamethasone was superior to methylprednisolone
	Ampiroxicam (27 mg) vs. placebo		I^2^ = NR	Publication bias = NR
	Follow-up: 1 day			GRADE = moderate
	Trismus reduction	NR	MD = −1.50 (−2.77 to −0.23)	Dexamethasone was superior to methylprednisolone
	Diclofenac 25 mg vs. placebo		I^2^ = NR	Publication bias = NR
	Follow-up: 1 day			GRADE = moderate
NSAIDs vs. placebo or corticosteroid (oral, submucosal, intramuscular, and intravenous routes) undergoing ^b^periodontal surgery				
Caporossi et al. (2020)	Postoperative pain relief (VAS scale)	RCT = 6 (284/284)	SMD = −7.34 (−11.38 to −3.40)	NSAIDs were superior to placebo
	Etoricoxib (120 mg) or celecoxib (200 mg) or ibuprofen (400 mg) or ketorolac (10 and 20 mg) vs. placebo		I^2^ = 33%	Publication bias = NR
	Follow-up: 1 h			GRADE = moderate
	Postoperative pain relief (VAS scale)	RCT = 5 (157/157)	SMD = −17.38 (−27.80 to −6.96)	NSAIDs were superior to placebo
	Etoricoxib (120 mg) or celecoxib (200 mg) or ibuprofen (400 mg) or ketorolac (10 e 20 mg) vs. placebo		I^2^ = 84%	Publication bias = NR
	Follow-up: 4 h			GRADE = moderate
	Postoperative pain relief (VAS scale)	RCT = 4 (139/139)	SMD = −6.69 (−10.92 to −2.45)	NSAIDs were superior to placebo
	Etoricoxib (120 or celecoxib (200 mg) or ketorolac (10 and 20 mg) vs. placebo		I^2^ = 0%	Publication bias = NR
	Follow-up: 8 h			GRADE = moderate
	Postoperative pain relief (VAS scale)	RCT = 3 (96/96)	SMD = −2.72 (−7.15 to 1.70)	AINES were not superior to placebo
	Etoricoxib (120 mg) or celecoxib (200 mg) vs. placebo		I^2^ = 0%	Publication bias = NR
	Follow-up: 8 h			GRADE = moderate
	Postoperative pain relief (VAS scale)	RCT = 3 (136 in total)	SMD = 0.05 (1.02–1.13)	NSAIDs were superior to placebo or dexamethasone
	Ibuprofen (400 mg), etoricoxib (120 mg), rofecoxib (50 mg), or diclofenac (400 mg) vs. placebo or dexamethasone (8 mg)		I^2^ = 55%	Publication bias = low
	Follow-up: 2 days			GRADE = moderate
	Postoperative pain relief (VAS scale)	RCT = 2	SMD = −0.13 (−0.60 to 0.34)	NSAIDs were not superior to dexamethasone
	Etoricoxib (120 mg) or celecoxib (200 mg) vs. dexamethasone (4 and 8 mg)	(70/70)	I^2^ = 0%	Publication bias = NR
	Follow-up: 1 h			GRADE = moderate
	Postoperative pain relief (VAS scale)	RCT = 2	SMD = −0.39 (−0.87 to 0.08)	NSAIDs were not superior to dexamethasone
	Etoricoxib (120 mg) or celecoxib (200 mg) vs. dexamethasone (4 and 8 mg)	(70/70)	I^2^ = 0%	Publication bias = NR
	Follow-up: 4 h			GRADE = moderate
	Postoperative pain relief (VAS scale)	RCT = 2 (70/70)	SMD = 0.01 (−0.67 to −0.69)	NSAIDs were not superior to dexamethasone
	Etoricoxib (120 mg) or celecoxib (200 mg) vs. dexamethasone (4 and 8 mg)		I^2^ = 51%	Publication bias = NR
	Follow-up: 8 h			GRADE = low

NSAIDs, non-steroidal anti-inflammatory drugs; VAS, visual analog scale; NR, not reported; 95% CI, 95% confidence interval; SMD, standardized; I^2^, heterogeneity; MD, mean difference; RCT, randomized clinical trial.

^a^ Pain outcome was collected from systematic reviews with meta-analyses for up to 48 h follow-up and trismus outcome for up to 4-day postoperative follow-up.

^b^ Participants undergoing periodontal surgery.

The results were described by the type of dental procedure (third molar, implant, or periodontal surgery), class of drug (corticosteroids or NSAIDs), and route of administration (oral; submucosal; intramuscular, IM; or intravenous, IV).

#### Corticosteroids in third molar surgery (*n* = 10 systematic reviews)

**Oral route:** Meta-analyses showed that dexamethasone (8 mg) was superior to methylprednisolone (40 mg) for reducing trismus and swelling within 4 days (quality of evidence was not reported) (Table 3). However, the findings proved insufficient to state that the use of preemptive dexamethasone was superior to methylprednisolone or NSAIDs (Falci et al., 2017).

Meta-analyses evaluated the preemptive oral use of anti-inflammatory drugs compared to placebo. There was reduction in pain with the use of nimesulide (100 mg) up to 24 h after surgery (high quality of evidence). Reduction in edema was also observed with the use of dexamethasone (8 mg) (high quality of evidence) and diclofenac (50 mg). A reduction in trismus was observed with the use of ampiroxicam (27 mg) and diclofenac (25 mg), both on the first day after surgery. Reduction in the average consumption of analgesics was also attributed to the use of nimesulide (100 mg), dexamethasone (4 and 8 mg), etoricoxib (120 mg), and ibuprofen (600 mg) compared to placebo. The study collected no data on adverse effects. The quality of evidence, when reported, was low or moderate (Table 3) (Cetira Filho et al., 2020).

**More than one route:** A meta-analysis evaluated the use of corticosteroids compared to placebo (via oral, submucosal, IM, or IV routes). Dexamethasone (4 and 8 mg) and methylprednisolone (40, 60, and 80 mg) both showed superior results to placebo for pain relief (within 24 h) and trismus reduction (within 72 h after surgery). The quality of evidence was not reported (Table 3). The route of administration appeared not to influence results, with the oral route representing the easiest option (Almeida et al., 2019).

Indirect meta-analyses observed that dexamethasone (4 mg, pterygomandibular or submandibular routes), dexamethasone (8 mg, pterygomandibular or submandibular), triamcinolone (4 mg, submandibular), and methylprednisolone (40, 80 mg, oral) were more effective than placebo for reducing pain on the first day after surgery. Dexamethasone (0.1 mg/kg, IM route), dexamethasone (4 mg, submandibular), dexamethasone (8 mg, submandibular, IM, or IV), and methylprednisolone (40 mg, oral or submandibular) were more effective than placebo for this outcome on the second day after surgery (Canellas et al., 2022).

Dexamethasone (8 mg, submucosal or pterygomandibular routes) was more effective for reducing edema after 2 days of surgery. With regard to reducing trismus, dexamethasone (4 mg, submandibular route) and methylprednisolone (125 mg, IV route) were more effective than placebo 7 days postoperatively. Safety data were poorly reported by clinical trials, but no serious complications were attributed to the use of corticosteroid (Table 3). Overall, corticosteroids reduced inflammatory complications, where dexamethasone (8 mg) appeared to be the best preoperative option for controlling these events. However, further RCTs should be performed to improve the quality of evidence (Canellas et al., 2022).

Indirect meta-analyses observed that dexamethasone (4 mg, IM or submucosal or local anesthetic twin-mixed routes), compared to placebo, reduced pain outcome on the first day after surgery and dexamethasone (4 mg, oral, IM, submucosal, or IV) reduced pain on the third day after surgery. Trismus measurements were reduced with the use of dexamethasone (4 mg, IM, IV, submucosal, or admixed with local anesthetic) and of dexamethasone (4 and 8 mg, oral) compared to placebo on the first day after surgery. On the third day after surgery, dexamethasone (4 mg, IV, submucosal, or administered with local anesthetic) also reduced trismus compared to placebo. Swelling measurements were reduced after the use of dexamethasone (4 mg, IM, IV, submucosal, or admixed with local anesthetic) and of dexamethasone (4 and 8 mg, oral) compared to placebo 1 day postoperatively. Three days after surgery, swelling was reduced with the use of dexamethasone (4 mg, oral, IM, IV, submucosal, or admixed with local anesthetic) compared to placebo. However, the quality of evidence was low for most of the findings (Table 3). Safety data were not reported by Cetira Filho et al. (2020).

Dexamethasone (4 mg) and prednisolone (10 mg) were compared to dexamethasone (8 mg), prednisolone (20 mg), or placebo via the oral, submucosal, and IM routes. According to the authors, although corticosteroids reduced edema and trismus compared to placebo, the effects on pain reduction remained a topic for further investigation (Varvara et al., 2017).

Methylprednisolone (16, 40, 60, and 80 mg) via the oral, submucosal, and IV routes was evaluated. Oral methylprednisolone reduced pain and trismus in the early postoperative period and reduced late postoperative pain compared to placebo. The reported effects of submucosal methylprednisolone (40 mg) on pain and trismus were conflicting. Methylprednisolone (20, 40, 80, and 125 mg, by IV) had no significant effect on pain and trismus but reduced edema (Nagori et al., 2019).

Dexamethasone (4 and 8 mg), methylprednisolone (16, 20, 40, and 125 mg), and prednisolone (10 and 20 mg), by oral and IV routes, were compared to placebo. In general, these corticosteroids reduced trismus and swelling, promoting a superior effect for the IV route to the oral route (Herrera-Briones et al., 2013).

Betamethasone (60 mg, IM route), dexamethasone (4 mg, IV), and methylprednisolone (40, 80, and 125 mg, IV) were compared to placebo. Data from meta-analyses were not collected, since the review summarized the results of studies that evaluated the pre- and postoperative uses of these drugs. The findings showed that corticosteroids promoted mild-to-moderate reduction in early edema and trismus, but the confidence in findings was limited due to the small number of trials ^24^.

Dexamethasone (8 mg) and methylprednisolone (40 mg) (both IV route) compared to placebo had limited impact on the quality of life. Dexamethasone (4 mg, submucosal route) reduced pain (2nd to 10th days postoperatively) and swelling (2nd day postoperatively) compared to placebo. No significant group difference in the reduction of trismus was observed, and no adverse effects were reported (Parhizkar et al., 2022).

#### NSAIDs in third molar surgery (*n* = 6 systematic reviews)

**Oral route:** Meta-analyses showed that preemptive analgesia with ibuprofen (400 mg), rofecoxib (50 mg), or diclofenac (50 mg) during the first 6 h postoperatively was superior to placebo or dexamethasone (8 mg) (moderate-quality evidence). NSAIDs (diclofenac 50, 100, or 150 mg; etoricoxib 90 mg; ibuprofen 400 and 800 mg; ibuprofen 600 mg + arginine 555 and 120 mg; and ketoprofen 100 and 150 mg) were associated with lower consumption of rescue medication than placebo, dexamethasone (8 mg), or codeine (30 mg) (moderate-quality evidence) (Table 3). The side effects most reported by the study were drowsiness, dizziness, headache, nausea, vomiting, trembling, sleepiness, allergy, syncope, and dyspnea (Cetira Filho et al., 2020).

Celecoxib (200 mg) was compared to ibuprofen (400 and 600 mg), acetaminophen (500 mg), and placebo at 4, 8, 24, and 48 h postoperatively. Celecoxib demonstrated better analgesic activity, with a lower number of patients requiring rescue analgesia than for the other drugs and placebo. The adverse effects reported were nausea (25%, 8.3%, and 18.1%), headache (14.1%, 9.2%, and 8.7%), and vomiting (11.3%, 1.3%, and 9.1%), as observed in placebo, celecoxib, and ibuprofen groups, respectively. Celecoxib had lower postoperative pain scores compared to acetaminophen, up until 12 h postoperatively. The number of patients requiring a rescue analgesic was lower in the celecoxib group than in the acetaminophen group (Isiordia-Espinoza et al., 2022).

**IV route:** The systematic review included one clinical trial that evaluated the preemptive use of parecoxib in third molar surgery. Parecoxib (20, 40, and 80 mg) was more effective for the management of postoperative pain than placebo. No reports of gastrointestinal, hematological, or renal adverse effects were found in the study (Romsing and Moiniche, 2004).

**Oral and IM routes:** Meloxicam (7.5, 10, and 15 mg, oral route) and meloxicam (7.5 and 15 mg, IM) were evaluated for postoperative pain management in patients undergoing third molar surgery. Meloxicam (any dose) had similar analgesic effects to naproxen (550 mg), diflunisal (500 mg), acetaminophen (500 mg), rofecoxib (12.5 mg), and nimesulide (100 mg) and a superior analgesic effect compared to ampiroxicam (27 mg), diclofenac (100 mg), salicylates, and tramadol (Khosraviani et al., 2020).

**IM and IV routes:** Ketorolac (30 mg, IM or IV routes) was compared to placebo or other drugs. Improvements in postoperative pain, median time taken for rescue medication, the total number of analgesics taken, and overall patient satisfaction were observed with the use of ketorolac (30 mg, IM) compared to diclofenac (75 mg, IM) and tramadol (50 mg, IV). However, further studies with a larger sample size are needed to inform best practices in this domain (Tirupathi et al., 2021).

The systematic review included one clinical trial which found that the preemptive use of ibuprofen (800 mg) + dexketoprofen (50 mg) or ibuprofen 800 mg (both IV route) reduced pain compared to placebo, within 48 h postoperatively. No adverse effects were reported (Silva et al., 2021).

#### Corticosteroids and NSAIDs in periodontal surgery (*n* = 2 systematic reviews)

Meta-analyses showed a superior effect of dexamethasone (4 and 8 mg, oral route) compared to placebo for controlling postoperative pain up to 8 h after surgery (moderate quality of evidence). There was also a superior effect of oral drugs etoricoxib (120 mg), celecoxib (200 mg), or ketorolac (10 and 20 mg) compared to placebo for controlling postoperative pain up to 8 h after the procedure (moderate quality of evidence) (Table 3). Regarding the occurrence of side effects, patients had no adverse effects after the use of analgesics in 16 studies. When side effects were reported, these were generally mild and equally distributed among treatment groups. The most frequently reported adverse effects were drowsiness, nausea, headache, and dizziness (Caporossi et al., 2020).

Another review included one clinical trial which showed that oral preemptive ketorolac 20 mg reduced pain compared to placebo. No difference was observed between the groups regarding the use of rescue medication (Nir et al., 2016).

#### Oral corticosteroids in implant surgery (*n* = 1 systematic review)

Of the 11 clinical trials evaluating the use of analgesics after dental implant, two studies were specifically on oral preemptive use. The results showed that ibuprofen (600 mg) and dexketoprofen (25 mg) were superior to placebo for reducing pain. Further RCTs with an adequate sample size comparing standardized implant approaches are needed to inform best practices in this domain (Melini et al., 2021).

### Main findings on randomized clinical trials included

Of the total 203 RCTs identified within systematic reviews, 93 (45.8%) were included in more than one review. Data on the clinical trials included in systematic reviews (without overlap = 110) are described in Supplementary Materials SC, SD. Data on interventions, comparators, follow-up time, and main effectiveness and safety outcomes were extracted, with this information collected from reviews and RCTs, when necessary.

A total of 70 clinical trials evaluated the use of corticosteroids: dexamethasone by oral (*n* = 20), submucosal (*n* = 17), pterygomandibular (*n* = 4), IM (*n* = 9), and IV (*n* = 5) routes; methylprednisolone by oral (*n* = 3), IM (*n* = 3), and IV (*n* = 6) routes; and oral prednisolone (*n* = 3) (Supplementary Material SC).

A total of 40 clinical trials evaluating the use of NSAIDs are described as follows: ibuprofen by oral (*n* = 7) and IV (*n* = 1) routes; ketorolac by oral (*n* = 2), IM (*n* = 1), and IV (*n* = 3) routes; meloxicam by oral (*n* = 3) and IM (*n* = 2) routes; diclofenac by oral (*n* = 4) and IM (*n* = 1) routes; oral diflunisal (*n* = 2); oral nimesulide (*n* = 2); and oral celecoxib (*n* = 3). Oral acetaminophen was evaluated in three RCTs. Dexketoprofen, rofecoxib, tenoxicam, parecoxib, and tramadol were evaluated by only one RCT (Supplementary Material SD).

## Discussion

### General interpretation of the results in the context of other evidence

The present review summarized the available evidence on the preemptive use of anti-inflammatory and analgesic drugs in oral surgery. Third molar surgery was the dental procedure most investigated. The reviews evaluated the use of corticosteroids and NSAIDs, where none of the studies were restricted to the use of opioids and acetaminophen. The oral route was involved in most studies. Furthermore, the majority of the reviews had at least two critical methodological flaws. The outcomes most reported included pain, edema, and trismus, while safety findings were rarely reported by the reviews.

Based on the review results, patients can benefit from the preemptive use of betamethasone (10, 20, and 60 mg), dexamethasone (4 and 8 mg), methylprednisolone (16, 20, 40, 60, 80, and 125 mg), or prednisolone (10 and 20 mg) by different routes (oral, pterygomandibular, submandibular, submucosal, IM, or IV) to minimize postoperative inflammatory complications after third molar surgery. However, the reviews reported the need for further RCTs, given the wide variety of drugs, doses, and routes of administration used, as well as issues with the methodological quality of studies, limiting the reliability of their findings. Only 3 out of the 10 reviews on corticosteroids reported information on adverse reactions, and no serious adverse effects were associated with the use of these drugs.

Celecoxib (200 mg), diclofenac (25, 30, 50, 75, and 100 mg), etoricoxib (120 mg), ibuprofen (400 and 600 mg), ketorolac (30 mg), meloxicam (7.5, 10, and 15 mg), nimesulide (100 mg), and rofecoxib (50 mg) were studied for different routes (oral, IM, and IV) and compared to corticosteroids, other NSAIDs, placebo, and tramadol. These drugs appear to be effective mainly for reducing pain (outcome most reported by studies) in patients undergoing third molar surgery. Although six systematic reviews with NSAIDs reported adverse effects data, information was sparse. No serious adverse effects were reported with the preemptive use of NSAIDs.

Celecoxib (200 mg), dexamethasone (4 and 8 mg), etoricoxib (120 mg), and ketorolac (10 and 20 mg), administered by oral or submucosal or IM or IV routes, reduced postoperative pain compared to placebo and were considered safe for use in patients undergoing periodontal surgery. In implant surgery, findings for the preemptive use of drugs were available only for ibuprofen (600 mg) and dexketoprofen (25 mg). Both drugs were shown to reduce postoperative pain, although the reliability of these findings needs to be confirmed.

The literature search identified an overview of systematic reviews that summarized the available evidence on the effectiveness and safety of opioid and non-opioid analgesics in acute dental pain in a population which included children (Moore et al., 2018). In this study, corticosteroids were not studied, and the combination of ibuprofen and acetaminophen showed the greatest treatment benefit in pain reduction. Diflunisal, acetaminophen, and oxycodone had the longest duration of action in adult patients. The present study updated part of the findings of this review and also included corticosteroids, drugs widely used in dentistry.

### Study strengths and limitations

The present study entailed a comprehensive literature search in which all stages of selection and data extraction were performed by reviewers, in pairs and independently. There was no restriction on the language of publication with respect to the reviews included. Although the quality of the evidence reported was based on information provided by the reviews and, hence, might have been affected by the methodological quality of the studies, the strength of this review was in providing an outline of the state of the art according to the available literature and to highlight information gaps.

It is important to note that adverse drug reactions, quality of life, and rescue medications were described by few systematic reviews, despite being relevant outcomes when assessing interventions. Furthermore, the heterogeneity of the reviews regarding drugs, doses, routes of administration, comparators, follow-up time, and outcomes, as well as the poorly reported quality of evidence, limited the findings of this review.

In general, the certainty of evidence of findings was poorly reported by the systematic reviews. In cases where certainty was evaluated, the quality of evidence tended to be rated as low or moderate, limiting the reliability of findings of the present review.

### Implications for clinical practice and future research

Preemptive analgesia in oral surgery has been widely performed for reduction and control of postoperative inflammatory processes. Most of the reviews included in the present study found that the use of corticosteroids and NSAIDs showed good results for reducing pain, edema, and trismus of patients undergoing dental surgeries. Cetira Filho et al. (2020) and Isiordia-Espinoza et al. (2022) reported the use of rescue medication, showing a reduction in medication consumption. However, the quality of evidence of these findings ranged from low to moderate.

Pain, swelling, and trismus often occur postoperatively and can affect the patient’s quality of life. Because it is a highly vascularized region of the body, there is a large release of exudate and mediators that cause the migration of inflammatory cells to the operated area (Cetira Filho et al., 2020). Oral surgery promotes injury to the surrounding tissues, producing pain, acute inflammation in the masseter muscle and submandibular regions, and trismus (Herrera-Briones et al., 2013). Thus, pharmacological management is designed to control and minimize these inflammatory sequelae.

The wide variety of clinical trials evaluating different drugs, doses, routes of administration, and follow-up times, precluded further analyses of specific or commonly used protocols for preemptive analgesia in dental surgery. Taken together, the findings of this review suggest that further RCTs with rigorous methodological designs should be carried out to provide clinical evidence on dose and administration route of drugs for the preemptive use in oral surgical procedures performed under local anesthesia. This information is valuable for the scientific community and dentists, contributing toward a better management of exacerbated inflammatory responses in intraoral surgical procedures.

## Conclusion

The preemptive use of corticosteroids and NSAIDs can reduce pain, edema, and trismus in oral surgeries, particularly in third molar surgeries, the procedure most studied in the literature reviewed. These drugs also proved to be safe at the doses evaluated, although data on adverse effects were poorly reported. However, given the wide variety of drugs, doses, administration routes, and follow-up times, coupled with the low or moderate quality of evidence, further RCTs should be conducted to confirm these findings. The information can help guide the decision-making of patients and dentists on the use of drugs for preemptive analgesia and pave the way for future scientific studies defining more precise protocols for the preemptive use of these drugs in dental surgeries.

## Data Availability

The original contributions presented in the study are included in the article/Supplementary Material; further inquiries can be directed to the corresponding author.
