# Erratum: Preemptive use of anti-inflammatories and analgesics in oral surgery: a review of systematic reviews

**DOI:** 10.3389/fphar.2024.1430168

**Published:** 2024-05-24

**Authors:** Frontiers Production Office

**Affiliations:** Frontiers Media SA, Lausanne, Switzerland

**Keywords:** analgesics, oral surgical procedures, preoperative period, postoperative pain, anti-inflammatory

Due to a production error, Table 1 was erroneously omitted from the published article. Moreover, Table 1 was incorrectly cited in the text as **Supplementary Table 1**. The correct Table 1 and its caption appear below. For this reason the original Table 1 (Risk of bias of systematic reviews included (*n* = 19)) is now renamed as **Table 2**, and the original **Table 2** (Results of meta-analysis on the use of corticosteroids (*n* = 5 reviews) and NSAIDs (*n* = 2 reviews)) is now renamed as **Table 3**.

**TABLE 1:** Characteristics of systematic reviews on preemptive use of anti-inflammatory drugs and analgesics in dental procedures (*n* = 19).

References (search period)	Number of preemptive RCT	Age of population (years)	Intervention vs. comparator (route of administration)	Main objective of review	Conclusions of review based on findings of preemptive use of drugs
Third Molar Surgery (Corticosteroids VS. Corticosteroids, Placebo, Or Other Drugs)					
^a^Falci et al., 2017 (up to Apr/2015)	7	15–41 (Min-Max)	Corticosteroids **vs.** corticosteroids or NSAIDS or placebo (*Oral route*)	To assess the effectiveness of preemptive oral use of dexamethasone in lower third-molar extractions in terms of reducing pain, swelling and/or trismus compared with other oral anti-inflammatories	There is insufficient evidence through meta-analysis to conclude that oral dexamethasone is better than methylprednisolone or NSAIDS as a preemptive analgesic. Dexamethasone may be more effective than methylprednisolone for reducing swelling and trismus. The study collected no data on adverse effects
Almadhoon et al., 2022 (up to Sep/2021)	34	18–69 (Min-Max)	Corticosteroids **vs.** corticosteroids or placebo (*Oral, submucosal, intramuscular, and intravenous routes*)	To assess the comparative effects of different dexamethasone routes and doses on reducing postoperative sequelae after impacted mandibular third molars surgery	Dexamethasone, in different doses and routes, was superior to placebo in reducing pain, edema, and trismus, 1 day after surgical extraction. Submucosal dexamethasone 4 mg reduced pain until 3 days after extraction. No noteworthy difference was found between route and dose of dexamethasone. The study collected no data on adverse effects
^a^Canellas et al., 2022 (up to 2021)	42	16–50 (Min-Max)	Corticosteroids **vs.** corticosteroids or placebo (*Oral, submucosal, intramuscular, intravenous, pterygomandibular routes*)	To compare the effects of different corticosteroids to minimize postoperative inflammatory complications after surgical extraction of the third molar by applying a network meta-analysis approach	Corticosteroids reduced inflammatory complications after third molar surgery. Dexamethasone 8 mg could be the best preoperative option to control inflammatory complications; however, more RCTs should be conducted to increase the quality of direct evidence. Few RCTs reported data on safety, but no serious adverse effects were observed. Surgeons should consider the use of dexamethasone submucosal, pterygomandibular, or oral routes to control these inflammatory complications
Parhizkar et al., 2022 (up to May/2021)	2	>15	Corticosteroids **vs.** placebo (*Submucosal and intravenous routes*)	To evaluate the efficacy of adjunctive corticosteroid therapy in improving patient-centered outcomes following third molar surgery	Dexamethasone 8 mg and methylprednisolone 40 mg (both via intravenous route) improved quality of life compared to placebo. Dexamethasone 4 mg (submucosal route) improved pain and swelling compared to placebo. No adverse effects have been reported
^a^Almeida et al., 2019 (up to Apr/2017)	9	15–45 (Min-Max)	Corticosteroids **vs.** placebo (*Oral, submucosal, intramuscular, and intravenous routes*)	To examine the effectiveness of corticosteroids in the control of pain, edema, and trismus following third molar surgery	Corticosteroids had a positive effect with regard to the control of the pain, edema, and trismus in the surgical removal of impacted third molars. The study collected no data on adverse effects. Route of administration appeared not to influence the results, making the oral route an easy excellent option
Varvara et al., 2017 (up to Apr/2017)	6	Not reported	Corticosteroids **vs.** corticosteroids or placebo (*Oral, submucosal and intramuscular routes*)	To examine the different corticosteroids used in oral surgery procedures to define the time and route of administration	The use of corticosteroids in oral surgery is promising for the reduction of postoperative morbidity, edema, and trismus. The drugs’ effects on pain reduction remain a topic for further investigation. The study collected no data on adverse effects
Nagori et al., 2019 (up to Jan/2018)	11	22 ± 4.2 (Mean ± standard deviation)	Corticosteroids **vs.** placebo (*Oral, submucosal, and intravenous routes*)	To investigate the available evidence on whether methylprednisolone improves postoperative outcomes following impacted third molar surgery	Methylprednisolone improved pain and edema in the early postoperative period but had no effect on edema in late postoperative. The study collected no data on adverse effects. More high-quality RCTs are required to provide stronger evidence for the use of this corticosteroid
Herrera-Briones et al., 2013 (up to Sep/2011)	11	Not reported	Corticosteroids **vs.** corticosteroids or placebo (*Oral and intravenous routes*)	To conduct a systematic review on the preemptive use of corticosteroids in third molar surgery	The use of dexamethasone, methylprednisolone, and prednisolone improved the postoperative experience of patients, with a significant impact on trismus and inflammation. The study collected no data on adverse effects. Greater effects appear to be achieved by using the parenteral route
Markiewicz et al., 2008 (up to Mar/2007)	5	23.2 (Mean)	Corticosteroids **vs.** placebo (*Intramuscular and intravenous routes*)	To evaluate the use of perioperative corticosteroids compared to placebo for reducing postoperative edema, trismus, and pain in patients undergoing removal of the third molar	Betamethasone, dexamethasone, and methylprednisolone promoted mild-to-moderate reduction in edema and improvement in range of motion after undergoing removal of third molar compared to placebo. The study collected no data on adverse effects. These findings need to be confirmed
Third Molar Surgery (Nsaids VS Placebo Or Other Drugs)					
^a^Cetera Filho et al., 2020 (up to Mar/2019)	20	22.0 ± 2.9 (Mean ± standard deviation)	NSAIDS or acetaminophen or corticosteroids **vs.** NSAIDS or opioids or placebo (*Oral route*)	To investigate the effectiveness of preemptive analgesia with NSAIDS for the relief of inflammatory events after surgical removal of third molars	Some NSAIDS were effective in controlling pain, edema, and trismus. Preemptive analgesia in the removal of third molars lowered average pain scores, especially in the first 6 h after surgery, and reduced average consumption of medication and the number of patients who needed it postoperatively. In general, the authors of the studies selected reported the main adverse effects as drowsiness, dizziness, headache, nausea, vomiting, trembling, sleepiness, allergy, syncope, and dyspnea
Magesty et al., 2021 (up to Aug/2020)	31	15–45 (Min-Max)	NSAIDS or corticosteroids **vs**. NSAIDS or placebo (*Oral route*)	To compare the effectiveness of oral pre-emptive analgesia administered for mandibular third molar surgery through a network meta-analysis	Reduction in average consumption of analgesics was observed with the use of nimesulide 100mg, dexamethasone 4 mg and 8mg, etoricoxib 120 mg and ibuprofen 600 mg compared to placebo. Reduction in pain was observed with the use of nimesulide 100 mg and reduction in edema with the use of dexamethasone 8 mg and diclofenac 50 mg and reduction in trismus with the use of ampiroxicam 27 mg and diclofenac 25 mg. The study collected no data on adverse effects
Isiordia-Espinosa et al., 2022 (up to Apr/2021)	3	Not reported	NSAIDS vs. NSAIDS vs. placebo (*Oral route*)	To evaluate the analgesic efficacy and adverse effects of celecoxib compared to non-opioid drugs after third molar surgery	Celecoxib 200 mg showed better analgesic efficacy than ibuprofen 400mg, acetaminophen 500 mg and placebo after 4, 8, and 24 postoperative hours following the third molar removal. The number of patients who required rescue analgesia was lower for celecoxib compared to non-opioid drugs. Lower number of gastrointestinal adverse effects with celecoxib compared with non-opioid treatments was observed
Khosraviani et al., 2020 (up to Jun/2018)	6	20–26 (Min-Max)	NSAIDS **vs**. NSAIDS or opioids or placebo (*Oral or intramuscular routes*)	To evaluate the effectiveness of meloxicam on post-operative pain management in patients who have undergone orofacial surgeries	Meloxicam had similar analgesic effects to naproxen 550mg, diflunisal 500mg, acetaminophen 500mg, rofecoxib 12.5 mg and nimesulide 100 mg and superior effects to ampiroxicam 27mg, diclofenac 100 mg and tramadol. One study reported mild nausea and vomiting and allergy as meloxicam-related complications
Silva et al., 2021 (up to Mar/2021)	1	18–35 (Min-Max)	NSAIDS **vs**. placebo (*Intravenous route*)	To assess the effect of preemptive intravenous ibuprofen on pain reduction after lower third molar surgery	Intravenous ibuprofen 800 mg, alone or combined with dexketoprofen, had the same perioperative analgesic efficacy and both were superior to placebo. No adverse effects were reported in the study
Romsing et al., 2004 (up to Jun/2003)	1	Not reported	NSAIDS **vs**. placebo (*Intravenous route*)	To review the analgesic efficacy of COX-2 inhibitor for post-operative pain relief after surgical removal of third molars	Intravenous administration of parecoxib 20mg, 40mg, 80 mg before oral surgery was effective and safe in providing preventive management of postoperative pain compared to placebo. No adverse effects were reported in the study
Tirupathi et al., 2021 (1980 to Jul/2020)	6	16–35 (Min-Max)	NSAIDS **vs**. corticosteroids or NSAIDS or tramadol or placebo (*Intramuscular and intravenous routes*)	To evaluate preemptive injected ketorolac in comparison to other agents for surgical removal of third molars	The studies showed better outcomes efficacy, but definitive conclusions cannot be made regarding the use of injected ketorolac for control of pain and edema. Thus, more clinical trials are needed to make definitive conclusions. In one RCT, four patients had complaints of nausea after receiving tramadol
Periodontal Surgery					
^a^Caporossi et al., 2020^a^ (up to Sep/2019)	5	18–56 (Min-Max)	Corticosteroids and NSAIDS **vs.** corticosteroids or NSAIDS or placebo (*Oral, submucosal, intramuscular, and intravenous routes*)	To systematically review the literature on the pharmacological effect of different drugs on pain relief after periodontal surgeries	Dexamethasone (4mg and 8 mg), etoricoxib (120 mg), celecoxib (200 mg), or ketorolac (10mg and 20 mg) compared to placebo reduced postoperative pain, up to 8 h after the procedure. There is not enough evidence to suggest a standard treatment. The side effects observed were generally mild and equally distributed among treatment groups
Nir et al., 2016^b^ (up to Jan/2015)	1	17–85 (Min-Max)	NSAIDS **vs.** placebo (*Oral route*)	To evaluate the effectiveness of preoperative oral use of ketorolac for reducing analgesic consumption and minimizing postoperative pain	Ketorolac 20 mg reduced pain compared to placebo. No difference was observed between the groups regarding the use of rescue medication. No adverse effects related to preoperative ketorolac use were observed
Implant Surgery					
Melini et al., 2021 (up to May/2020)	2	17–85 (Min-Max)	NSAIDS **vs.** placebo (*Oral route*)	To summarize the available evidence on analgesics in the management of postoperative pain after dental implant placement	Dexketoprofen 25 mg and ibuprofen 600 mg were superior to placebo for reducing pain. One RCT reported bleeding due to the use of dexketoprofen. Further RCTs are needed to inform best practices in this domain

NSAIDS, Non-Steroidal Anti-Inflammatory Drugs. RCT, Randomized Clinical Trial.

^a^ Systematic reviews with specific meta-analysis data on preemptive drug use.

^b^ Non-specific reviews of dental studies.

^c^ Information available only in the review method.

