# Supplementary material for: Preemptive use of anti-inflammatories and analgesics in oral surgery: a review of systematic reviews
Source: Front Pharmacol. 2024 Jan 24;14:1303382. doi: 10.3389/fphar.2023.1303382 (PMC10847331; doi:10.3389/fphar.2023.1303382)
Supplement: Supplementary file 1 [file DataSheet1.docx]

**Supplementary Materials**

**A - Descriptors selected for search in databases and search strategy in databases**

| **#1**  **Dental procedures** | (Maxillofacial Surgery) OR (Oral Surgery) OR Exodontics OR (Third Molar) OR (Third Molars) OR (Wisdom Tooth) OR (Wisdom Teeth) OR (Impacted Tooth) OR (Impacted Teeth) OR (Dental Implant) OR (Dental Implants) OR (Unerupted Tooth) OR (Unerupted Teeth) OR (Supernumerary Tooth) OR (Supernumerary Teeth) OR (Fourth Molar) OR (Fourth Molars) OR (Bone Transplantation) OR (Bone Grafting) OR Autograft OR (Autologous Transplants) OR (Autologous Transplant) OR Autotransplants OR Autotransplant OR Allografts OR Allograft OR (Allogeneic Transplants) OR (Allogeneic Transplant) OR (Allogeneic Grafts) OR (Allogeneic Graft) OR Homografts OR Homograft OR (Homologous Transplants) OR (Homologous Transplant) OR Heterografts OR Heterograft OR Xenografts OR Xenograft OR Pulpitis OR Pulpitides OR (Endodontic Inflammation) OR (Endodontic Inflammations) OR endodontics OR endodontology OR (Dental Pulp Diseases) OR (Dental Pulp Disease) OR (root canal therapy) OR (root canal therapies) OR endodontic OR (periapical periodontitis) OR (Periapical Periodontitides) OR (Apical Periodontitides) OR (Apical Periodontitis) OR (Acute Nonsuppurative Periodontitides) OR (Acute Nonsuppurative Periodontitis) OR (dental pulp necrosis) OR (Dental Pulp Necroses) OR (Dental Pulp Necrosis) OR (Pulp Necroses) OR (Pulp Necrosis) OR (dental pulp gangrene) OR (dental pulp gangrenes) OR (Pulp Gangrene) OR (Pulp Gangrenes) OR (Pulp Mummification) OR (Pulp Mummifications) OR (Dental Pulp Autolysis) OR (Dental Pulp Autolyses) OR (nonvital tooth) OR (tooth devitalized) OR (tooth pulpless) OR (teeth pulpless) OR (teeth devitalized) OR (nonvital teeth) OR (teeth endodontically-treated) OR (teeth endodontically treated) OR (tooth endodontically-treated) OR (tooth endodontically treated) OR (granuloma periapical) OR (granulomas periapical) OR (periapical periodontitis chronic nonsuppurative) OR (periodontitis apical chronic nonsuppurative) OR (dental granulomas) OR (dental granuloma) OR (periodontitis apical suppurative) OR (abscess periapical) OR (abscesses periapical) OR (periapical periodontitides suppurative) OR (alveolar abscess apical) OR (abscesses apical alveolar) OR (dentoalveolar abscess apical) OR (abscesses apical dentoalveolar) OR Pulpectomy OR Pulpectomies OR (root canal preparation) OR (root canal preparations) |
| --- | --- |
| **#2 Drugs** | (Adrenal Cortex Hormones) OR Corticosteroids OR Corticosteroid OR Corticosteroids OR Corticosteroid OR (Glucocorticosteroids) OR Glucocorticosteroid OR (Glucocorticosteroid Effect) OR (Glucorticosteroid Effects) OR (Anti-Inflammatory Agents) OR (Anti Inflammatory Agents) OR (Antiinflammatory Agent) OR (Antiinflammatory Agents) OR Anti-Inflammatories OR (Anti Inflammatories) OR (Antiinflammatories) OR (Anti-Inflammatory Agent) OR (Anti Inflammatory Agent) OR (Agents Anti-Inflammatory) OR (Agents Anti Inflammatory) OR Dexamethasone OR Methylfluorprednisolone OR Betamethasone OR Flubenisolone OR Betadexamethasone OR (Methylprednisolone Acetate) OR Methylprednisolone OR Methylprednisolone-21-acetate OR (Methylprednisolone 21 acetate) OR Acetyl-Methylprednisolone OR (Acetyl Methylprednisolone) OR 6-Methylprednisolone OR (6 Methylprednisolone) OR [Prednisone](https://www.ncbi.nlm.nih.gov/mesh/68011241) OR Dehydrocortisone OR delta-Cortisone OR Prednisolone OR Hidrocortisone OR cortisol OR (Anti-Inflammatory Agents Non-Steroidal) OR NSAID OR (Nonsteroidal Anti-Inflammatory Agent) OR (Agent Nonsteroidal Anti-Inflammatory) OR (Anti-Inflammatory Agent Nonsteroidal) OR (Nonsteroidal Anti Inflammatory Agent) OR NSAIDS OR (Antiinflammatory Agents Non Steroidal) OR (Antiinflammatory Agents Nonsteroidal) OR (Nonsteroidal Antiinflammatory Agents) OR (Non-Steroidal Anti-Inflammatory Agents) OR (Non Steroidal Anti Inflammatory Agents) OR (Nonsteroidal Anti-Inflammatory Agents) OR (Nonsteroidal Anti Inflammatory Agents) OR (Non-Steroidal Anti-Inflammatory Agent) OR (Agent Non-Steroidal Anti-Inflammatory) OR (Anti-Inflammatory Agent Non-Steroidal) OR (Non Steroidal Anti Inflammatory Agent) OR (Anti Inflammatory Agents Nonsteroidal) OR (Analgesics Anti-Inflammatory) OR (Anti-Inflammatory Analgesics) OR (Aspirin-Like Agents) OR (Aspirin Like Agents) OR (Aspirin-Like Agent) OR (Agent Aspirin-Like) OR (Aspirin Like Agent) OR ([Cyclooxygenase 2 Inhibitors](https://www.ncbi.nlm.nih.gov/mesh/68052246)) OR (Cyclooxygenase 2 Inhibitor) OR (COX-2 Inhibitor) OR (COX 2 Inhibitor) OR Coxib OR Coxibs OR (COX-2 Inhibitors) OR (COX 2 Inhibitors) OR (COX2 Inhibitors) OR (COX2 Inhibitor) OR (Cyclooxygenase-2 Inhibitors) OR (Cyclooxygenase-2 Inhibitor) OR Celecoxib OR Ketoprofen OR diclofenac OR flurbiprofen OR Ibuprofen OR Indomethacin OR ketorolac OR lornoxicam OR (mefenamic acid) OR meloxicam OR naproxen OR piroxicam OR sulindac OR celecoxib OR valdecoxib OR lumiracoxib OR rofecoxib OR etoricoxib OR parecoxib OR etodolac OR Analgesics OR Anodynes OR (Analgesic Drugs) OR Analgesic OR (Analgesic Agents) OR (Antinociceptive Agents) OR (Agent Antinociceptive) OR Dipyrone OR Methamizole OR Metamizol OR Dipyronium OR Metamizole OR Acetaminophen OR Acetominophen OR Acetaminophen OR Acetamidophenol OR (Analgesics Opioid) OR (Opioid Analgesics) OR (Opioid Analgesic) OR Opioids OR Opioid OR (Opioid Partial Agonists) OR (Agonists Opioid Partial) OR (Full Opioid Agonists) OR tramadol OR codeine OR pentazocine OR tapentadol OR oxycodone |
| **#3**  **Preemptive use** | Preemptive OR Preemptive OR (Pre emptive) OR Preventive OR Premedication OR (Preoperative Care) OR (Care Preoperative) OR (Preoperative Procedure) OR (Procedures Preoperative) OR (Preoperative Period) OR (Period Preoperative) OR (Acute pain) OR (Acute pains) OR (Post-surgical Pain) OR (Pain Post-surgical) OR (Post surgical Pain) OR (Pain Post-operative) OR (Pain Post operative) OR (Postsurgical Pain) OR (Post-operative Pain) OR (Post operative Pain) OR (Post-operative Pains) OR (Postoperative Pain) OR (Postoperative Pain Chronic) OR (Pain Chronic Postoperative) OR (Chronic Postoperative Pain) OR (Chronic Post-surgical Pain) OR (Chronic Post surgical Pain) OR (Pain Chronic Post-surgical) OR (Post-surgical Pain Chronic) OR (Chronic Postsurgical Pain) OR (Chronic Postsurgical Pains) OR (Pain Chronic Postsurgical) OR (Postsurgical Pain Chronic) OR (Persistent Postsurgical Pain) OR (Pain Persistent Postsurgical) OR (Postsurgical Pain Persistent) OR (Post-operative Pain Chronic) OR (Pain Chronic Post-operative) OR (Post operative Pain Chronic) OR (Chronic Post-operative Pain) OR (Chronic Post operative Pain) OR (Postoperative Pain Acute) OR (Pain Acute Postoperative) OR (Acute Postoperative Pain) OR (Acute Post-operative Pain) OR (Acute Post operative Pain) OR (Post-operative Pain Acute) OR (Pain Acute Post-operative) OR (Post operative Pain Acute) |
| **#4**  **Systematic review** | (((systematic review[ti] OR systematic literature review[ti] OR systematic scoping review[ti] OR systematic narrative review[ti] OR systematic qualitative review[ti] OR systematic evidence review[ti] OR systematic quantitative review[ti] OR systematic meta-review[ti] OR systematic critical review[ti] OR systematic mixed studies review[ti] OR systematic mapping review[ti] OR systematic cochrane review[ti] OR systematic search and review[ti] OR systematic integrative review[ti]) NOT comment[pt] NOT (protocol[ti] OR protocols[ti])) NOT MEDLINE [subset]) OR (Cochrane Database Syst Rev[ta] AND review[pt]) OR systematic review[pt] |
| **#5** | **#1 AND #2 AND #3 AND #4** |

**MEDLINE (PubMed) (n= 280 results)**

 ((((Pulpitis OR Pulpitides OR (Endodontic Inflammation) OR (Endodontic Inflammations) OR endodontics OR endodontology OR (Dental Pulp Diseases) OR (Dental Pulp Disease) OR (root canal therapy) OR (root canal therapies) OR endodontic OR (periapical periodontitis) OR (Periapical Periodontitides) OR (Apical Periodontitides) OR (Apical Periodontitis) OR (Acute Nonsuppurative Periodontitides) OR (Acute Nonsuppurative Periodontitis) OR (dental pulp necrosis) OR (Dental Pulp Necroses) OR (Dental Pulp Necrosis) OR (Pulp Necroses) OR (Pulp Necrosis) OR (dental pulp gangrene) OR (dental pulp gangrenes) OR (Pulp Gangrene) OR (Pulp Gangrenes) OR (Pulp Mummification) OR (Pulp Mummifications) OR (Dental Pulp Autolysis) OR (Dental Pulp Autolyses) OR (nonvital tooth) OR (tooth devitalized) OR (tooth pulpless) OR (teeth pulpless) OR (teeth devitalized) OR (nonvital teeth) OR (teeth endodontically-treated) OR (teeth endodontically treated) OR (tooth endodontically-treated) OR (tooth endodontically treated) OR (granuloma periapical) OR (granulomas periapical) OR (periapical periodontitis chronic nonsuppurative) OR (periodontitis apical chronic nonsuppurative) OR (dental granulomas) OR (dental granuloma) OR (periodontitis apical suppurative) OR (abscess periapical) OR (abscesses periapical) OR (periapical periodontitides suppurative) OR (alveolar abscess apical) OR (abscesses apical alveolar) OR (dentoalveolar abscess apical) OR (abscesses apical dentoalveolar) OR Pulpectomy OR Pulpectomies OR (root canal preparation) OR (root canal preparations)) OR ((Maxillofacial Surgery) OR (Oral Surgery) OR Exodontics OR (Third Molar) OR (Third Molars) OR (Wisdom Tooth) OR (Wisdom Teeth) OR (Impacted Tooth) OR (Impacted Teeth) OR (Dental Implant) OR (Dental Implants) OR (Unerupted Tooth) OR (Unerupted Teeth) OR (Supernumerary Tooth) OR (Supernumerary Teeth) OR (Fourth Molar) OR (Fourth Molars) OR (Bone Transplantation) OR (Bone Grafting) OR Autograft OR (Autologous Transplants) OR (Autologous Transplant) OR Autotransplants OR Autotransplant OR Allografts OR Allograft OR (Allogeneic Transplants) OR (Allogeneic Transplant) OR (Allogeneic Grafts) OR (Allogeneic Graft) OR Homografts OR Homograft OR (Homologous Transplants) OR (Homologous Transplant) OR Heterografts OR Heterograft OR Xenografts OR Xenograft)) AND ((Adrenal Cortex Hormones) OR Corticosteroids OR Corticosteroid OR Corticoids OR Corticoid OR (Glucocorticoids) OR Glucocorticoid OR (Glucocorticoid Effect) OR (Glucorticoid Effects) OR (Anti-Inflammatory Agents) OR (Anti Inflammatory Agents) OR (Antiinflammatory Agent) OR (Antiinflammatory Agents) OR Anti-Inflammatories OR (Anti Inflammatories) OR (Antiinflammatories) OR (Anti-Inflammatory Agent) OR (Anti Inflammatory Agent) OR (Agents Anti-Inflammatory) OR (Agents Anti Inflammatory) OR Dexamethasone OR Methylfluorprednisolone OR Betamethasone OR Flubenisolone OR Betadexamethasone OR (Methylprednisolone Acetate) OR Methylprednisolone OR Methylprednisolone-21-acetate OR (Methylprednisolone 21 acetate) OR Acetyl-Methylprednisolone OR (Acetyl Methylprednisolone) OR 6-Methylprednisolone OR (6 Methylprednisolone) OR Prednisone OR Dehydrocortisone OR delta-Cortisone OR Prednisolone OR Hidrocortisone OR cortisol OR (Anti-Inflammatory Agents Non-Steroidal) OR NSAID OR (Nonsteroidal Anti-Inflammatory Agent) OR (Agent Nonsteroidal Anti-Inflammatory) OR (Anti-Inflammatory Agent Nonsteroidal) OR (Nonsteroidal Anti Inflammatory Agent) OR NSAIDS OR (Antiinflammatory Agents Non Steroidal) OR (Antiinflammatory Agents Nonsteroidal) OR (Nonsteroidal Antiinflammatory Agents) OR (Non-Steroidal Anti-Inflammatory Agents) OR (Non Steroidal Anti Inflammatory Agents) OR (Nonsteroidal Anti-Inflammatory Agents) OR (Nonsteroidal Anti Inflammatory Agents) OR (Non-Steroidal Anti-Inflammatory Agent) OR (Agent Non-Steroidal Anti-Inflammatory) OR (Anti-Inflammatory Agent Non-Steroidal) OR (Non Steroidal Anti Inflammatory Agent) OR (Anti Inflammatory Agents Nonsteroidal) OR (Analgesics Anti-Inflammatory) OR (Anti-Inflammatory Analgesics) OR (Aspirin-Like Agents) OR (Aspirin Like Agents) OR (Aspirin-Like Agent) OR (Agent Aspirin-Like) OR (Aspirin Like Agent) OR (Cyclooxygenase 2 Inhibitors) OR (Cyclooxygenase 2 Inhibitor) OR (COX-2 Inhibitor) OR (COX 2 Inhibitor) OR Coxib OR Coxibs OR (COX-2 Inhibitors) OR (COX 2 Inhibitors) OR (COX2 Inhibitors) OR (COX2 Inhibitor) OR (Cyclooxygenase-2 Inhibitors) OR (Cyclooxygenase-2 Inhibitor) OR Celecoxib OR Ketoprofen OR diclofenac OR flurbiprofen OR Ibuprofen OR Indomethacin OR ketorolac OR lornoxicam OR (mefenamic acid) OR meloxicam OR naproxen OR piroxicam OR sulindac OR celecoxib OR valdecoxib OR lumiracoxib OR rofecoxib OR etoricoxib OR parecoxib OR etodolac OR Analgesics OR Anodynes OR (Analgesic Drugs) OR Analgesic OR (Analgesic Agents) OR (Antinociceptive Agents) OR (Agent Antinociceptive) OR Dipyrone OR Methamizole OR Metamizol OR Dipyronium OR Metamizole OR Acetaminophen OR Acetominophen OR Acetaminophen OR Acetamidophenol OR (Analgesics Opioid) OR (Opioid Analgesics) OR (Opioid Analgesic) OR Opioids OR Opioid OR (Opioid Partial Agonists) OR (Agonists Opioid Partial) OR (Full Opioid Agonists) OR tramadol OR codeine OR pentazocine OR tapentadol OR oxycodone)) AND (Preemptive OR Preemptive OR (Pre emptive) OR Preventive OR Premedication OR (Preoperative Care) OR (Care Preoperative) OR (Preoperative Procedure) OR (Procedures Preoperative) OR (Preoperative Period) OR (Period Preoperative) OR (Acute pain) OR (Acute pains) OR (Post-surgical Pain) OR (Pain Post-surgical) OR (Post surgical Pain) OR (Pain Post-operative) OR (Pain Post operative) OR (Postsurgical Pain) OR (Post-operative Pain) OR (Post operative Pain) OR (Post-operative Pains) OR (Postoperative Pain) OR (Postoperative Pain Chronic) OR (Pain Chronic Postoperative) OR (Chronic Postoperative Pain) OR (Chronic Post-surgical Pain) OR (Chronic Post surgical Pain) OR (Pain Chronic Post-surgical) OR (Post-surgical Pain Chronic) OR (Chronic Postsurgical Pain) OR (Chronic Postsurgical Pains) OR (Pain Chronic Postsurgical) OR (Postsurgical Pain Chronic) OR (Persistent Postsurgical Pain) OR (Pain Persistent Postsurgical) OR (Postsurgical Pain Persistent) OR (Post-operative Pain Chronic) OR (Pain Chronic Post-operative) OR (Post operative Pain Chronic) OR (Chronic Post-operative Pain) OR (Chronic Post operative Pain) OR (Postoperative Pain Acute) OR (Pain Acute Postoperative) OR (Acute Postoperative Pain) OR (Acute Post-operative Pain) OR (Acute Post operative Pain) OR (Post-operative Pain Acute) OR (Pain Acute Post-operative) OR (Post operative Pain Acute))) AND ((((systematic review[ti] OR systematic literature review[ti] OR systematic scoping review[ti] OR systematic narrative review[ti] OR systematic qualitative review[ti] OR systematic evidence review[ti] OR systematic quantitative review[ti] OR systematic meta-review[ti] OR systematic critical review[ti] OR systematic mixed studies review[ti] OR systematic mapping review[ti] OR systematic cochrane review[ti] OR systematic search and review[ti] OR systematic integrative review[ti]) NOT comment[pt] NOT (protocol[ti] OR protocols[ti])) NOT MEDLINE [subset]) OR (Cochrane Database Syst Rev[ta] AND review[pt]) OR systematic review[pt]) Sort by: Most Recent

**EMBASE (n= 191 results)**

Query('pulpitis'/exp OR pulpitis OR pulpitides OR (endodontic AND ('inflammation'/exp OR inflammation)) OR (endodontic AND inflammations) OR 'endodontics'/exp OR endodontics OR endodontology OR (('dental'/exp OR dental) AND ('pulp'/exp OR pulp) AND ('diseases'/exp OR diseases)) OR (('dental'/exp OR dental) AND ('pulp'/exp OR pulp) AND ('disease'/exp OR disease)) OR (('root'/exp OR root) AND canal AND ('therapy'/exp OR therapy)) OR (('root'/exp OR root) AND canal AND therapies) OR endodontic OR (periapical AND ('periodontitis'/exp OR periodontitis)) OR (periapical AND periodontitides) OR (apical AND periodontitides) OR (apical AND ('periodontitis'/exp OR periodontitis)) OR (acute AND nonsuppurative AND periodontitides) OR (acute AND nonsuppurative AND ('periodontitis'/exp OR periodontitis)) OR (('dental'/exp OR dental) AND ('pulp'/exp OR pulp) AND necroses) OR (('dental'/exp OR dental) AND ('pulp'/exp OR pulp) AND ('necrosis'/exp OR necrosis)) OR (('pulp'/exp OR pulp) AND necroses) OR (('pulp'/exp OR pulp) AND ('necrosis'/exp OR necrosis)) OR (('dental'/exp OR dental) AND ('pulp'/exp OR pulp) AND ('gangrene'/exp OR gangrene)) OR (('dental'/exp OR dental) AND ('pulp'/exp OR pulp) AND gangrenes) OR (('pulp'/exp OR pulp) AND ('gangrene'/exp OR gangrene)) OR (('pulp'/exp OR pulp) AND gangrenes) OR (('pulp'/exp OR pulp) AND ('mummification'/exp OR mummification)) OR (('pulp'/exp OR pulp) AND mummifications) OR (('dental'/exp OR dental) AND ('pulp'/exp OR pulp) AND ('autolysis'/exp OR autolysis)) OR (('dental'/exp OR dental) AND ('pulp'/exp OR pulp) AND autolyses) OR (nonvital AND ('tooth'/exp OR tooth)) OR (('tooth'/exp OR tooth) AND devitalized) OR (('tooth'/exp OR tooth) AND pulpless) OR (('teeth'/exp OR teeth) AND pulpless) OR (('teeth'/exp OR teeth) AND devitalized) OR (nonvital AND ('teeth'/exp OR teeth)) OR (('teeth'/exp OR teeth) AND 'endodontically treated') OR (('teeth'/exp OR teeth) AND endodontically AND treated) OR (('tooth'/exp OR tooth) AND 'endodontically treated') OR (('tooth'/exp OR tooth) AND endodontically AND treated) OR (('granuloma'/exp OR granuloma) AND periapical) OR (('granulomas'/exp OR granulomas) AND periapical) OR (periapical AND ('periodontitis'/exp OR periodontitis) AND chronic AND nonsuppurative) OR (('periodontitis'/exp OR periodontitis) AND apical AND chronic AND nonsuppurative) OR (('dental'/exp OR dental) AND ('granulomas'/exp OR granulomas)) OR (('dental'/exp OR dental) AND ('granuloma'/exp OR granuloma)) OR (('periodontitis'/exp OR periodontitis) AND apical AND suppurative) OR (('abscess'/exp OR abscess) AND periapical) OR (abscesses AND periapical) OR (periapical AND periodontitides AND suppurative) OR (alveolar AND ('abscess'/exp OR abscess) AND apical) OR (abscesses AND apical AND alveolar) OR (dentoalveolar AND ('abscess'/exp OR abscess) AND apical) OR (abscesses AND apical AND dentoalveolar) OR 'pulpectomy'/exp OR pulpectomy OR pulpectomies OR (('root'/exp OR root) AND canal AND ('preparation'/exp OR preparation)) OR (('root'/exp OR root) AND canal AND preparations) OR (maxillofacial AND surgery) OR (oral AND surgery) OR exodontics OR (third AND molar) OR (third AND molars) OR (wisdom AND tooth) OR (wisdom AND teeth) OR (impacted AND tooth) OR (impacted AND teeth) OR (dental AND implant) OR (dental AND implants) OR (unerupted AND tooth) OR (unerupted AND teeth) OR (supernumerary AND tooth) OR (supernumerary AND teeth) OR (fourth AND molar) OR (fourth AND molars) OR (bone AND transplantation) OR (bone AND grafting) OR autograft OR (autologous AND transplants) OR (autologous AND transplant) OR autotransplants OR autotransplant OR allografts OR allograft OR (allogeneic AND transplants) OR (allogeneic AND transplant) OR (allogeneic AND grafts) OR (allogeneic AND graft) OR homografts OR homograft OR (homologous AND transplants) OR (homologous AND transplant) OR heterografts OR heterograft OR xenografts OR xenograft) AND (adrenal AND cortex AND hormones OR corticosteroids OR corticosteroid OR corticoids OR corticoid OR glucocorticoids OR glucocorticoid OR (glucocorticoid AND effect) OR (glucorticoid AND effects) OR ('anti inflammatory' AND agents) OR (anti AND inflammatory AND agents) OR (antiinflammatory AND agent) OR (antiinflammatory AND agents) OR 'anti inflammatories' OR (anti AND inflammatories) OR antiinflammatories OR ('anti inflammatory' AND agent) OR (anti AND inflammatory AND agent) OR (agents AND 'anti inflammatory') OR (agents AND anti AND inflammatory) OR dexamethasone OR methylfluorprednisolone OR betamethasone OR flubenisolone OR betadexamethasone OR (methylprednisolone AND acetate) OR methylprednisolone OR 'methylprednisolone 21 acetate' OR (methylprednisolone AND 21 AND acetate) OR 'acetyl methylprednisolone' OR (acetyl AND methylprednisolone) OR '6 methylprednisolone' OR (6 AND methylprednisolone) OR prednisone OR dehydrocortisone OR 'delta cortisone' OR prednisolone OR hidrocortisone OR cortisol OR ('anti inflammatory' AND agents AND 'non steroidal') OR nsaid OR (nonsteroidal AND 'anti inflammatory' AND agent) OR (agent AND nonsteroidal AND 'anti inflammatory') OR ('anti inflammatory' AND agent AND nonsteroidal) OR (nonsteroidal AND anti AND inflammatory AND agent) OR NSAIDS OR (antiinflammatory AND agents AND non AND steroidal) OR (antiinflammatory AND agents AND nonsteroidal) OR (nonsteroidal AND antiinflammatory AND agents) OR ('non steroidal' AND 'anti inflammatory' AND agents) OR (non AND steroidal AND anti AND inflammatory AND agents) OR (nonsteroidal AND 'anti inflammatory' AND agents) OR (nonsteroidal AND anti AND inflammatory AND agents) OR ('non steroidal' AND 'anti inflammatory' AND agent) OR (agent AND 'non steroidal' AND 'anti inflammatory') OR ('anti inflammatory' AND agent AND 'non steroidal') OR (non AND steroidal AND anti AND inflammatory AND agent) OR (anti AND inflammatory AND agents AND nonsteroidal) OR (analgesics AND 'anti inflammatory') OR ('anti inflammatory' AND analgesics) OR ('aspirin like' AND agents) OR (aspirin AND like AND agents) OR ('aspirin like' AND agent) OR (agent AND 'aspirin like') OR (aspirin AND like AND agent) OR (cyclooxygenase AND 2 AND inhibitors) OR (cyclooxygenase AND 2 AND inhibitor) OR ('cox 2' AND inhibitor) OR (cox AND 2 AND inhibitor) OR coxib OR coxibs OR ('cox 2' AND inhibitors) OR (cox AND 2 AND inhibitors) OR (cox2 AND inhibitors) OR (cox2 AND inhibitor) OR ('cyclooxygenase 2' AND inhibitors) OR ('cyclooxygenase 2' AND inhibitor) OR ketoprofen OR diclofenac OR flurbiprofen OR ibuprofen OR indomethacin OR ketorolac OR lornoxicam OR (mefenamic AND acid) OR meloxicam OR naproxen OR piroxicam OR sulindac OR celecoxib OR valdecoxib OR lumiracoxib OR rofecoxib OR etoricoxib OR parecoxib OR etodolac OR analgesics OR anodynes OR (analgesic AND drugs) OR analgesic OR (analgesic AND agents) OR (antinociceptive AND agents) OR (agent AND antinociceptive) OR dipyrone OR methamizole OR metamizol OR dipyronium OR metamizole OR acetaminophen OR acetominophen OR acetaminophen OR acetamidophenol OR (analgesics AND opioid) OR (opioid AND analgesics) OR (opioid AND analgesic) OR opioids OR opioid OR (opioid AND partial AND agonists) OR (agonists AND opioid AND partial) OR (full AND opioid AND agonists) OR tramadol OR codeine OR pentazocine OR tapentadol OR oxycodone) AND (preemptive OR 'pre emptive' OR (pre AND emptive) OR preventive OR premedication OR (preoperative AND care) OR (care AND preoperative) OR (preoperative AND procedure) OR (procedures AND preoperative) OR (preoperative AND period) OR (period AND preoperative) OR (acute AND pain) OR (acute AND pains) OR ('post surgical' AND pain) OR (pain AND 'post surgical') OR (post AND surgical AND pain) OR (pain AND 'post operative') OR (pain AND post AND operative) OR (postsurgical AND pain) OR ('post operative' AND pain) OR (post AND operative AND pain) OR ('post operative' AND pains) OR (postoperative AND pain) OR (postoperative AND pain AND chronic) OR (pain AND chronic AND postoperative) OR (chronic AND postoperative AND pain) OR (chronic AND 'post surgical' AND pain) OR (chronic AND post AND surgical AND pain) OR (pain AND chronic AND 'post surgical') OR ('post surgical' AND pain AND chronic) OR (chronic AND postsurgical AND pain) OR (chronic AND postsurgical AND pains) OR (pain AND chronic AND postsurgical) OR (postsurgical AND pain AND chronic) OR (persistent AND postsurgical AND pain) OR (pain AND persistent AND postsurgical) OR (postsurgical AND pain AND persistent) OR ('post operative' AND pain AND chronic) OR (pain AND chronic AND 'post operative') OR (post AND operative AND pain AND chronic) OR (chronic AND 'post operative' AND pain) OR (chronic AND post AND operative AND pain) OR (postoperative AND pain AND acute) OR (pain AND acute AND postoperative) OR (acute AND postoperative AND pain) OR (acute AND 'post operative' AND pain) OR (acute AND post AND operative AND pain) OR ('post operative' AND pain AND acute) OR (pain AND acute AND 'post operative') OR (post AND operative AND pain AND acute)) AND ((('systematic review':ti OR 'systematic literature review':ti OR 'systematic scoping review':ti OR 'systematic narrative review':ti OR 'systematic qualitative review':ti OR 'systematic evidence review':ti OR 'systematic quantitative review':ti OR 'systematic meta-review':ti OR 'systematic critical review':ti OR 'systematic mixed studies review':ti OR 'systematic mapping review':ti OR 'systematic cochrane review':ti OR 'systematic search') AND review:ti OR 'systematic integrative review':ti) NOT comment:it NOT (protocol:ti OR protocols:ti) NOT 'medline [subset]' OR ('cochrane database syst rev[ta]' AND review:it) OR 'systematic review':it) AND [embase]/lim

**COCHRANE (n= 63 results)**

#1= Pulpitis OR Pulpitides OR (Endodontic Inflammation) OR (Endodontic Inflammations) OR endodontics OR endodontology OR (Dental Pulp Diseases) OR (Dental Pulp Disease) OR (root canal therapy) OR (root canal therapies) OR endodontic OR (periapical periodontitis) OR (Periapical Periodontitides) OR (Apical Periodontitides) OR (Apical Periodontitis) OR (Acute Nonsuppurative Periodontitides) OR (Acute Nonsuppurative Periodontitis) OR (dental pulp necrosis) OR (Dental Pulp Necroses) OR (Dental Pulp Necrosis) OR (Pulp Necroses) OR (Pulp Necrosis) OR (dental pulp gangrene) OR (dental pulp gangrenes) OR (Pulp Gangrene) OR (Pulp Gangrenes) OR (Pulp Mummification) OR (Pulp Mummifications) OR (Dental Pulp Autolysis) OR (Dental Pulp Autolyses) OR (nonvital tooth) OR (tooth devitalized) OR (tooth pulpless) OR (teeth pulpless) OR (teeth devitalized) OR (nonvital teeth) OR (teeth endodontically-treated) OR (teeth endodontically treated) OR (tooth endodontically-treated) OR (tooth endodontically treated) OR (granuloma periapical) OR (granulomas periapical) OR (periapical periodontitis chronic nonsuppurative) OR (periodontitis apical chronic nonsuppurative) OR (dental granulomas) OR (dental granuloma) OR (periodontitis apical suppurative) OR (abscess periapical) OR (abscesses periapical) OR (periapical periodontitides suppurative) OR (alveolar abscess apical) OR (abscesses apical alveolar) OR (dentoalveolar abscess apical) OR (abscesses apical dentoalveolar) OR Pulpectomy OR Pulpectomies OR (root canal preparation) OR (root canal preparations)

#2= (Maxillofacial Surgery) OR (Oral Surgery) OR Exodontics OR (Third Molar) OR (Third Molars) OR (Wisdom Tooth) OR (Wisdom Teeth) OR (Impacted Tooth) OR (Impacted Teeth) OR (Dental Implant) OR (Dental Implants) OR (Unerupted Tooth) OR (Unerupted Teeth) OR (Supernumerary Tooth) OR (Supernumerary Teeth) OR (Fourth Molar) OR (Fourth Molars) OR (Bone Transplantation) OR (Bone Grafting) OR Autograft OR (Autologous Transplants) OR (Autologous Transplant) OR Autotransplants OR Autotransplant OR Allografts OR Allograft OR (Allogeneic Transplants) OR (Allogeneic Transplant) OR (Allogeneic Grafts) OR (Allogeneic Graft) OR Homografts OR Homograft OR (Homologous Transplants) OR (Homologous Transplant) OR Heterografts OR Heterograft OR Xenografts OR Xenograft

#3= (Adrenal Cortex Hormones) OR Corticosteroids OR Corticosteroid OR Corticoids OR Corticoid OR (Glucocorticoids) OR Glucocorticoid OR (Glucocorticoid Effect) OR (Glucorticoid Effects) OR (Anti-Inflammatory Agents) OR (Anti Inflammatory Agents) OR (Antiinflammatory Agent) OR (Antiinflammatory Agents) OR Anti-Inflammatories OR (Anti Inflammatories) OR (Antiinflammatories) OR (Anti-Inflammatory Agent) OR (Anti Inflammatory Agent) OR (Agents Anti-Inflammatory) OR (Agents Anti Inflammatory) OR Dexamethasone OR Methylfluorprednisolone OR Betamethasone OR Flubenisolone OR Betadexamethasone OR (Methylprednisolone Acetate) OR Methylprednisolone OR Acetyl-Methylprednisolone OR (Acetyl Methylprednisolone) OR Prednisone OR Dehydrocortisone OR delta-Cortisone OR Prednisolone OR Hidrocortisone OR cortisol OR (Anti-Inflammatory Agents Non-Steroidal) OR NSAID OR (Nonsteroidal Anti-Inflammatory Agent) OR (Agent Nonsteroidal Anti-Inflammatory) OR (Anti-Inflammatory Agent Nonsteroidal) OR (Nonsteroidal Anti Inflammatory Agent) OR NSAIDS OR (Antiinflammatory Agents Non Steroidal) OR (Antiinflammatory Agents Nonsteroidal) OR (Nonsteroidal Antiinflammatory Agents) OR (Non-Steroidal Anti-Inflammatory Agents) OR (Non Steroidal Anti Inflammatory Agents) OR (Nonsteroidal Anti-Inflammatory Agents) OR (Nonsteroidal Anti Inflammatory Agents) OR (Non-Steroidal Anti-Inflammatory Agent) OR (Agent Non-Steroidal Anti-Inflammatory) OR (Anti-Inflammatory Agent Non-Steroidal) OR (Non Steroidal Anti Inflammatory Agent) OR (Anti Inflammatory Agents Nonsteroidal) OR (Analgesics Anti-Inflammatory) OR (Anti-Inflammatory Analgesics) OR (Aspirin-Like Agents) OR (Aspirin Like Agents) OR (Aspirin-Like Agent) OR (Agent Aspirin-Like) OR (Aspirin Like Agent) OR (Cyclooxygenase 2 Inhibitors) OR (Cyclooxygenase 2 Inhibitor) OR (COX-2 Inhibitor) OR (COX 2 Inhibitor) OR Coxib OR Coxibs OR (COX-2 Inhibitors) OR (COX 2 Inhibitors) OR (COX2 Inhibitors) OR (COX2 Inhibitor) OR (Cyclooxygenase-2 Inhibitors) OR (Cyclooxygenase-2 Inhibitor) OR Celecoxib OR Ketoprofen OR diclofenac OR flurbiprofen OR Ibuprofen OR Indomethacin OR ketorolac OR lornoxicam OR (mefenamic acid) OR meloxicam OR naproxen OR piroxicam OR sulindac OR celecoxib OR valdecoxib OR lumiracoxib OR rofecoxib OR etoricoxib OR parecoxib OR etodolac OR Analgesics OR Anodynes OR (Analgesic Drugs) OR Analgesic OR (Analgesic Agents) OR (Antinociceptive Agents) OR (Agent Antinociceptive) OR Dipyrone OR Methamizole OR Metamizol OR Dipyronium OR Metamizole OR Acetaminophen OR Acetominophen OR Acetaminophen OR Acetamidophenol OR (Analgesics Opioid) OR (Opioid Analgesics) OR (Opioid Analgesic) OR Opioids OR Opioid OR (Opioid Partial Agonists) OR (Agonists Opioid Partial) OR (Full Opioid Agonists) OR tramadol OR codeine OR pentazocine OR tapentadol OR oxycodone

#4= Preemptive OR Preemptive OR (Pre emptive) OR Preventive OR Premedication OR (Preoperative Care) OR (Care Preoperative) OR (Preoperative Procedure) OR (Procedures Preoperative) OR (Preoperative Period) OR (Period Preoperative) OR (Acute pain) OR (Acute pains) OR (Post-surgical Pain) OR (Pain Post-surgical) OR (Post surgical Pain) OR (Pain Post-operative) OR (Pain Post operative) OR (Postsurgical Pain) OR (Post-operative Pain) OR (Post operative Pain) OR (Post-operative Pains) OR (Postoperative Pain) OR (Postoperative Pain Chronic) OR (Pain Chronic Postoperative) OR (Chronic Postoperative Pain) OR (Chronic Post-surgical Pain) OR (Chronic Post surgical Pain) OR (Pain Chronic Post-surgical) OR (Post-surgical Pain Chronic) OR (Chronic Postsurgical Pain) OR (Chronic Postsurgical Pains) OR (Pain Chronic Postsurgical) OR (Postsurgical Pain Chronic) OR (Persistent Postsurgical Pain) OR (Pain Persistent Postsurgical) OR (Postsurgical Pain Persistent) OR (Post-operative Pain Chronic) OR (Pain Chronic Post-operative) OR (Post operative Pain Chronic) OR (Chronic Post-operative Pain) OR (Chronic Post operative Pain) OR (Postoperative Pain Acute) OR (Pain Acute Postoperative) OR (Acute Postoperative Pain) OR (Acute Post-operative Pain) OR (Acute Post operative Pain) OR (Post-operative Pain Acute) OR (Pain Acute Post-operative) OR (Post operative Pain Acute)

#5= #1 OR #2

#6= #5 AND #4 AND #3 (n=501)

Only systematic reviews (n=63)

**WEB OF SCIENCE (n= 155 results)**

#7= #6 AND #5 AND #4 AND #1 ([155](http://apps-webofknowledge.ez257.periodicos.capes.gov.br/summary.do?product=WOS&doc=1&qid=11&SID=7EGQ9RjqAZRCyhQcV4G&search_mode=CombineSearches&update_back2search_link_param=yes))

#6= #3 OR #2 ([453.911](http://apps-webofknowledge.ez257.periodicos.capes.gov.br/summary.do?product=WOS&doc=1&qid=10&SID=7EGQ9RjqAZRCyhQcV4G&search_mode=CombineSearches&update_back2search_link_param=yes))

#5= TS=(Preemptive OR Preemptive OR (Pre emptive) OR Preventive OR Premedication OR (Preoperative Care) OR (Care Preoperative) OR (Preoperative Procedure) OR (Procedures Preoperative) OR (Preoperative Period) OR (Period Preoperative) OR (Acute pain) OR (Acute pains) OR (Post-surgical Pain) OR (Pain Post-surgical) OR (Post surgical Pain) OR (Pain Post-operative) OR (Pain Post operative) OR (Postsurgical Pain) OR (Post-operative Pain) OR (Post operative Pain) OR (Post-operative Pains) OR (Postoperative Pain) OR (Postoperative Pain Chronic) OR (Pain Chronic Postoperative) OR (Chronic Postoperative Pain) OR (Chronic Post-surgical Pain) OR (Chronic Post surgical Pain) OR (Pain Chronic Post-surgical) OR (Post-surgical Pain Chronic) OR (Chronic Postsurgical Pain) OR (Chronic Postsurgical Pains) OR (Pain Chronic Postsurgical) OR (Postsurgical Pain Chronic) OR (Persistent Postsurgical Pain) OR (Pain Persistent Postsurgical) OR (Postsurgical Pain Persistent) OR (Post-operative Pain Chronic) OR (Pain Chronic Post-operative) OR (Post operative Pain Chronic) OR (Chronic Post-operative Pain) OR (Chronic Post operative Pain) OR (Postoperative Pain Acute) OR (Pain Acute Postoperative) OR (Acute Postoperative Pain) OR (Acute Post-operative Pain) OR (Acute Post operative Pain) OR (Post-operative Pain Acute) OR (Pain Acute Post-operative) OR (Post operative Pain Acute)) ([381.553](http://apps-webofknowledge.ez257.periodicos.capes.gov.br/summary.do?product=WOS&doc=1&qid=9&SID=7EGQ9RjqAZRCyhQcV4G&search_mode=AdvancedSearch&update_back2search_link_param=yes))

# 4= TS=((Adrenal Cortex Hormones) OR Corticosteroids OR Corticosteroid OR Corticoids OR Corticoid OR (Glucocorticoids) OR Glucocorticoid OR (Glucocorticoid Effect) OR (Glucorticoid Effects) OR (Anti-Inflammatory Agents) OR (Anti Inflammatory Agents) OR (Antiinflammatory Agent) OR (Antiinflammatory Agents) OR Anti-Inflammatories OR (Anti Inflammatories) OR (Antiinflammatories) OR (Anti-Inflammatory Agent) OR (Anti Inflammatory Agent) OR (Agents Anti-Inflammatory) OR (Agents Anti Inflammatory) OR Dexamethasone OR Methylfluorprednisolone OR Betamethasone OR Flubenisolone OR Betadexamethasone OR (Methylprednisolone Acetate) OR Methylprednisolone OR Methylprednisolone-21-acetate OR (Methylprednisolone 21 acetate) OR Acetyl-Methylprednisolone OR (Acetyl Methylprednisolone) OR 6-Methylprednisolone OR (6 Methylprednisolone) OR Prednisone OR Dehydrocortisone OR delta-Cortisone OR Prednisolone OR Hidrocortisone OR cortisol OR (Anti-Inflammatory Agents Non-Steroidal) OR NSAID OR (Nonsteroidal Anti-Inflammatory Agent) OR (Agent Nonsteroidal Anti-Inflammatory) OR (Anti-Inflammatory Agent Nonsteroidal) OR (Nonsteroidal Anti Inflammatory Agent) OR NSAIDS OR (Antiinflammatory Agents Non Steroidal) OR (Antiinflammatory Agents Nonsteroidal) OR (Nonsteroidal Antiinflammatory Agents) OR (Non-Steroidal Anti-Inflammatory Agents) OR (Non Steroidal Anti Inflammatory Agents) OR (Nonsteroidal Anti-Inflammatory Agents) OR (Nonsteroidal Anti Inflammatory Agents) OR (Non-Steroidal Anti-Inflammatory Agent) OR (Agent Non-Steroidal Anti-Inflammatory) OR (Anti-Inflammatory Agent Non-Steroidal) OR (Non Steroidal Anti Inflammatory Agent) OR (Anti Inflammatory Agents Nonsteroidal) OR (Analgesics Anti-Inflammatory) OR (Anti-Inflammatory Analgesics) OR (Aspirin-Like Agents) OR (Aspirin Like Agents) OR (Aspirin-Like Agent) OR (Agent Aspirin-Like) OR (Aspirin Like Agent) OR (Cyclooxygenase 2 Inhibitors) OR (Cyclooxygenase 2 Inhibitor) OR (COX-2 Inhibitor) OR (COX 2 Inhibitor) OR Coxib OR Coxibs OR (COX-2 Inhibitors) OR (COX 2 Inhibitors) OR (COX2 Inhibitors) OR (COX2 Inhibitor) OR (Cyclooxygenase-2 Inhibitors) OR (Cyclooxygenase-2 Inhibitor) OR Celecoxib OR Ketoprofen OR diclofenac OR flurbiprofen OR Ibuprofen OR Indomethacin OR ketorolac OR lornoxicam OR (mefenamic acid) OR meloxicam OR naproxen OR piroxicam OR sulindac OR celecoxib OR valdecoxib OR lumiracoxib OR rofecoxib OR etoricoxib OR parecoxib OR etodolac OR Analgesics OR Anodynes OR (Analgesic Drugs) OR Analgesic OR (Analgesic Agents) OR (Antinociceptive Agents) OR (Agent Antinociceptive) OR Dipyrone OR Methamizole OR Metamizol OR Dipyronium OR Metamizole OR Acetaminophen OR Acetominophen OR Acetaminophen OR Acetamidophenol OR (Analgesics Opioid) OR (Opioid Analgesics) OR (Opioid Analgesic) OR Opioids OR Opioid OR (Opioid Partial Agonists) OR (Agonists Opioid Partial) OR (Full Opioid Agonists) OR tramadol OR codeine OR pentazocine OR tapentadol OR oxycodone) ([847.533](http://apps-webofknowledge.ez257.periodicos.capes.gov.br/summary.do?product=WOS&doc=1&qid=8&SID=7EGQ9RjqAZRCyhQcV4G&search_mode=AdvancedSearch&update_back2search_link_param=yes))

# 3= TS=((Maxillofacial Surgery) OR (Oral Surgery) OR Exodontics OR (Third Molar) OR (Third Molars) OR (Wisdom Tooth) OR (Wisdom Teeth) OR (Impacted Tooth) OR (Impacted Teeth) OR (Dental Implant) OR (Dental Implants) OR (Unerupted Tooth) OR (Unerupted Teeth) OR (Supernumerary Tooth) OR (Supernumerary Teeth) OR (Fourth Molar) OR (Fourth Molars) OR (Bone Transplantation) OR (Bone Grafting) OR Autograft OR (Autologous Transplants) OR (Autologous Transplant) OR Autotransplants OR Autotransplant OR Allografts OR Allograft OR (Allogeneic Transplants) OR (Allogeneic Transplant) OR (Allogeneic Grafts) OR (Allogeneic Graft) OR Homografts OR Homograft OR (Homologous Transplants) OR (Homologous Transplant) OR Heterografts OR Heterograft OR Xenografts OR Xenograft) ([431.640](http://apps-webofknowledge.ez257.periodicos.capes.gov.br/summary.do?product=WOS&doc=1&qid=7&SID=7EGQ9RjqAZRCyhQcV4G&search_mode=AdvancedSearch&update_back2search_link_param=yes))

# 2= TS=(Pulpitis OR Pulpitides OR (Endodontic Inflammation) OR (Endodontic Inflammations) OR endodontics OR endodontology OR (Dental Pulp Diseases) OR (Dental Pulp Disease) OR (root canal therapy) OR (root canal therapies) OR endodontic OR (periapical periodontitis) OR (Periapical Periodontitides) OR (Apical Periodontitides) OR (Apical Periodontitis) OR (Acute Nonsuppurative Periodontitides) OR (Acute Nonsuppurative Periodontitis) OR (dental pulp necrosis) OR (Dental Pulp Necroses) OR (Dental Pulp Necrosis) OR (Pulp Necroses) OR (Pulp Necrosis) OR (dental pulp gangrene) OR (dental pulp gangrenes) OR (Pulp Gangrene) OR (Pulp Gangrenes) OR (Pulp Mummification) OR (Pulp Mummifications) OR (Dental Pulp Autolysis) OR (Dental Pulp Autolyses) OR (nonvital tooth) OR (tooth devitalized) OR (tooth pulpless) OR (teeth pulpless) OR (teeth devitalized) OR (nonvital teeth) OR (teeth endodontically-treated) OR (teeth endodontically treated) OR (tooth endodontically-treated) OR (tooth endodontically treated) OR (granuloma periapical) OR (granulomas periapical) OR (periapical periodontitis chronic nonsuppurative) OR (periodontitis apical chronic nonsuppurative) OR (dental granulomas) OR (dental granuloma) OR (periodontitis apical suppurative) OR (abscess periapical) OR (abscesses periapical) OR (periapical periodontitides suppurative) OR (alveolar abscess apical) OR (abscesses apical alveolar) OR (dentoalveolar abscess apical) OR (abscesses apical dentoalveolar) OR Pulpectomy OR Pulpectomies OR (root canal preparation) OR (root canal preparations)) ([24.998](http://apps-webofknowledge.ez257.periodicos.capes.gov.br/summary.do?product=WOS&doc=1&qid=5&SID=7EGQ9RjqAZRCyhQcV4G&search_mode=AdvancedSearch&update_back2search_link_param=yes))

# 1 TS=("systematic review" OR "systematic literature review" OR "systematic scoping review" OR "systematic narrative review" OR "systematic qualitative review" OR "systematic evidence review" OR "systematic quantitative review" OR "systematic meta-review" OR "systematic critical review" OR "systematic mixed studies review" OR "systematic mapping review" OR "systematic cochrane review" OR "systematic search") ([231.082](http://apps-webofknowledge.ez257.periodicos.capes.gov.br/summary.do?product=WOS&doc=1&qid=4&SID=7EGQ9RjqAZRCyhQcV4G&search_mode=AdvancedSearch&update_back2search_link_param=yes))

**Scopus (n= 284 results)**

(Filtros de RS apenas em TITLE-ABS-KEY) excluindo indexados no medline e embase

( ( ( ALL ( *pulpitis*  OR  *pulpitides*  OR  ( *endodontic*  AND *inflammation* )  OR  ( *endodontic*  AND *inflammations* )  OR  *endodontics*  OR  *endodontology*  OR  ( *dental*  AND *pulp*  AND *diseases* )  OR  ( *dental*  AND *pulp*  AND *disease* )  OR  ( *root*  AND *canal*  AND *therapy* )  OR  ( *root*  AND *canal*  AND *therapies* )  OR  *endodontic*  OR  ( *periapical*  AND *periodontitis* )  OR  ( *periapical*  AND *periodontitides* )  OR  ( *apical*  AND *periodontitides* )  OR  ( *apical*  AND *periodontitis* )  OR  ( *acute*  AND *nonsuppurative*  AND *periodontitides* )  OR  ( *acute*  AND *nonsuppurative*  AND *periodontitis* )  OR  ( *dental*  AND *pulp*  AND *necrosis* )  OR  ( *dental*  AND *pulp*  AND *necroses* )  OR  ( *dental*  AND *pulp*  AND *necrosis* )  OR  ( *pulp*  AND *necroses* )  OR  ( *pulp*  AND *necrosis* )  OR  ( *dental*  AND *pulp*  AND *gangrene* )  OR  ( *dental*  AND *pulp*  AND *gangrenes* )  OR  ( *pulp*  AND *gangrene* )  OR  ( *pulp*  AND *gangrenes* )  OR  ( *pulp*  AND *mummification* )  OR  ( *pulp*  AND *mummifications* )  OR  ( *dental*  AND *pulp*  AND *autolysis* )  OR  ( *dental*  AND *pulp*  AND *autolyses* )  OR  ( *nonvital*  AND *tooth* )  OR  ( *tooth*  AND *devitalized* )  OR  ( *tooth*  AND *pulpless* )  OR  ( *teeth*  AND *pulpless* )  OR  ( *teeth*  AND *devitalized* )  OR  ( *nonvital*  AND *teeth* )  OR  ( *teeth*  AND *endodontically-treated* )  OR  ( *teeth*  AND *endodontically*  AND *treated* )  OR  ( *tooth*  AND *endodontically-treated* )  OR  ( *tooth*  AND *endodontically*  AND *treated* )  OR  ( *granuloma*  AND *periapical* )  OR  ( *granulomas*  AND *periapical* )  OR  ( *periapical*  AND *periodontitis*  AND *chronic*  AND *nonsuppurative* )  OR  ( *periodontitis*  AND *apical*  AND *chronic*  AND *nonsuppurative* )  OR  ( *dental*  AND *granulomas* )  OR  ( *dental*  AND *granuloma* )  OR  ( *periodontitis*  AND *apical*  AND *suppurative* )  OR  ( *abscess*  AND *periapical* )  OR  ( *abscesses*  AND *periapical* )  OR  ( *periapical*  AND *periodontitides*  AND *suppurative* )  OR  ( *alveolar*  AND *abscess*  AND *apical* )  OR  ( *abscesses*  AND *apical*  AND *alveolar* )  OR  ( *dentoalveolar*  AND *abscess*  AND *apical* )  OR  ( *abscesses*  AND *apical*  AND *dentoalveolar* )  OR  *pulpectomy*  OR  *pulpectomies*  OR  ( *root*  AND *canal*  AND *preparation* )  OR  ( *root*  AND *canal*  AND *preparations* ) ) )  OR  ( ALL ( ( *maxillofacial*  AND *surgery* )  OR  ( *oral*  AND *surgery* )  OR  *exodontics*  OR  ( *third*  AND *molar* )  OR  ( *third*  AND *molars* )  OR  ( *wisdom*  AND *tooth* )  OR  ( *wisdom*  AND *teeth* )  OR  ( *impacted*  AND *tooth* )  OR  ( *impacted*  AND *teeth* )  OR  ( *dental*  AND *implant* )  OR  ( *dental*  AND *implants* )  OR  ( *unerupted*  AND *tooth* )  OR  ( *unerupted*  AND *teeth* )  OR  ( *supernumerary*  AND *tooth* )  OR  ( *supernumerary*  AND *teeth* )  OR  ( *fourth*  AND *molar* )  OR  ( *fourth*  AND *molars* )  OR  ( *bone*  AND *transplantation* )  OR  ( *bone*  AND *grafting* )  OR  *autograft*  OR  ( *autologous*  AND *transplants* )  OR  ( *autologous*  AND *transplant* )  OR  *autotransplants*  OR  *autotransplant*  OR  *allografts*  OR  *allograft*  OR  ( *allogeneic*  AND *transplants* )  OR  ( *allogeneic*  AND *transplant* )  OR  ( *allogeneic*  AND *grafts* )  OR  ( *allogeneic*  AND *graft* )  OR  *homografts*  OR  *homograft*  OR  ( *homologous*  AND *transplants* )  OR  ( *homologous*  AND *transplant* )  OR  *heterografts*  OR  *heterograft*  OR  *xenografts*  OR  *xenograft* ) ) )  AND  ( ALL ( ( *adrenal*  AND *cortex*  AND *hormones* )  OR  *corticosteroids*  OR  *corticosteroid*  OR  *corticoids*  OR  *corticoid*  OR  ( *glucocorticoids* )  OR  *glucocorticoid*  OR  ( *glucocorticoid*  AND *effect* )  OR  ( *glucorticoid*  AND *effects* )  OR  ( *anti-inflammatory*  AND *agents* )  OR  ( *anti*  AND *inflammatory*  AND *agents* )  OR  ( *antiinflammatory*  AND *agent* )  OR  ( *antiinflammatory*  AND *agents* )  OR  *anti-inflammatories*  OR  ( *anti*  AND *inflammatories* )  OR  ( *antiinflammatories* )  OR  ( *anti-inflammatory*  AND *agent* )  OR  ( *anti*  AND *inflammatory*  AND *agent* )  OR  ( *agents*  AND *anti-inflammatory* )  OR  ( *agents*  AND *anti*  AND *inflammatory* )  OR  *dexamethasone*  OR  *methylfluorprednisolone*  OR  *betamethasone*  OR  *flubenisolone*  OR  *betadexamethasone*  OR  ( *methylprednisolone*  AND *acetate* )  OR  *methylprednisolone*  OR  *methylprednisolone-21-acetate*  OR  ( *methylprednisolone*  *21*  *acetate* )  OR  *acetyl-methylprednisolone*  OR  ( *acetyl*  AND *methylprednisolone* )  OR  *6-methylprednisolone*  OR  ( *6*  *methylprednisolone* )  OR  *prednisone*  OR  *dehydrocortisone*  OR  *delta-cortisone*  OR  *prednisolone*  OR  *hidrocortisone*  OR  *cortisol*  OR  ( *anti-inflammatory*  AND *agents*  AND *non-steroidal* )  OR  *nsaid*  OR  ( *nonsteroidal*  AND *anti-inflammatory*  AND *agent* )  OR  ( *agent*  AND *nonsteroidal*  AND *anti-inflammatory* )  OR  ( *anti-inflammatory*  AND *agent*  AND *nonsteroidal* )  OR  ( *nonsteroidal*  AND *anti*  AND *inflammatory*  AND *agent* )  OR  *NSAIDS*  OR  ( *antiinflammatory*  AND *agents*  AND *non*  AND *steroidal* )  OR  ( *antiinflammatory*  AND *agents*  AND *nonsteroidal* )  OR  ( *nonsteroidal*  AND *antiinflammatory*  AND *agents* )  OR  ( *non-steroidal*  AND *anti-inflammatory*  AND *agents* )  OR  ( *non*  AND *steroidal*  AND *anti*  AND *inflammatory*  AND *agents* )  OR  ( *nonsteroidal*  AND *anti-inflammatory*  AND *agents* )  OR  ( *nonsteroidal*  AND *anti*  AND *inflammatory*  AND *agents* )  OR  ( *non-steroidal*  AND *anti-inflammatory*  AND *agent* )  OR  ( *agent*  AND *non-steroidal*  AND *anti-inflammatory* )  OR  ( *anti-inflammatory*  AND *agent*  AND *non-steroidal* )  OR  ( *non*  AND *steroidal*  AND *anti*  AND *inflammatory*  AND *agent* )  OR  ( *anti*  AND *inflammatory*  AND *agents*  AND *nonsteroidal* )  OR  ( *analgesics*  AND *anti-inflammatory* )  OR  ( *anti-inflammatory*  AND *analgesics* )  OR  ( *aspirin-like*  AND *agents* )  OR  ( *aspirin*  AND *like*  AND *agents* )  OR  ( *aspirin-like*  AND *agent* )  OR  ( *agent*  AND *aspirin-like* )  OR  ( *aspirin*  AND *like*  AND *agent* )  OR  ( *cyclooxygenase*  *2*  *inhibitors* )  OR  ( *cyclooxygenase*  *2*  *inhibitor* )  OR  ( *cox-2*  AND *inhibitor* )  OR  ( *cox*  *2*  *inhibitor* )  OR  *coxib*  OR  *coxibs*  OR  ( *cox-2*  AND *inhibitors* )  OR  ( *cox*  *2*  *inhibitors* )  OR  ( *cox2*  AND *inhibitors* )  OR  ( *cox2*  AND *inhibitor* )  OR  ( *cyclooxygenase-2*  AND *inhibitors* )  OR  ( *cyclooxygenase-2*  AND *inhibitor* )  OR  *celecoxib*  OR  *ketoprofen*  OR  *diclofenac*  OR  *flurbiprofen*  OR  *ibuprofen*  OR  *indomethacin*  OR  *ketorolac*  OR  *lornoxicam*  OR  ( *mefenamic*  AND *acid* )  OR  *meloxicam*  OR  *naproxen*  OR  *piroxicam*  OR  *sulindac*  OR  *celecoxib*  OR  *valdecoxib*  OR  *lumiracoxib*  OR  *rofecoxib*  OR  *etoricoxib*  OR  *parecoxib*  OR  *etodolac*  OR  *analgesics*  OR  *anodynes*  OR  ( *analgesic*  AND *drugs* )  OR  *analgesic*  OR  ( *analgesic*  AND *agents* )  OR  ( *antinociceptive*  AND *agents* )  OR  ( *agent*  AND *antinociceptive* )  OR  *dipyrone*  OR  *methamizole*  OR  *metamizol*  OR  *dipyronium*  OR  *metamizole*  OR  *acetaminophen*  OR  *acetominophen*  OR  *acetaminophen*  OR  *acetamidophenol*  OR  ( *analgesics*  AND *opioid* )  OR  ( *opioid*  AND *analgesics* )  OR  ( *opioid*  AND *analgesic* )  OR  *opioids*  OR  *opioid*  OR  ( *opioid*  AND *partial*  AND *agonists* )  OR  ( *agonists*  AND *opioid*  AND *partial* )  OR  ( *full*  AND *opioid*  AND *agonists* )  OR  *tramadol*  OR  *codeine*  OR  *pentazocine*  OR  *tapentadol*  OR  *oxycodone* ) )  AND  ( ALL ( *preemptive*  OR  *preemptive*  OR  ( *pre*  AND *emptive* )  OR  *preventive*  OR  *premedication*  OR  ( *preoperative*  AND *care* )  OR  ( *care*  AND *preoperative* )  OR  ( *preoperative*  AND *procedure* )  OR  ( *procedures*  AND *preoperative* )  OR  ( *preoperative*  AND *period* )  OR  ( *period*  AND *preoperative* )  OR  ( *acute*  AND *pain* )  OR  ( *acute*  AND *pains* )  OR  ( *post-surgical*  AND *pain* )  OR  ( *pain*  AND *post-surgical* )  OR  ( *post*  AND *surgical*  AND *pain* )  OR  ( *pain*  AND *post-operative* )  OR  ( *pain*  AND *post*  AND *operative* )  OR  ( *postsurgical*  AND *pain* )  OR  ( *post-operative*  AND *pain* )  OR  ( *post*  AND *operative*  AND *pain* )  OR  ( *post-operative*  AND *pains* )  OR  ( *postoperative*  AND *pain* )  OR  ( *postoperative*  AND *pain*  AND *chronic* )  OR  ( *pain*  AND *chronic*  AND *postoperative* )  OR  ( *chronic*  AND *postoperative*  AND *pain* )  OR  ( *chronic*  AND *post-surgical*  AND *pain* )  OR  ( *chronic*  AND *post*  AND *surgical*  AND *pain* )  OR  ( *pain*  AND *chronic*  AND *post-surgical* )  OR  ( *post-surgical*  AND *pain*  AND *chronic* )  OR  ( *chronic*  AND *postsurgical*  AND *pain* )  OR  ( *chronic*  AND *postsurgical*  AND *pains* )  OR  ( *pain*  AND *chronic*  AND *postsurgical* )  OR  ( *postsurgical*  AND *pain*  AND *chronic* )  OR  ( *persistent*  AND *postsurgical*  AND *pain* )  OR  ( *pain*  AND *persistent*  AND *postsurgical* )  OR  ( *postsurgical*  AND *pain*  AND *persistent* )  OR  ( *post-operative*  AND *pain*  AND *chronic* )  OR  ( *pain*  AND *chronic*  AND *post-operative* )  OR  ( *post*  AND *operative*  AND *pain*  AND *chronic* )  OR  ( *chronic*  AND *post-operative*  AND *pain* )  OR  ( *chronic*  AND *post*  AND *operative*  AND *pain* )  OR  ( *postoperative*  AND *pain*  AND *acute* )  OR  ( *pain*  AND *acute*  AND *postoperative* )  OR  ( *acute*  AND *postoperative*  AND *pain* )  OR  ( *acute*  AND *post-operative*  AND *pain* )  OR  ( *acute*  AND *post*  AND *operative*  AND *pain* )  OR  ( *post-operative*  AND *pain*  AND *acute* )  OR  ( *pain*  AND *acute*  AND *post-operative* )  OR  ( *post*  AND *operative*  AND *pain*  AND *acute* ) ) )  AND  ( TITLE-ABS-KEY ( ( *systematic*  AND *review* )  OR  ( *systematic*  AND *literature*  AND *review* )  OR  ( *systematic*  AND *scoping*  AND *review* )  OR  ( *systematic*  AND *narrative*  AND *review* )  OR  ( *systematic*  AND *qualitative*  AND *review* )  OR  ( *systematic*  AND *evidence*  AND *review* )  OR  ( *systematic*  AND *quantitative*  AND *review* )  OR  ( *systematic*  AND *meta-review* )  OR  ( *systematic*  AND *critical*  AND *review* )  OR  ( *systematic*  AND *mixed*  AND *studies*  AND *review* )  OR  ( *systematic*  AND *mapping*  AND *review* )  OR  ( *systematic*  AND *cochrane*  AND *review* )  OR  ( *systematic*  AND *search*  AND  *review* )  OR  ( *systematic*  AND *integrative*  AND *review* ) ) ) )  AND NOT  ( INDEX ( *medline* )  OR  INDEX ( *embase* ) )

**Virtual Health Library (n= 408 results)**
(pulpitis OR pulpitides OR (endodontic inflammation) OR (endodontic inflammations) OR endodontics OR endodontology OR (dental pulp diseases) OR (dental pulp disease) OR (root canal therapy) OR (root canal therapies) OR endodontic OR (periapical periodontitis) OR (periapical periodontitides) OR (apical periodontitides) OR (apical periodontitis) OR (acute nonsuppurative periodontitides) OR (acute nonsuppurative periodontitis) OR (dental pulp necrosis) OR (dental pulp necroses) OR (dental pulp necrosis) OR (pulp necroses) OR (pulp necrosis) OR (dental pulp gangrene) OR (dental pulp gangrenes) OR (pulp gangrene) OR (pulp gangrenes) OR (pulp mummification) OR (pulp mummifications) OR (dental pulp autolysis) OR (dental pulp autolyses) OR (nonvital tooth) OR (tooth devitalized) OR (tooth pulpless) OR (teeth pulpless) OR (teeth devitalized) OR (nonvital teeth) OR (teeth endodontically-treated) OR (teeth endodontically treated) OR (tooth endodontically-treated) OR (tooth endodontically treated) OR (granuloma periapical) OR (granulomas periapical) OR (periapical periodontitis chronic nonsuppurative) OR (periodontitis apical chronic nonsuppurative) OR (dental granulomas) OR (dental granuloma) OR (periodontitis apical suppurative) OR (abscess periapical) OR (abscesses periapical) OR (periapical periodontitides suppurative) OR (alveolar abscess apical) OR (abscesses apical alveolar) OR (dentoalveolar abscess apical) OR (abscesses apical dentoalveolar) OR pulpectomy OR pulpectomies OR (root canal preparation) OR (root canal preparations) OR (maxillofacial surgery) OR (oral surgery) OR exodontics OR (third molar) OR (third molars) OR (wisdom tooth) OR (wisdom teeth) OR (impacted tooth) OR (impacted teeth) OR (dental implant) OR (dental implants) OR (unerupted tooth) OR (unerupted teeth) OR (supernumerary tooth) OR (supernumerary teeth) OR (fourth molar) OR (fourth molars) OR (bone transplantation) OR (bone grafting) OR autograft OR (autologous transplants) OR (autologous transplant) OR autotransplants OR autotransplant OR allografts OR allograft OR (allogeneic transplants) OR (allogeneic transplant) OR (allogeneic grafts) OR (allogeneic graft) OR homografts OR homograft OR (homologous transplants) OR (homologous transplant) OR heterografts OR heterograft OR xenografts OR xenograft) AND ((adrenal cortex hormones) OR corticosteroids OR corticosteroid OR corticoids OR corticoid OR (glucocorticoids) OR glucocorticoid OR (glucocorticoid effect) OR (glucorticoid effects) OR (anti-inflammatory agents) OR (anti inflammatory agents) OR (antiinflammatory agent) OR (antiinflammatory agents) OR anti-inflammatories OR (anti inflammatories) OR (antiinflammatories) OR (anti-inflammatory agent) OR (anti inflammatory agent) OR (agents anti-inflammatory) OR (agents anti inflammatory) OR dexamethasone OR methylfluorprednisolone OR betamethasone OR flubenisolone OR betadexamethasone OR (methylprednisolone acetate) OR methylprednisolone OR methylprednisolone-21-acetate OR (methylprednisolone 21 acetate) OR acetyl-methylprednisolone OR (acetyl methylprednisolone) OR 6-methylprednisolone OR (6 methylprednisolone) OR prednisone OR dehydrocortisone OR delta-cortisone OR prednisolone OR hidrocortisone OR cortisol OR (anti-inflammatory agents non-steroidal) OR nsaid OR (nonsteroidal anti-inflammatory agent) OR (agent nonsteroidal anti-inflammatory) OR (anti-inflammatory agent nonsteroidal) OR (nonsteroidal anti inflammatory agent) OR NSAIDS OR (antiinflammatory agents non steroidal) OR (antiinflammatory agents nonsteroidal) OR (nonsteroidal antiinflammatory agents) OR (non-steroidal anti-inflammatory agents) OR (non steroidal anti inflammatory agents) OR (nonsteroidal anti-inflammatory agents) OR (nonsteroidal anti inflammatory agents) OR (non-steroidal anti-inflammatory agent) OR (agent non-steroidal anti-inflammatory) OR (anti-inflammatory agent non-steroidal) OR (non steroidal anti inflammatory agent) OR (anti inflammatory agents nonsteroidal) OR (analgesics anti-inflammatory) OR (anti-inflammatory analgesics) OR (aspirin-like agents) OR (aspirin like agents) OR (aspirin-like agent) OR (agent aspirin-like) OR (aspirin like agent) OR (cyclooxygenase 2 inhibitors) OR (cyclooxygenase 2 inhibitor) OR (cox-2 inhibitor) OR (cox 2 inhibitor) OR coxib OR coxibs OR (cox-2 inhibitors) OR (cox 2 inhibitors) OR (cox2 inhibitors) OR (cox2 inhibitor) OR (cyclooxygenase-2 inhibitors) OR (cyclooxygenase-2 inhibitor) OR celecoxib OR ketoprofen OR diclofenac OR flurbiprofen OR ibuprofen OR indomethacin OR ketorolac OR lornoxicam OR (mefenamic acid) OR meloxicam OR naproxen OR piroxicam OR sulindac OR celecoxib OR valdecoxib OR lumiracoxib OR rofecoxib OR etoricoxib OR parecoxib OR etodolac OR analgesics OR anodynes OR (analgesic drugs) OR analgesic OR (analgesic agents) OR (antinociceptive agents) OR (agent antinociceptive) OR dipyrone OR methamizole OR metamizol OR dipyronium OR metamizole OR acetaminophen OR acetominophen OR acetaminophen OR acetamidophenol OR (analgesics opioid) OR (opioid analgesics) OR (opioid analgesic) OR opioids OR opioid OR (opioid partial agonists) OR (agonists opioid partial) OR (full opioid agonists) OR tramadol OR codeine OR pentazocine OR tapentadol OR oxycodone) AND (preemptive OR preemptive OR (pre emptive) OR preventive OR premedication OR (preoperative care) OR (care preoperative) OR (preoperative procedure) OR (procedures preoperative) OR (preoperative period) OR (period preoperative) OR (acute pain) OR (acute pains) OR (post-surgical pain) OR (pain post-surgical) OR (post surgical pain) OR (pain post-operative) OR (pain post operative) OR (postsurgical pain) OR (post-operative pain) OR (post operative pain) OR (post-operative pains) OR (postoperative pain) OR (postoperative pain chronic) OR (pain chronic postoperative) OR (chronic postoperative pain) OR (chronic post-surgical pain) OR (chronic post surgical pain) OR (pain chronic post-surgical) OR (post-surgical pain chronic) OR (chronic postsurgical pain) OR (chronic postsurgical pains) OR (pain chronic postsurgical) OR (postsurgical pain chronic) OR (persistent postsurgical pain) OR (pain persistent postsurgical) OR (postsurgical pain persistent) OR (post-operative pain chronic) OR (pain chronic post-operative) OR (post operative pain chronic) OR (chronic post-operative pain) OR (chronic post operative pain) OR (postoperative pain acute) OR (pain acute postoperative) OR (acute postoperative pain) OR (acute post-operative pain) OR (acute post operative pain) OR (post-operative pain acute) OR (pain acute post-operative) OR (post operative pain acute)) AND ( type_of_study:("systematic_reviews"))

**Epistemonikos (n= 52 results)**

(title:(Pulpitis OR Pulpitides OR (Endodontic Inflammation) OR endodontics OR (Dental Pulp Disease) OR (root canal therapy) OR (root canal therapies) OR endodontic OR (periapical periodontitis) OR (Periapical Periodontitides) OR (Apical Periodontitides) OR (Apical Periodontitis) OR (Acute Nonsuppurative Periodontitides) OR (Acute Nonsuppurative Periodontitis) OR (Dental pulp necrosis) OR (Dental Pulp Necroses) OR (Pulp Necroses) OR (Pulp Necrosis) OR (Dental pulp gangrenes) OR (Pulp Gangrene) OR (Pulp Gangrenes) OR (granuloma periapical) OR (granulomas periapical) OR (periapical periodontitis chronic nonsuppurative) OR (periodontitis apical chronic nonsuppurative) OR (dental granulomas) OR (dental granuloma) OR (periodontitis apical suppurative) OR (abscess periapical) OR (abscesses periapical) OR (alveolar abscess apical) OR (abscesses apical alveolar) OR (dentoalveolar abscess apical) OR Pulpectomy OR Pulpectomies OR (root canal preparation) OR (Maxillofacial Surgery) OR (Oral Surgery) OR Exodontics OR (Third Molar) OR (Third Molars) OR (Wisdom Tooth) OR (Wisdom Teeth) OR (Impacted Tooth) OR (Impacted Teeth) OR (Dental Implant) OR (Dental Implants)) OR abstract:(Pulpitis OR Pulpitides OR (Endodontic Inflammation) OR endodontics OR (Dental Pulp Disease) OR (root canal therapy) OR (root canal therapies) OR endodontic OR (periapical periodontitis) OR (Periapical Periodontitides) OR (Apical Periodontitides) OR (Apical Periodontitis) OR (Acute Nonsuppurative Periodontitides) OR (Acute Nonsuppurative Periodontitis) OR (Dental pulp necrosis) OR (Dental Pulp Necroses) OR (Pulp Necroses) OR (Pulp Necrosis) OR (Dental pulp gangrenes) OR (Pulp Gangrene) OR (Pulp Gangrenes) OR (granuloma periapical) OR (granulomas periapical) OR (periapical periodontitis chronic nonsuppurative) OR (periodontitis apical chronic nonsuppurative) OR (dental granulomas) OR (dental granuloma) OR (periodontitis apical suppurative) OR (abscess periapical) OR (abscesses periapical) OR (alveolar abscess apical) OR (abscesses apical alveolar) OR (dentoalveolar abscess apical) OR Pulpectomy OR Pulpectomies OR (root canal preparation) OR (Maxillofacial Surgery) OR (Oral Surgery) OR Exodontics OR (Third Molar) OR (Third Molars) OR (Wisdom Tooth) OR (Wisdom Teeth) OR (Impacted Tooth) OR (Impacted Teeth) OR (Dental Implant) OR (Dental Implants))) AND (title:(Corticosteroids OR Corticosteroid OR Corticoids OR Corticoid OR Glucocorticoids OR Glucocorticoid OR (Anti-Inflammatory Agents) OR (Anti Inflammatory Agents) OR (Antiinflammatory Agent) OR (Antiinflammatory Agents) OR Anti-Inflammatories OR Antiinflammatories OR (Anti-Inflammatory Agents Non-Steroidal) OR NSAID OR (Nonsteroidal Anti-Inflammatory Agent) OR (Anti-Inflammatory Agent Nonsteroidal) OR (Nonsteroidal Anti Inflammatory Agent) OR NSAIDS OR (Antiinflammatory Agents Non Steroidal) OR (Antiinflammatory Agents Nonsteroidal) OR (Non-Steroidal Anti-Inflammatory Agents) OR (Nonsteroidal Anti Inflammatory Agents) OR (Non-Steroidal Anti-Inflammatory Agent) OR (Agent Non-Steroidal Anti-Inflammatory) OR (Anti-Inflammatory Agent Non-Steroidal) OR (Non Steroidal Anti Inflammatory Agent) OR (Anti Inflammatory Agents Nonsteroidal)) OR abstract:(Corticosteroids OR Corticosteroid OR Corticoids OR Corticoid OR Glucocorticoids OR Glucocorticoid OR (Anti-Inflammatory Agents) OR (Anti Inflammatory Agents) OR (Antiinflammatory Agent) OR (Antiinflammatory Agents) OR Anti-Inflammatories OR Antiinflammatories OR (Anti-Inflammatory Agents Non-Steroidal) OR NSAID OR (Nonsteroidal Anti-Inflammatory Agent) OR (Anti-Inflammatory Agent Nonsteroidal) OR (Nonsteroidal Anti Inflammatory Agent) OR NSAIDS OR (Antiinflammatory Agents Non Steroidal) OR (Antiinflammatory Agents Nonsteroidal) OR (Non-Steroidal Anti-Inflammatory Agents) OR (Nonsteroidal Anti Inflammatory Agents) OR (Non-Steroidal Anti-Inflammatory Agent) OR (Agent Non-Steroidal Anti-Inflammatory) OR (Anti-Inflammatory Agent Non-Steroidal) OR (Non Steroidal Anti Inflammatory Agent) OR (Anti Inflammatory Agents Nonsteroidal))) AND (title:(Preemptive OR Preemptive OR (Pre emptive) OR Preventive OR Premedication OR (Preoperative Care) OR (Period Preoperative) OR (Acute pain) OR (Post-surgical Pain) OR (Pain Post-operative) OR (Pain Post operative) OR (Postsurgical Pain) OR (Post-operative Pain) OR (Post operative Pain) OR (Postoperative Pain) OR (Acute Post-operative Pain) OR (Acute Post operative Pain)) OR abstract:(Preemptive OR Preemptive OR (Pre emptive) OR Preventive OR Premedication OR (Preoperative Care) OR (Period Preoperative) OR (Acute pain) OR (Post-surgical Pain) OR (Pain Post-operative) OR (Pain Post operative) OR (Postsurgical Pain) OR (Post-operative Pain) OR (Post operative Pain) OR (Postoperative Pain) OR (Acute Post-operative Pain) OR (Acute Post operative Pain)))

**B - List of excluded studies (n= 71)**

| **STUDIES** | **REASONS FOR EXCLUSION** |
| --- | --- |
| 1. ALEXANDER, L. *et al*. The combination of non-selective NSAID 400 mg and paracetamol 1000 mg is more effective than each drug alone for treatment of acute pain. A systematic review Swed Dent J, v. 38, n. 1, p. 1-14, 2014. | Other intervention |
| 1. ASHLEY, P. F. *et al*. Preoperative analgesics for additional pain relief in children and adolescents having dental treatment. Cochrane Database of Systematic Review, v. 2012, n. 9, 2012. | Other population |
| 1. ASHLEY, P. F. *et al*. Preoperative analgesics for additional pain relief in children and adolescents having dental treatment. Cochrane Database of Systematic Review, v. 2016, n. 8, 2016. | Other population |
| 1. AU, A. H. *et al*. The Efficacy and Clinical Safety of Various Analgesic Combinations for Post-Operative Pain after Third Molar Surgery: A Systematic Review and Meta-Analysis. PLoS One, v. 10, n. 6, 2015. | Other intervention |
| 1. BAILEY, E.; WORTHINGTON, H.; COULTHARD, P. Ibuprofen and/or paracetamol (acetaminophen) for pain relief after surgical removal of lower wisdom teeth, a Cochrane systematic review. Brazilian Dental Journal, v. 216, n. 8, p. 451-5, 2014. | Other intervention |
| 1. BAVANA SRI, S.; SARAH SATHIYAWATHIE, R.; GURUNATHAN, D. A review of perioperative corticosteroid use in oral and maxillofacial surgery. Drug Invention Today, v.12, n. 3, p.611-613, 2019. | Other type of study |
| 1. BHAVARAJU SA. et. al. Pre-emptive administration of gabapentinoids to reduce postoperative pain and opioid usage following oral and maxillofacial surgical procedures. Oral Surg. 00:1–10, 2021. https://doi.org/10.1111/ors.12617 | Other intervention |
| 1. BRIGNARDELLO-PETERSEN, R. Corticosteroids may reduce pain after endodontic treatment. Journal of the American Dental Association, v. 150, n. 3, E31-E31, 2019. | Other population |
| 1. CALATAYUD, C. *et al*. Anestesia en la endodoncia de molares y premolares mandibulares con pulpitis aguda irreversible. Recomendaciones clínicas. Hacia una revisión sistemática. Cient. dent. (Ed. impr.), v. 14, v. 2, p. 141-148, 2017. | Other population |
| 1. CLARKE, R. *et al*. Single dose oral etoricoxib for acute postoperative pain in adult. Cochrane Database of Systematic Review, n. 2, 2009. | Other intervention |
| 1. CLARKE, R. SHEENA DERRY; R ANDREW MOORE. Single dose oral etoricoxib for acute postoperative pain in adults. Cochrane Database of Systematic Reviews, v. 2014, n. 5, 2014. | Other intervention |
| 1. COLLINS, S. L. *et al*. Oral ibuprofen and diclofenac in post-operative pain: a quantitative systematic review. European Journal of Pain, v. 2, n. 4, p. 285-291, 1998. | Other intervention |
| 1. CORBELLA, S. *et al*. Inferior alveolar nerve block for the treatment of teeth presenting with irreversible pulpitis: A systematic review of the literature and meta-analysis. Quintessence Int, v. 48, n. 1, p. 69-82, 2017. | Other outcomes |
| 1. COSTA, F. W. G. *et al*. Does the Preemptive Use of Oral Nonsteroidal Anti-inflammatory Drugs Reduce Postoperative Pain in Surgical Removal of Third Molars? A Meta analysis of Randomized Clinical Trials. Anesth Prog, v. 62, p. 57-63, 2015. | Primary studies included in another systematic review |
| 1. CHEN, Q. Submucosal injection of dexamethasone reduces postoperative discomfort after third-molar extraction: A systematic review and meta-analysis. J Am Dent Assoc. 2017 v. 148, n. 2, p. 81-91, 2017. | Primary studies included in another systematic review |
| 1. DAN, A. E. B. *et al*. Corticosteroid administration in oral and orthognathic surgery: a systematic review of the literatura and meta-analysis. J Oral Maxillofac Surg, v. 68, p. 2207-2220, 2010. | Primary studies included in another systematic review |
| 1. DE GEUS, J. L. *et al*. Effect of ibuprofen on the efficacy of inferior alveolar nerve block in patients with irreversible pulpitis: A meta-analysis. Australian Endodontic Journal, v. 45, n. 2, p. 246-258, 2019. | Other population |
| 1. DEVJI, T. Nonnarcotic analgesics reduce average pain up to 24 hours after endodontic treatment compared with placebo. Journal of the American Dental Association, v. 149, n. 9:E128-E128, 2018. | Other population |
| 1. DOLEMAN, B, *et al*. Pre-emptive and preventive opioids for postoperative pain in adults undergoing all types of surgery. Cochrane Database of Systematic Reviews, Issue 12. Art. No.: CD0126242018,. | Primary studies included in another systematic review |
| 1. ESPARZA-VILLALPANDO, V. *et al*. Local Ketamine Improves Postoperative Analgesia After Third Molar Surgery. J Oral Maxillofac Surg, v. 77, n. 12, p. 2386-2400, 2019. | Other intervention |
| 1. FAN, D. *et al*. Pre-emptive use of non-steroids anti-inflammatory drugs for a successful inferior alveolar nerve block in patients with irreversible pulpitis: A systematic review and network meta-analysis. International Journal of Clinical and Experimental Medicine, v. 11, n.11, p. 11567-11577, 2018. | Other outcomes |
| 1. FERNANDES, I. A. *et al*. Intramuscular injection of dexamethasone for the control of pain, swelling, and trismus after third molar surgery: a systematic review and meta-analysis. Int J Oral Maxillofac Surg, v. 48, n. 5, p. 659-668, may 2019. | Other intervention |
| 1. FRANCO-DE LA TORRE L. *et al*. A Meta-Analysis of the Analgesic Efficacy of Single-Doses of Ibuprofen Compared to Traditional Non-Opioid Analgesics Following Third Molar Surgery. Pharmaceuticals 14, 360, 2021. https://doi.org/10.3390/ph14040360 | Other intervention |
| 1. GATELY F. The effect of pre-emptive ibuprofen on post-operative pain after removal of lower third molar teeth: a systematic review. Evid Based Dent. 2022 Mar 4. doi: 10.1038/s41432-021-0211-1. Epub ahead of print. Erratum in: Evid Based Dent. 2022 Jun;23(2):47. PMID: 35246613. | Primary studies included in another systematic review |
| 1. GONÇALVES K. K. N. *et al*. Is the injection of tramadol effective at control of pain after impacted mandibular third molar extractions? A systematic review and meta-analysis. Med Oral Patol Oral Cir Bucal. Nov 1;27 (6):e560-8, 2022. | Other intervention |
| 1. González-Morelos M. *et al*. Efficacy of Methylprednisolone Compared to Other Drugs for Pain, Swelling, and Trismus Control after Third Molar Surgery: A Meta-Analysis. Healthcare, v.10, 1028, 2022. https://doi.org/10.3390/healthcare10061028 | Primary studies included in another systematic review |
| 1. HALL, P. E. *et al*. Single dose oral lornoxicam for acute postoperative pain in adults. Cochrane Database of Systematic Review, v. 2009, n. 4, out 2009. | Other intervention |
| 1. HOU *et al*. Comparison of Submucosal With Intramuscular or Intravenous Administration of Dexamethasone for Third Molar Surgeries: A Systematic Review and Meta-Analysis. Front. Surg. 8:714950, 2021. doi: 10.3389/fsurg.2021.714950 | Other intervention |
| 1. ISIORDIA-ESPINOZA *et al*. Analgesic efficacy and safety of single-dose tramadol and non-steroidal anti-inflammatory drugs in operations on the third molars: a systematic review and meta-analysis. British Journal of Orl and Maxillofacial Surgery, v. 52, p. 775-783, 2014. | Primary studies included in another systematic review |
| 1. KANDAMANI, J. *et al*. Submucosal administration of dexamethasone versus methyl prednisolone in management of postoperative sequelae after mandibular third molar impaction. A systematic review. Int. J. Res. Pharm. Sci., v. 11, n. 3, p. 4479-4486, 2020. | Primary studies included in another systematic review |
| 1. KARAPINAR-KAZANDAG, M.; TANALP, J.; ERSEV, H. Effect of Premedication on the Success of Inferior Alveolar Nerve Block in Patients with Irreversible Pulpitis: A Systematic Review of the Literature. Biomed Res Int, 2019. | Other outcomes |
| 1. KHOULY I. *et al*. Post-operative pain management in dental implant surgery: a systematic review and meta-analysis of randomized clinical trials. Clinical Oral Investigations, v. 25, p. 2511-2536, 2021. | Primary studies included in another systematic review |
| 1. LAPIDUS, D. *et al*. Effect of premedication to provide analgesia as a supplement to inferior alveolar nerve block in patients with irreversible pulpitis. Journal of the American Dental Association, v. 147, n. 6, p. 427-37, 2016. | Other outcomes |
| 1. LARSEN M. K. *et al*. Different dosages of corticosteroid and routes of administration in mandibular third molar surgery: a systematic review. J Oral Maxillofac Res, v. 9, n. 2, apr-jun 2018. | Primary studies included in another systematic review |
| 1. LI, C. *et al*. Preoperative oral nonsteroidal anti-inflammatory drugs for the success of the inferior alveolar nerve block in irreversible pulpitis treatment: a systematic review and meta-analysis based on randomized controlled trials. Quintessence Int, v. 43, n. 3, p. 209-19, mar 2012. | Other outcomes |
| 1. MCQUAY H. J. Pre-emptive analgesia: a systematic review of clinical studies. Ann Med, v. 27, p. 249-256, 1995. | Other intervention |
| 1. MACARIO, A; LIPMAN A.G. Ketorolac in the era of cyclo-oxygenase-2 selective nonsteroidal anti-inflammatory drugs: a systematic review of efficacy, side effects, and regulatory issues. Pain Medicine, v. 2, n. 4, p. 336-351, 2001. | Other population |
| 1. MADHOON H. W. *et al*. The efficacy of methylprednisolone versus dexamethasone in reducing postoperative sequelae after third molar surgery: A systematic review and meta-analysis. Journal of Oral and Maxillofacial Surgery, Medicine, and Pathology. v. 34, p. 365–374, 2022. | Primary studies included in another systematic review |
| 1. MALLAMA *et al*. A systematic review and trial sequential analysis of intravenous vs. Oral peri-operative paracetamol. Anaesthesia. v. 76, p. 270-276, 2021. | Surgical procedure performed under general anesthesia |
| 1. MATTOS-PEREIRA *et al*. Preemptive analgesia in dental implant surgery: A systematic review and meta-analysis of randomized controlled trials. Med Oral Patol Oral Cir Bucal. v. 1, n. 26(5), p. 632-41, sep 2021. | Primary studies included in another systematic review |
| 1. MARTÍNEZ LEGÓRBURU, J. *et al*. Analgesic efficacy of cyclooxygenase-2 specific inhibitors in patients with postoperative dental pain: A systematic review and metanalysis. European Journal of Clinical Pharmacology, v. 66, n. S96, 2010 | Full text not found |
| 1. MEDEIROS-ALBUQUERQUE, A. F. *et al*. Preemptive analgesia-related gene and protein expression in third molar surgeries under non-steroidal anti-inflammatory drug protocols: A PROSPERO-registered systematic review of clinical studies. Med Oral Patol Oral Cir Bucal, v. 23, n. 6, p. e723-e732, nov 2018. | Other outcomes |
| 1. MEDVE, R.A.; WANG, J.; KARIM, R. Tramadol and acetaminophen tablets for dental pain. Anesth Prog, v. 48, n.3, p. 79-81, 2001. | Other type of study |
| 1. MIROSHNYCHENKO A. *et al*. Acute Postoperative Pain Due to Dental Extraction in the Adult Population: A Systematic Review and Network Meta-analysis. Journal of Dental Research, vol. 102(4), p.391– 401, 2023 | Other intervention |
| 1. MOLL, R. *et al*. Single dose oral mefenamic acid for acute postoperative pain in adults. Cochrane Database of Systematic Reviews, v. 2017, v. 3, CD007553, 2011. | Other intervention |
| 1. MOORE, A. *et al*. Paracetamol with and without codeine in acute pain: A quantitative systematic review. Pain Apr, v. 70, n. 2-3, p. 193-201, 1997. | Other intervention |
| 1. MOORE, P. A.; HERSH, E.V. *et al*. Combining ibuprofen and acetaminophen for acute pain management after third-molar extractions: Translating clinical research to dental practice. J. Am. Dent. Assoc., v. 144, n. 8, p. 898-908, 2013. | Other type of study |
| 1. MORDINI L. *et al*. The use of corticosteroids in the lateral sinus augmentation surgical procedure: A systematic review and meta-analysis. A systematic review and meta-analysis. Clin Implant Dent Relat Res. v. 24(6), p. 776‐791, 2022. doi:10.1111/cid.13126 | Other type of study |
| 1. NAGENDRABABU, V. *et al*. Effect of Nonsteroidal Anti-inflammatory Drug as an Oral Premedication on the Anesthetic Success of Inferior Alveolar Nerve Block in Treatment of Irreversible Pulpitis: A Systematic Review with Meta-analysis and Trial Sequential Analysis. J Endod Jun, v. 44, n. 6, p. 914-922, 2018. | Other population |
| 1. O`HARE, P. E. *et al*. Effect of submucosal dexamethasone injections in the prevention of postoperative pain, trismus, and oedema associated with mandibular third molar surgery: a systematic review and meta-analysis. Int. J. Oral Maxillofac Surg, v. 48. p. 1456-1469, 2019. | Primary studies included in another systematic review |
| 1. ONG C.K. S. *et al*. The efficacy of preemptive analgesia for acute postoperative pain management: a meta-analysis. Anesth Analg, v. 100, p. 757-773, 2005. | Primary studies included in another systematic review |
| 1. PATEL, N.; BAILEY, E.; COULTHARD, P. Opioids for pain after oral surgery. Oral Surg., v. 7, n. 4, p. 196-202, 2014. | Other type of study |
| 1. PENPRASE B. *et al*. The efficacy of preemptive analgesia for postoperative pain control: a systematic review of the literature. AORN Journal, v. 101, n. 1, jan 2015. | Primary studies included in another systematic review |
| 1. PULIKKOTIL, S. J. *et al*. Effect of oral premedication on the anaesthetic efficacy of inferior alveolar nerve block in patients with irreversible pulpitis - A systematic review and network meta-analysis of randomized controlled trials. Int Endod J, v. 51, n.9, p.989-1004, set 2018. | Other population |
| 1. REFAHEE, S. M. *et al*. pre-emptive low-dose ketamine with local anesthesia reduces postoperative morbidity after third molar surgery: a systematic review and meta-analysis. J Oral Res, v. 9, n. 4, nov 2020. | Primary studies included in another systematic review |
| 1. SHIRVANI, A. *et al*. Effect of preoperative oral analgesics on pulpal anesthesia in patients with irreversible pulpitis-a systematic review and meta-analysis. Clin Oral Investig, v. 21, n. 1, p.43-52, jan 2017. | Other outcomes |
| 1. SHIBL *et al*. Effectiveness of pre-operative oral corticosteroids in reducing pain, trismus and oedema following lower third molar extractions: a systematic review. British Dental Journal. 8, jul, 2021. https://doi.org/10.1038/s41415-021-3165-y | Primary studies included in another systematic review |
| 1. SIVARAMAKRISHNAN, G.; SRIDHARAN, K. Oral Ketorolac with Inferior Alveolar Nerve Block for Irreversible Pulpitis: A Systematic Review and Meta-analysis. Open Dent J, v.12, p.340-346, abr 2018. | Other outcomes |
| 1. SHOORANIZAD E, PARVIN M. Comparison of the Effects of Dexamethasone Administration on Postoperative Sequelae Before and After “Third Molar" Extraction Surgeries. Endocrine, Methabolic & Immune Disorders – Drug Targets, v. 20, p. 356-364, 2020. | Other type of study |
| 1. STAGG, K. Intravenous versus oral paracetamol for postoperative analgesia: A systematic review. J. Perioperat. Pract., v. 31, n. 10, p. 373-378, out 2021. | Clinical trial with surgical procedures performed under general anesthesia |
| 1. SMITH L. A. *et al*. Single-dose ketorolac and pethidine in acute postoperative pain: systematic review with meta-analysis. British Journal of Anaesthesia. v. 84, n. 1, p. 48-58, 2000. | Other intervention |
| 1. STRAUBE S. *et al*. Effect of preoperative cox-II-seletive NSAIDS (coxibs) on postoperative outcomes: a systematic review of randomized studies. Acta Anaesthesiol Scand, v. 49, p. 601-613, 2005. | Primary studies included in another systematic review |
| 1. SUGRAGAN, C.*et al*. Do corticosteroids reduce postoperative pain following third molar intervention? J Dent Anesth Pain Med, v. 20, n. 5, p. 281-291, out 2020. | Primary studies included in another systematic review |
| 1. TIRUNAGARI, S. K. *et al*. Single dose oral etodolac for acute postoperative pain in adults. Cochrane Database of Systematic Review, v. 2009, n.3, Cd007357, jul 2009. | Other intervention |
| 1. TUPYOTA, P. *et al*. Supplementary techniques for pain control during root canal treatment of lower posterior teeth with irreversible pulpitis: A systematic review and meta-analysis. Aust Endod J, v. 44, n.1, p.14-25, abril 2018. | Other population |
| 1. WEIL, K. *et al*. Paracetamol for pain relief aftar surgical removal of lower wisdom teeth. Cochrane Database Syst Rev 2007, (3):CD004487. | Other intervention |
| 1. WEYANT, R. Paracetamol (Acetaminophen) is Safe and Effective for Treatment of Postoperative Third Molar Extraction Pain. J. Evid-Based Dent. Pract., v. 9, n.4, p.211-212, 2009. | Other type of study |
| 1. ZANJIR, M. *et al*. Efficacy and Safety of Pulpal Anesthesia Strategies during Endodontic Treatment of Permanent Mandibular Molars with Symptomatic Irreversible Pulpitis: A Systematic Review and Network Meta-analysis. J Endod, v.45, n.12, p.1435-1464, dez 2019. | Other population |
| 1. ZANJIR, M.; AZARPAZHOOH, A. Limited evidence suggests premedication with corticosteroids may help relieve postoperative endodontic pain in patients with symptomatic pulpitis undergoing single-visit root canal treatment. Journal of Evidence-Based Dental Practice, v. 20, n.1, p. 3, mar 2020. | Other population |
| 1. ZEMMEL, M.H. The role of COX-2 inhibitors in the perioperative setting: Efficacy and safety - A systematic review. AANA Journal, v.74, n.1, p.49-60, 2006. | Other type of study |
| 1. ZHOU, X. Y.; NANAYAKKARA, S. Corticosteroids can be effective introducing postoperative endodontic pain. Journal of Evidence-Based Dental Practice, v. 20, n.3, jun 2020. | Other population |

**C - Description of randomized clinical trials of adults undergoing third molar surgery and in preemptive use of corticosteroids (n= 70)**

| **Clinical trials**  (Number of SR) | **Drugs or placebo**  (Number of participants) | **Outcomes**  (Follow-up) | **Effectiveness outcomes** | **Safety outcomes** |
| --- | --- | --- | --- | --- |
| **DEXAMETHASONE (oral route) (20)** | | | | |
| Agostinho *et al*., 2014  (n= 2) | Dexamethasone 4mg (n= 27)  Dexamethasone 12mg (n= 27) | Pain, trismus and swelling  (2 days) | No statistical difference was observed between the groups | Not reported |
| Alcantara *et al*., 2013  (n= 3) | Dexamethasone 8mg (n= 18)  Methylprednisolone 40mg (n= 18) | Pain, trismus and swelling  (1, 2, 3 and 7 days) | No statistical difference was observed between the groups for pain outcome. Dexamethasone 8mg was superior to methylprednisolone 40mg for reducing trismus and swelling (up to 2 days) | Not reported |
| Barbalho *et al*., 2017  (n=1) | Dexametasone 8mg + nimesulide 100 mg  (n=10)  Dexametasona 8mg + placebo (n=10) | Pain and rescue analgesics  (2, 4, 6, 8, 12 and 16 hours, and 1, 2 and 3 days)  Trismus and swelling  (1, 2, 3 and 7 days) | Dexamethasone 8mg + nimesulide 100mg were superior to other group for pain outcome. Swelling and trismus were similar in the two treatment groups.  The need for the ingestion of a greater number of analgesics occur in dexamethasone 8mg + placebo combination | No adverse effects were observed |
| Bauer *et al*., 2013  (n= 2) | Dexamethasone 8mg + ibuprofen 600mg (n= 24)  Ibuprofen 600mg + placebo (n= 23)  Placebo (n= 47) | Pain  (1, 2, 3 days) | Dexamethasone 8mg with ibuprofen 600mg were superior to other groups for pain outcome | No adverse effects were observed |
| Baxendale; Valter; Lavery, 1993  (n= 2) | Dexamethasone 8mg (n= 24)  Placebo (n=25) | Pain and swelling  (1 and 4 hours) | Dexamethasone 8mg was superior to placebo for pain and swelling outcomes | Not reported |
| Bhargava; Sreekumar; Deshpande, 2013  (n= 1) | Dexamethasone 4mg (n= 10)  Dexamethasone 4mg (SM: 1mL) (n= 10)  Dexamethasone 4mg (pterygomandibular) (n= 10)  Dexamethasone 4mg (IM: 1mL) (n= 10)  Dexamethasone 4mg (IV: 1mL) (n=10)  No steroid (n= 10) | Pain and swelling  (1, 3 and 7 days) | Dexamethasone (any dose) was superior to “no steroid” group for pain (up to 7 days) and swelling (up to 3 days) outcomes. No difference was observed in the effectiveness of drugs when used by the different routes | Not reported |
| BORTOLUZZI *et al*., 2013  Bortoluzzi *et al*,. 2013  (n= 1) | Dexamethasone 8mg (n= 14)  Placebo (n=12) | Pain, trismus and swelling  (1, 3 and 4 days) | No statistical difference was observed between the groups | Not reported |
| Chaudhary *et al*., 2015  (n=1) | Dexamethasone 8mg (oral) (n=100)  Dexamethasone 4mg (IV) (n=100) | Pain, trismus and swelling  (1, 3 and 4 days) | There is no significant difference between two doses of administration of the dosage of dexamethasone IV 4mg or oral 8mg | Not reported |
| Darawade *et al*., 2013  (n= 3) | Dexamethasone 8mg (n= 25)  Methylprednisolone 40mg (n= 25) | Pain, trismus and swelling  (1, 2, 3 and 7 days) | No statistical difference was observed between the groups for pain outcome. Dexamethasone 8mg was superior to methylprednisolone 40mg for reducing trismus (up to 2 days) and swelling (up to 7 days) | Not reported |
| De Sousa Santos *et al*., 2012  (n= 2) | Dexamethasone 4mg + tramadol 50mg  (n= 30)  Diclofenac 50mg + tramadol 50mg (n= 30) | Pain and swelling  (12 hours; and 1 and 2 days)  Trismus (2 and 7 days) | Dexamethasone 4mg + tramadol 50mg was superior to diclofenac 50mg + tramadol 50mg for reducing pain (up to 12 hours), trismus (up to 7 days) and promoting less swelling (up to 2 days) | No adverse effects were observed |
| Konuganti; Rangaraj; Elizabeth, 2015  (n= 1) | Dexamethasone 8mg (n= 20)  Etoricoxib120mg (n= 20)  Placebo (n= 20) | Pain  (1 and 8 hours; 3 days) | Both drugs were effective for pain prevention after open‑flap debridement surgical procedure for up to 3 days | No adverse effects were observed |
| Laureano Filho *et al*. 2008  (n= 6) | Dexamethasone 4mg (n= 30)  Dexamethasone 8mg (n= 30) | Pain, trismus and swelling  (2 days) | No statistical difference was observed be­tween the groups for pain outcome. Dexamethasone 8mg was superior to dexamethasone 4mg for reducing trismus and swelling | Not reported |
| Lisboa; Pilatti, 2013  (n=2) | Dexamethasone 8mg (n= 10)  Ibuprofen 600mg + arginine 555mg  (n= 10)  Etoricoxib 90mg (n= 9)  Placebo (n= 13) | Pain and analgesic consumption  (2, 4, 6, 8, 12, 24, 36 and 48 hours) | All anti-inflammatories were superior to placebo for pain outcome. The analgesic consumption was lower with the use of dexamethasone 8mg and etoricoxib 90mg | Not reported |
| Maciel *et al*., 2019  (n=1) | Dexametasona 8mg + Ibuprofeno 600mg (n= 30)  Dexametasona 8mg + Ibuprofeno 600mg + Arginina 600mg (n= 30) | None information was found in systematic review | None information was found in systematic review | None information was found in systematic review |
| Oliveira *et al*., 2021  (n=2) | Dexamethasone 4mg (n= 22)  Placebo (n= 22) | Pain, edema and trismus  (1, 3 and 7 days) | Dexamethasone 4mg proved effective for controlling pain, reducing swelling for up to 3 days postoperatively. There was no significant difference for trismus. | No adverse effects were observed |
| Pansard *et al*., 2020  (n=1) | Dexamethasone 8mg (n= 44)  Placebo (n= 49) | Pain, edema and trismus (1, 3 and 7 days) | No significant differences were found between the intervention and control for outcomes measured | Not reported |
| Santos *et al*., 2021  (n=2) | Dexamethasone (4mg) (n= 20)  Acetaminophen (1000mg) (n= 20)  Ibuprofen (600mg) (n= 20)  Ketoprofen (100mg) (n= 20)  Nimesulide (100mg) (n= 20)  Placebo (n= 20) | Pain, edema and rescue medication  (1, 6, 12 hours, 1, 2 and 3 days) | Ibuprofen 600mg and nimesulide 100mg showed higher overall effects on pain scores over time, with no differences between them. Acetaminophen 1000mg showed significantly lower overall effects in edema control. Lower quantity of rescue medication was observed for ibuprofen and nimesulide | No adverse effects were observed |
| Simoni *et al*., 203  (n= 4) | Dexamethasone 8mg (n= 20)  Diclofenac 50mg (n= 20)  Placebo (n= 14) | Pain and rescue medication  (1, 6, 8 and 12 hours; 2, 4 and 7 days) | Dexamethasone 8mg orally was superior to diclofenac 50mg and placebo for reducing pain outcome. Total amount of rescue medication did not differ among the groups | Not reported |
| Sotto-Maior; Senna; Assis, 2011  (n= 2) | Dexamethasone 4mg (n= 50)  Etoricoxib 120mg (n= 50) | Pain, trismus and swelling  (1 and 2 days) | No statistical difference was observed between the groups for outcomes evaluated | Not reported |
| Steffens *et al*., 2010  (n= 1) | Dexamethasone 8mg (n= 15)  Etoricoxib120mg (n= 15)  Placebo (n= 15) | Pain and rescue medication  (1 to 8 hours; 3 and 4 days) | Dexamethasone 8mg and etoricoxib 120mg were superior to placebo for preventing pain, at 8 hours after surgery. Rescue medication intake was significantly lower for drugs groups compared to placebo | No adverse effect observed |
| **DexametHasone (submucosal route) (17)** | | | | |
| Arora *et al*., 2018  (n=2) | Dexamethasone 4mg (n=15)  Dexamethasone 8mg (n=15)  Placebo (n=15) | Pain, swelling and trismus  (2 and 7 days) | Dexamethasone 4mg and 8mg were both superior to placebo for preventing swelling at 2 days after procedure | No adverse effect observed |
| Chugh *et al*., 2018  (n= 3) | Dexamethasone 8mg (n= 23)  Methylprednisolone 40mg (n= 20)  Placebo (n= 17) | Pain, swelling and trismus  (7 days) | Dexamethasone 8mg was superior to methylprednisolone 40mg for reducing swelling outcome. Dexamethasone 8mg was superior to placebo for pain and trismus outcomes | Not reported |
| Deo, 2016  (n= 3) | Dexamethasone 8mg (n= 19)  Placebo (n= 11) | Pain, swelling and trismus  (7 days) | Dexamethasone 8mg was superior to placebo for reducing pain, trismus and swelling outcomes | Not reported |
| Ehsan *et al*., 2014  (n= 3) | Dexamethasone 4 mg (n= 50)  Placebo (n= 50) | Trismus and swelling  (2 and 7 days) | Dexamethasone 4mg was superior to placebo on reducing trismus and swelling outcomes | Not reported |
| Gozali *et al*., 2017  (n=2) | Dexamethasone 8 mg (n= 48)  Placebo (n= 48) | Pain  (1, 2 and 3 days) | Dexamethasone 8mg can reduce postoperative pain after mandibular third molar extraction | Not reported |
| Grossi etal., 2007  (n= 3) | Dexamethasone 4mg (n= 18)  Dexamethasone 8mg (n= 20)  Placebo (n= 23) | Pain, trismus and edema  (1, 2, 3 and 7 days) | Dexamethasone 4mg and 8mg had equal benefit in reducing edema (both were superior to placebo). No difference was observed among the groups for pain and trismus outcomes | Not reported |
| Hadi *et al*., 2020  (n=1) | Dexamethasone 4mg (n= 20)  Placebo (n= 20) | Pain (1 to 7 days); trismus and edema  (1, 2 and 7 days) | Intervention group showed less trismus than the control group. Intervention group showed a significant reduction in pain (1st and 2nd postoperative days) and in edema compared to the control group | Not reported |
| Khalida *et al*., 2017  (n= 2) | Dexamethasone 4mg (n= 20)  Placebo (n= 20) | Swelling and trismus  (2 days) | Dexamethasone 40mg reduced the post-operative discomfort compared to placebo | Not reported |
| Lau *et al*., 2020  (n=1) | Dexamethasone 8mg (SM= 2 mL) (n= 52)  Dexamethasone 8mg (IV= 2 mL) (n= 52) | Pain, swelling and trismus  (2 and 7 days) | There is no difference in pain, swelling and trismus between the groups | Not reported |
| Liaquat *et al*., 2021  (n=1) | Dexamethasone 4mg (n= 75)  Placebo (n= 75) | Pain, and trismus  (1, 2 and 3 days) | Dexamethasone injection significantly reduced pain and trismus on the third postoperative day | Not reported |
| Lim; Ngeow, 2017  (n= 2) | Dexamethasone 4mg (n= 20)  Methylprednisolone 40mg (n= 20)  Placebo (n= 20) | Pain, swelling, trismus and analgesic consumption  (1, 2, 4, 5 and 7 days) | Methylprednisolone 40mg reduced pain and was associated with less analgesic consumption, in 2nd post-operative day. Both drugs reduced swelling and trismus compared to placebo | No adverse effect observed |
| NAIR *et al*., 2013  Nair *et al*., 2013  (n= 2) | Dexamethasone 4mg (n= 50)  Placebo (n= 50) | Pain, swelling and trismus  (2 and 7 days) | Dexamethasone 4mg reduced postoperative edema compared to placebo in 2nd postoperative day | Not reported |
| Saravannan *et al*., 2016  (n= 3) | Dexamethasone 4mg (submucosal)  (n= 20)  Dexamethasone 4mg (intramuscular) (n= 20) | Pain, trismus and swelling  (1, 3 and 7 days) | Submucosal dexamethasone 4mg was superior to intramuscular dexamethasone 4mg for reducing pain, trismus and swelling (up to 7 days) | Not reported |
| Shad *et al*., 2020  (n=2) | Dexamethasone 4mg (n=75)  Placebo (n=75) | Pain, swelling, trismus and quality of life  (2 days) | Dexamethasone 4mg reduced post-operative pain, swelling and trismus, and improved patient quality of life compared to placebo | Not reported |
| Sreesha *et al*., 2020  (n= 1) | Dexamethasone 4mg (SM) (n= 32)  Dexamethasone 4mg (IV) (n= 32) | Pain, swelling, trismus and quality of life  (4 hours, 2 and 7 days) | Postoperative medications have similar effects on both groups regarding the reduction of pain, even though the IV dexamethasone provided a significant difference in pain perception on immediate and 2nd postoperative day | Not reported |
| Syed *et al*., 2017  (n= 1) | Dexamethasone 4mg (n= 20)  Placebo (n= 20) | Pain, trismus and swelling  (1 and 7days) | Dexamethasone 4mg reduced pain, swelling and improvement of mouth opening at the 1st postoperative day | Not reported |
| Warraich *et al*., 2013  (n= 2) | Dexamethasone 4 mg (n= 50)  Placebo (n= 50) | Pain and swelling  (2 and 10 days)  Trismus (2, 10 and 28 days) | Pain was significantly lower in dexamethasone group compared to placebo (2nd to 10th postoperative days). Swelling was lower with the use of dexamethasone (2nd day). No difference was observed between the groups in reduction of trismus | Not reported |
| **DEXAMETHASONE (pterygomandibular route) (4)** | | | | |
| Boonsiriseth *et al*., 2017  (n= 1) | Dexamethasone 8mg (2mL) (n= 31)  Placebo (n= 31) | Pain, trismus and swelling  (2 and 7 days) | Dexamethasone 8mg was superior to placebo for reducing pain, trismus and swelling outcomes for up to 2 days | Not reported |
| Latt *et al*., 2016  (n= 2) | Dexamethasone 8mg (n= 30)  Placebo (n= 30) | Pain  (1, 2 and 3 days)  Trismus and swelling  (2 and 7 days) | No statistical difference was observed between the groups for pain outcome. Dexamethasone 8mg was superior to placebo in swelling and trismus on the 2nd postoperative day | Not reported |
| Moranon *et al*., 2019  (n= 2) | Dexamethasone 8mg (pterygomandibular) (n= 30)  Dexamethasone 8mg (Sublingual) (n= 30) | Pain (1, 2 and 3 days)  Trismus and swelling  (2 and 7 days) | No significant difference was observed between groups in pain and swelling (2nd and 7th postoperative days). However, the pterygomandibular group showed greater degree of limited mouth opening than sublingual group (2nd day after operation) | No adverse effect observed |
| Wanthanont *et al*., 2021  (n= 2) | Dexamethasone 4mg (n= 26)  Dexamethasone 8mg (n= 26) | Pain, trismus and swelling  (2 and 7 days) | Dexamethasone 8mg reduced swelling and pain. However, the effects on trismus and analgesic consumption were similar | No adverse effect observed |
| **DEXAMETHASONE (intramuscular Route - IM) (9)** | | | | |
| Al-Dajani, 2017  (n= 2) | Dexamethasone 0.1mg/kg (deltoid muscle) (n= 32)  No drug (n= 32) | Pain, trismus and swelling  (7 days) | Dexamethasone was superior to no drug for reducing pain, trismus and swelling outcomes | Not reported |
| Antunes *et al*., 2011  (n= 3) | Dexamethasone 8mg (masseter muscle) (n= 67)  Dexamethasone 8mg (oral) (n= 67)  Placebo (n= 67) | Pain, trismus and swelling  (1, 2, 3 and 7 days) | Both dexamethasone groups were superior to placebo for reducing pain, trismus and swelling outcomes following lower third molar extraction. | Not reported |
| Klongnoi *et al*., 2012  (n= 4) | Dexamethasone 8mg (masseter muscle) (n= 20)  Placebo (n= 20) | Pain, trismus and swelling  (7 days) | Dexamethasone 8mg was superior to placebo for reducing pain, trismus and swelling outcomes | Not reported |
| Magrin *et al*., 2010  (n= 1) | Dexamethasone 4mg (masseter muscle) (n= 15)  Placebo (n= 15) | Pain and trismus  (1, 2 and 7 days)  Swelling (1 to 4 days) | Patients in the intervention group reported a decrease in pain, edema, trismus and faster functional recovery compared to the control group | Not reported |
| Mushtaq *et al*., 2011  (n= 1) | Dexamethasone 8mg (masseter muscle) (n= 100)  Placebo (n= 100) | Pain, swelling, trismus and rescue analgesic  (2 and 5 days) | Dexamethasone 8mg was effective in minimizing swelling and pain at all intervals. It resulted in less trismus than controls on day 2 postoperatively, but there was no significant difference among the groups at 5th day | No adverse effect observed |
| Nandini, 2016  (n= 2) | Dexamethasone 8mg (masseter muscle) (n= 10)  Placebo (n= 10) | Pain, trismus and swelling  (1, 2 and 7 days) | Dexamethasone 8mg reduced pain, swelling and trismus for up to 7 days postoperatively with effect superior to placebo | No adverse effect observed |
| Pedersen, 1985  (n= 1) | Dexamethasone 4mg (masseter muscle) (n= 30)  Placebo (n= 30) | Pain, trismus and swelling  (2 and 7 days) | Dexamethasone 4mg equaled placebo for reducing pain, but was superior to placebo for reducing trismus (up to 7 days) and swelling (up to 2 days) | No adverse effect observed |
| Priyanga *et al*., 2021  (n= 2) | Dexamethasone 8mg (IM) (n= 75)  Dexamethasone 8mg (sublingual) (n= 75) | Pain, trismus and swelling  (2, 3 and 7 days) | Sublingual dexamethasone 8mg had superior results in controlling pain, swelling and trismus compared to use of IM route | Not reported |
| Srivastava *et al*., 2021  (n= 1) | Dexamethasone 8mg (masseter muscle) (n= 20)  Methylprednisolone 40mg (masseter muscle) (n= 20)  Placebo (n= 67) | Pain, trismus and swelling  (2 and 7 days) | Dexamethasone 8mg reduced postoperative pain, swelling and trismus compared to other groups | No adverse effect observed |
| **DEXAMETHASONE (intravenous route) (5)** | | | | |
| Agraval *et al*., 2020  (n= 1) | Dexamethasone 8mg IV (n=15)  Dexamethasone 8mg intralesional (n=15)  Placebo (n=15) | Pain, trismus and swelling  (1, 2, 3, 4, 5, 6 and 7 days) | Patients receiving intralesional steroids experienced lesser amount of pain and trismus as compared to those who received IV steroids. Drugs group patients showed a lower degree of pain as compared to placebo group, but only on the day of surgical. Less reduction in incisal mouth opening was reported in intralesional group as compared to IV group on the 7th postoperative day | Not reported |
| Claseman *et al*., 1998  (n= 1) | Dexamethasone 8mg (n= 8)  Dexamethasone 8mg +  ketorolac 30mg (n= 9)  ketorolac 30mg (n= 9)  Placebo (n= 8) | Pain and rescue medication  (4, 6, 8 and 10 hours) | All drug groups were superior to placebo for reducing pain and rescue medication consumption for up to 10 hours | No adverse effect observed |
| Hiriyanna *et al*., 2021  (n= 2) | Dexamethasone 8mg (IV) (n= 17)  Dexamethasone 8mg (submucosal) (n= 16) | Pain, trismus, swelling and quality of life  (2 and 7 days) | No statistical difference between groups was detected in promoting change on 2nd and 7th days | Not reported |
| Neupertt III *et al*., 1992  (n = 2) | Dexamethasone 4mg (n= 60)  Placebo (n = 60 | Pain, trismus and swelling  (1 and 7 days) | Pain and trismus were reduced in dexamethasone group compared to placebo. No statistical difference was observed between the groups for swelling | No adverse effect observed |
| Tiwana *et al*., 2005  (n= 1) | Dexamethasone 8mg (n= 37)  Methylprednisolone 40mg (n= 23)  Placebo (n= 60) | Quality of life  (1 and 14 days) | Intravenous corticosteroids had a positive, but limited impact on quality of life | Post-surgical nausea was lower for patients who had corticosteroid treatment compared to placebo |
| **METHYLPREDNISOLONE (oral route) (3)** | | | | |
| Acham *et al*., 2013  (n= 4) | Methylprednisolone 40mg (n= 16)  Methylprednisolone 60mg (n= 16)  Methylprednisolone 80mg (n= 16)  Placebo (n= 16) | Pain, trismus and swelling  (7 days) | Methylprednisolone 40mg, 60mg and 80mg was superior to placebo for reducing pain, trismus and swelling outcomes | No adverse effect observed |
| Miles; Desjardins, 1993  (n= 3) | Methylprednisolone 16mg (oral) (n= 11)  Methylprednisolone 20mg (intravenous) (n= 11)  Placebo (n=11) | Swelling, trismus and rescue medication  (1, 2, 3, 4 and 7 days) | Methylprednisolone 16mg and 20mg had better results than placebo for swelling and trismus outcomes. All groups required the highest number of pill backup analgesics on the day of surgery | No adverse effect observed |
| Mitchell; Seymour, 1985  (n= 1) | Methylprednisolone 80mg (n= 20)  Aspirin 1000mg (n= 20)  Ibuprofen (400mg) + codeine (30mg) (n= 20)  Placebo (n= 20) | Pain  (1, 2, 3 and 5 hours)  Swelling and trismus  (2 and 7 days) | Aspirin and ibuprofen/codeine groups were superior to methylprednisolone and placebo group for reducing pain. Swelling outcome was equal among the groups. Methylprednisolone promoted a significantly reduced period of post-operative trismus | Drowsiness may have been due to the high dose of codeine |
| **METHYLPREDNISOLONE (intramuscular route) (3)** | | | | |
| Gholami *et al*., 2021  (n=1) | Methylprednisolone 40mg (masseter muscle) (n= 20)  Methylprednisolone 40mg (gluteal muscle) (n= 20)  Placebo (n= 20) | Pain  (1, 5 and 7 days)  Swelling and trismus  (5 and 7 days) | The gluteal group had the lowest mean pain level (1, 5 and 7 days after surgery). Placebo group had the highest mean edema and the gluteal group had the lowest (5 and 7 days after surgery). The lowest mean amount of trismus was documented in the control group (5 and 7 days after surgery) | Not reported |
| LARSEN *et al*., 2021  Larsen *et al*., 2021  (n=1) | Methylprednisolone 20mg (n= 26)  Methylprednisolone 30mg (n= 26)  Methylprednisolone 40mg (n= 26)  Placebo (n= 26) | Pain, trismus and quality of life  (1, 3, and 7 days) | Patients receiving methylprednisolone 30mg reported a tendency toward lower pain compared with other groups. No significant differences were observed among the groups for trismus or quality of life outcomes | Not reported |
| Selvaraj *et al*., 2014  (n=1) | Methylprednisolone 40mg (masseter muscle) (n= 10)  Methylprednisolone 40mg (gluteal muscle) (n= 10) | Pain  (1, 2, and 3 days)  Swelling and trismus  (2 and 7 days) | No significant difference between the groups was observed in terms of pain, swelling or trismus | Not reported |
| **METHYLPREDNISOLONE (intravenous route) (6)** | | | | |
| Beirne; Holander, 1986  (n= 4) | Methylprednisolone 125mg (n= 15)  Placebo (n= 16) | Pain, trismus and swelling  (1, 2, 3, 4 and 7 days) | Methylprednisolone 125mg was superior to placebo for reducing pain (up 4 days) and swelling (up to 7 days) outcomes. No statistical difference was observed between the groups for trismus | Not reported |
| Esen *et al*., 1999  (n= 4) | Methylprednisolone 125mg (n= 20)  Placebo (n= 20) | Rescue medication, swelling and trismus  (1 and 7 days) | Methylprednisolone 125mg group used less pain medication compared to placebo, but the difference was not significant (up to 1 day after surgery). Trismus (up to 7 days) and swelling (up to 2 days) outcomes were superior to placebo | No adverse effect observed |
| Holland, 1987  (n= 1) | Methylprednisolone 40mg (n= 20)  Placebo (n= 20) | Pain, swelling and healing  (1 and 7 days) | Methylprednisolone 40mg was superior to placebo for reducing pain and swelling outcomes, but healing was similar in both groups | No adverse effect observed |
| Hyrkas *et al*., 1993  (n= 5) | Methylprednisolone 40mg (IV) + diclofenac 100mg (oral) + diclofenac 50mg (oral) (n= 36)  Saline solution (IV) + diclofenac 100mg (oral) + diclofenac 50mg (oral) (n= 36) | Pain, trismus and rescue medication  (4 hours; 1 and 2 days) | Methylprednisolone/diclofenac was superior to control group for pain outcome. There was no significant difference between the groups with respect to mouth opening. Use of rescue medication was lower in methylprednisolone + diclofenac group than in control group | No adverse effect observed |
| Ilhan *et al*., 2014  (n= 2) | Methylprednisolone 80mg (n= 20)  Tenoxicam 20mg (n= 20)  Placebo (n= 20) | Pain, swelling and trismus  (2 days) | Methylprednisolone 80mg was superior to tenoxicam 20mg for control of trismus. No significant difference was observed for pain or swelling | No adverse effect observed |
| Ústun *et al*., 2003  (n= 2) | Methylprednisolone 1.5mg/kg (n= 20)  Methylprednisolone 3mg/kg (n= 20) | Pain, swelling and trismus  (2 and 7 days) | No statistical difference was observed between the groups for pain, trismus or swelling outcomes | Not reported |
| **PREDNISOLONE (oral route) (3)** | | | | |
| Ibikunle; Adeyemo; Ladeinde, 2016  (n= 2) | Prednisolone 40mg (oral) (n= 62)  Prednisolone 40mg (submucosal) (n= 62)  Placebo (n= 62) | Pain, swelling, trismus and quality of life  (1, 2 and 3 days) | Submucosal and oral prednisolone was superior to placebo for reducing postoperative pain, edema and trismus. Submucosal prednisolone promoted significant improvement in quality of life compared to oral prednisolone | Not reported |
| Kang *et al*., 2010  (n= 5) | Prednisolone 10mg (n= 96)  Prednisolone 20mg (n= 60)  Placebo (n= 64) | Pain, swelling and trismus  (6 days) | No statistical difference was observed among the groups | No adverse effect observed |
| Lim, 1996  (n= 1) | Prednisolone 10mg + diclofenac 50mg (n= not reported)  Diclofenac 50mg (n= not reported) | Pain, trismus and swelling  (NR) | No significant difference was observed between groups for trismus and pain. Prednisolone 10mg + diclofenac 50mg was superior to diclofenac 50mg for swelling outcome | Not reported |

**D - Description of randomized clinical trials of adults undergoing third molar surgery, dental implants and periodontal surgery and in preemptive use of NSAIDS and opioid (n= 40)**

| **Clinical trials**  (Number of SR) | **I: Intervention**  **C: Control**  (Number of participants) | **Outcomes**  (Follow-up) | **Effectiveness outcomes** | **Safety outcomes** |
| --- | --- | --- | --- | --- |
| **ACETAMINOPHEN (oral route) (3)** | | | | |
| Asadi *et al*., 2017  (n=1) | I: Acetaminophen (600 mg) + ibuprofen (400mg) + cafein (15 mg)  C: Placebo | Pain  (2, 4, 6 and 8 hours) | At any time, the mean pain score was significantly lower in the group who received combination regimen than the control group | No adverse effect observed |
| Cristalli *et al*., 2017  (n=1) | I: Acetaminophen l (500 mg) + codein (30 mg) (n=32)  C: placebo (n=32) | Pain  Time of the first request for rescue analgesic  (2 days) | The pain intensity score on the first day was significantly lower in the analgesic group than in the placebo group. The time to using rescue therapy was significantly longer in the analgesic group than in the placebo group. The number of paracetamol-codeine tablets used postoperatively did not differ between the analgesic and placebo groups | No adverse effect observed |
| Liashek; Desjardins; Triplett, 1987  (n= 1) | I: Acetaminophen 650mg (n= 12)  I: Propoxyphene 50mg (n= 10)  I: Acetaminophen 650mg + Propoxyphene 50mg (n= 11)  C: Placebo (n= 12) | Pain  (4 hours) | Acetaminophen 650mg + propoxyphene 50mg was superior to each drug alone and to placebo for reducing pain outcome | No adverse effect observed |
| **CELECOXIB (oral route) (3)** | | | | |
| Khan *et al*., 2002  (n=1) | I: Celecoxib 200mg (n= 37)  I: Ibuprofen 600mg (n= 30)  C: Placebo (n= 36) | Pain  (4 hours) | Pain intensity rating was lower with celecoxib and ibuprofen compared to placebo | Not reported |
| Xie *et al*., 2020  (n=2) | I: Celecoxib 200 mg  (n= 30)  C: Acetaminophen 500mg  (n = 30) | Pain  (4, 6, 8, and 12 hours)  Rescue medication (12 hours) | Celecoxib 200mg had lower postoperative pain scores compared to acetaminophen 500mg, at 4, 6, 8, and 12 hours. The number of patients who require analgesics and rescue analgesic was lower in celecoxib 200mg group than acetaminophen 500mg group | Not reported |
| Al-Sukhun *et al*., 2012  (n= 3) | I: Celecoxib 200mg (n= 48)  I: Ibuprofen 400mg (n= 48)  C: Placebo (n= 48) | Pain  (24 hours) | Celecoxib 200mg was superior both to Ibuprofen 400mg and to placebo for reducing pain | Adverse effects were nausea (25%, 8.3%, and 18.1%), headache (14.1%, 9.2% and 8.7%) and vomiting (11.3%, 1.3% and 9.1%) in placebo, celecoxib, and ibuprofen groups, respectively |
| **DEXKETOPROFEN (oral route) (1)** | | | | |
| Sanchez-Pérez *et al*., 2018**  (n= 1) | I: Dexketoprofen 25 mg (n=41)  C: Placebo (n=42) | Pain  (2, 8, 12 hours; 1, 2, 3 and 7 days) | Dexketoprofen 25 mg was superior to placebo for reducing immediate postoperative pain | No adverse effect observed |
| **DICLOFENAC (oral route) (4)** | | | | |
| Akbulut *et al*., 2014  (n= 2) | I: Diclofenac potassium 50mg (n=14)  I: Naproxen 275mg (n= 14)  I: Etodolac 200mg (n= 14) | Pain  (12 hours; 1, 2, 3, 5 and 7 days)  Swelling and trismus  (2 and 7 days) | All drugs were effective for controlling pain and trismus. Diclofenac 50mg was superior to naproxen 275mg and etodolac 200mg for reducing swelling on 2nd postoperative day | No adverse effect observed |
| Aoki *et al*., 2006a  (n=1) | I: Dextromethorphan 30mg (n=37)  I: Diclofenac 25mg (n=38)  C: Placebo (n=36) | Pain and rescue medication  (1, 7 and 14 days) | All drugs were effective for controlling pain until the 7th day after surgery. Compared with the placebo, patients premedicated with dextromethorphan 30mg, required significantly fewer analgesics for postoperative pain | No adverse effect observed |
| Cigerim; Eroglu, 2018  (n= 1) | I: Diclofenac potassium 50mg (n= 30)  I: Naproxen 550 mg + codeine 30mg (n= 30)  I: Benzydamine 50mg (n= 30) | Pain  (2, 8, 10, 12, 18, and 24 hours)  Swelling and trismus  (2 and 7 days) | Naproxen 550mg + codeine 30mg were superior to diclofenac potassium 50mg and benzydamine 50mg for reducing pain (up to 24 hours) and edema and trismus outcomes (up to 7 days) | No adverse effect observed |
| Orozco-Solis *et al*., 2016  (n= 3) | I: Diclofenac sodium 100mg (n=18)  I: Meloxicam 15mg (n=18)  C: Placebo (n=18) | Pain (6 and 24 hours)  Swelling and trismus  (1, 2, 3 and 7 days) | Meloxicam 15mg was superior to diclofenac sodium 100mg and placebo for reducing pain (up to 5 hours) and trismus outcomes (up to 7 days). No statistical difference was observed among the groups for swelling (up to 7 days) | No adverse effect observed |
| **DICLOFENAC (intramuscular route) (1)** | | | | |
| Mony; Kukarni; Shetty, 2016  (n= 1) | I: Diclofenac 30mg (n=25)  C: Ketorolac 75mg (n=25) | Pain and rescue medication  (12 hours) | Parenteral ketorolac resulted in prolonged analgesia. This was evident in terms of consumption of rescue medication, which was significantly less in the ketorolac than the diclofenac group | No adverse effect observed |
| **DIFLUNISAL (oral route) (2)** | | | | |
| Pektas *et al*., 2007  (n= 2) | I: Diflunisal1000mg (n=40)  I: Lornoxicam 16mg (n=40) | Pain  (2, 4, 6, 12 and 24 hours) | No statistical difference was observed be­tween the groups. Both NSAIDS proved highly effective | Mild and self-limiting adverse effects were observed that required no treatment |
| Costa *et al*., 2015  (n= 2) | I: Etoricoxib 120mg (n=18)  C: Placebo (n=18) | Pain, rescue medication, swelling and trismus  (0, 2, 4, 6, 8, 10 and 12 hours; 1, 2 3, 5 and 7 days) | Etoricoxib 120mg was superior to placebo for reducing pain outcome (up to 8 hours). At 8 hours after surgery, all patients who had received placebo consumed rescue analgesics, while 22.2% of the patients who had received etoricoxib needed no rescue medication during the 7 days after surgery. No statistical difference was observed between the groups for swelling or trismus outcomes | No significant difference was observed in the number of participants reporting adverse effects. No serious adverse events were observed |
| **FENBUFEN (oral route) (1)** | | | | |
| Smith; Brook, 1990  (n=1) | I: Fenbufen 450mg (n=20)  C: Placebo (n=18) | Pain  (2, 8, and 12h; 1 and 3 days) | Preoperative administration of Fenbufen 450mg reduced immediate postoperative pain, but overall pain experience and morbidity was not improved over placebo. | No adverse effect observed |
| **IBUPROFEN (oral route) (7)** | | | | |
| Albuquerque *et al*., 2017  (n= 2) | I: Ibuprofen 400mg (n= 12)  I: Etoricoxib 120mg (n= 12)  C: Placebo (n=12) | Pain  (0, 2, 4, 6, 8, 10 12 and 24 hours; 5 and 7 days)  Swelling and trismus  (1, 3 and 7 days) | Etoricoxib 120mg was superior to both ibuprofen 400mg and placebo for reducing pain. Trismus was reduced in the group treated with ibuprofen on 7th postoperative day and in the group treated with etoricoxib at 1, 3 and 7 days after surgery compared to placebo group. Both drugs were superior to placebo for swelling (3 days) | No drug-related side effects were observed |
| Borea; Monopoli; Colantoni, 1996  (n= 1) | I: Ibuprofen + arginine 400mg (n= 47)  I: Naproxen 550mg (n= 46)  C: Placebo (n= 46) | Pain and rescue medication  (0, 1, 2, 3, 4 and 5 hours) | No statistical difference was observed among the groups up to 5 hours. Rescue medication was required in significantly fewer patients in the active treatment groups than in the placebo group | Adverse events reported were: with the use of ibuprofen + arginine (dizziness); naproxen (moderate nausea, syncope and dyspnea); and placebo (somnolence, fatigue, excessive secretion of saliva and difficulty concentrating) |
| Chiu; Cheung, 2005  (n= 1) | I: Ibuprofen 400mg (n= 33)  I: Rofecoxib 50mg (n= 33)  C: Placebo (n= 32) | Pain and rescue medication  (6 and 48 hours) | No statistical difference was observed among the groups for pain (up to 6 hours) or consumption of rescue pain medication (up to 48 hours) | Ibuprofen group reported nausea |
| Lustenberger; Gratz; Mutzbauer, 2011  (n= 1) | I: Ibuprofen 400mg (n= 10)  I: Lornoxicam 8mg (n= 10) | Pain and rescue medication  (2 and 6 hours; 1, 2, 3 and 4 days) | No statistical difference was observed between the groups. Both NSAIDs proved effective in the management of pain for up to 3 days | No significant difference was observed between the groups |
| Morse; Tump; Kevelham, 2006  (n= 2) | I: Ibuprofen 400mg (n= 16)  I: Rofecoxib 50mg (n= 16)  C: Placebo (n=16) | Pain  (6 hours) | No statistical difference was observed among the groups for pain outcome. | No drug-related side effects were observed. |
| **Pereira *et al*., 2020  (n= 1) | I: Ibuprofen 600mg (n= 27)  C: Placebo (n= 27) | Pain and rescue medication  (1, 6 and12 hours; 1, 2 and 3 days) | Ibuprofen 600mg was superior to placebo for reducing pain outcome.  Rescue medication was required in significantly fewer patients in the treatment groups than the placebo group | No drug-related side effects were observed |
| *Singh *et al*., 2020  (n= 1) | I: Ibuprofen 400mg (n=10)  C: Placebo (n=10) | Pain  (Information not found) | Ibuprofen significantly reduced intensity of pain as compared with placebo | Not reported |
| **IBUPROFEN (intravenous route) (1)** | | | | |
| Kuupeli; Gulnahar, 2019  (n=1) | I: Ibuprofen 800mg + dexketoprofen 50mg (n= 20)  I: Ibuprofen 800mg (n= 20)  C: Placebo (n= 20) | Pain  (1, 2, 4 and 48 hours) | Intravenous ibuprofen (alone or with dexketoprofen) had the same perioper­ative analgesic efficacy as placebo (up to 24 hours) | Not reported |
| **KETOROLAC (oral route) (2)** | | | | |
| *Hungund; Thakkar, 2011  (n= 1) | I: ketorolac 10mg (n= 40)  C: Placebo (n= 40) | Pain  (2 hours) | ketorolac 10mg was superior to placebo for reducing pain outcome | No drug-related side effects were observed |
| *Trombelli *et al*., 1996  (n= 2) | I: ketorolac 20mg (n= 22)  C: Placebo (n= 21) | Pain and rescue medication  (10 hours) | ketorolac 20mg was superior to placebo for reducing pain. No statistical difference was observed between the groups in consumption of rescue pain medication | No drug-related side effects were observed |
| **KETOROLAC (intramuscular route) (1)** | | | | |
| Shah *et al*., 2013  (n= 1) | I: Ketorolac 30mg (n= 50)  C: Tramadol 50mg (n= 50)  C: Placebo (n= 50) | Pain  (12 hours) | ketorolac 30mg was superior to tramadol 50mg and to placebo for reducing pain outcome | In tramadol group, 4 patients reported nausea, swelling and respiratory depression |
| **KETOROLAC (intravenous route) (3)** | | | | |
| Ong; Tan, 2004  (n= 1) | I: Ketorolac 30mg (n= 32)  I: Tramadol 50mg (n= 32) | Pain  (12 hours) | ketorolac 30mg was superior to tramadol 50mg for reducing pain outcome | No drug-related side effects were observed |
| Gutta; Koehn; James, 2013  (n= 1) | I: Ketorolac 30mg (n= 38)  C: placebo (2ml) (n= 27) | Pain  (4 and 8 hours) | ketorolac 30mg was superior to placebo for reducing pain outcome | Not reported |
| Gopalraju *et al*., 2014  (n= 1) | I: Ketorolac 30mg (n= 40)  I: Tramadol 50mg (n= 40) | Pain  (12 hours) | ketorolac 30mg was superior to tramadol 50mg for reducing pain outcome | No drug-related side effect was observed |
| **MELOXICAM (oral route) (3)** | | | | |
| Oki *et al*., 2006b  (n= 3) | I: Meloxicam 10mg (n= 31)  I: Ampiroxicam 27mg (n= 30)  C: Placebo (n=30) | Pain and trismus  (1, 7 and 14 days) | Meloxicam 10mg was superior to ampiroxicam 27mg and to placebo for reducing pain outcome. No statistical difference was observed among the groups for the trismus outcome | The incidence of adverse effect did not differ significantly among groups |
| De Menezes; Cury, 2010  (n= 1) | I: Meloxicam 7.5mg (n= 20)  I: Nimesulide 100 mg (n= 20) | Pain  (8, 12 and 24 hours)  Trismus and swelling  (1, 2 and 3 days) | Pain control was similar in both treatment groups. Nimesulide was more effective than meloxicam for the control of swelling and trismus | No drug-related side effect was observed |
| Sener *et al*., 2005  (n= 1) | I: Meloxicam 7.5mg (n= 30)  I: Diflunisal 500mg (n= 30)  I: Naproxen 550mg (n= 30)  I: Acetaminophen 500mg (n=30)  I: Rofecoxib 12.5mg (n= 30) | Pain  (2, 4, 6 and 12 hours) | Diflunisal 500mg was superior to other groups for reducing pain outcome | No significant difference in the prevalence of nausea, vomiting, allergy or gastrointestinal symptoms was found among the groups |
| **MELOXICAM (intramuscular route) (2)** | | | | |
| Isiordia-Espinoza *et al*., 2012a  (n= 1) | I: Meloxicam 15mg (n=15)  I: Tramadol 50mg (n=15) | Pain, trismus and swelling  (6 hours; 1, 2 and 3 days) | Meloxicam 15mg was superior to tramadol 50mg for reducing pain outcome | No drug-related side effect was observed |
| Isiordia-Espinoza *et al*., 2012b  (n= 1) | I: Meloxicam 15mg (n=17)  I: Tramadol 50mg (n=17)  C: Meloxicam 7.5mg + tramadol 25mg (n=17) | Pain  (12 hours) | Meloxicam 7.5mg + tramadol 25mg had a similar analgesic effect to meloxicam 15mg, but was better than tramadol 50 mg for reducing pain outcome | Combination of meloxicam with tramadol showed some adverse effects |
| **NIMESULIDE (oral route) (2)** | | | | |
| Costa-Araujo *et al*., 2012  (n=2) | I: Nimesulide 100mg (n= 47)  C: Tramadol 100mg (n=47) | Pain and rescue medication  (5, 6, 24, 36, 48, 60, 72 hours) | Oral nimesulide (100mg) and tramadol (100mg) demonstrated similar results with regard to postoperative pain.83% of the patients needed the rescue drug in the postoperative period when using tramadol, whereas only 68.1% needed the rescue drug when nimesulide was used, although this difference did not achieve statistical significance | 19.1% of the patient reported vomiting and 10.6% reported nausea in the postoperative period of tramadol (100 mg). None of the patients reported any adverse effect with the use of nimesulide |
| Neychev; Chenchev; Simitchiev, 2017  (n= 1) | I: Nimesulide 100mg (n= 30)  C: Metamizole 500mg (n= 30)  C: Placebo (n= 20) | Pain  (2, 6 and 12 and 24 hours; 2 and 3 days) | Nimesulide 100mg was superior to metamizole 500mg and to placebo for reducing pain outcome (up to 24 hours) | Not reported |
| **PARECOXIB (intramuscular route) (1)** | | | | |
| Desjardins *et al*., 2001  (n= 1) | I: Parecoxib 20mg (n= 56)  I: Parecoxib 40mg (n= 56)  I: Parecoxib 80mg (n= 56)  C: Placebo (n=56) | Pain and rescue medication  (Intervals from 2 to 24 hours) | Parecoxib (any dose) was superior to placebo for reducing pain for up to 24 hours.  Rescue medication was required in significantly fewer patients in the treatment groups than placebo group | 39% of patients in placebo group, 30% in parecoxib 20mg group, 34% in parecoxib 40mg group, and 25% in parecoxib 80mg group had adverse events. Most of these events were of mild-to-moderate severity and included nausea, headache, dizziness and vomiting |
| **ROFECOXIB (oral route) (1)** | | | | |
| Fornai *et al*., 2006  (n=1) | I: Rofecoxib 50mg  I: Naproxeno 550mg  C: Placebo | Pain  (Every 30min until 6 hours) | Pain intensity in the placebo group increased throughout the observation period. Naproxen 50mg and rofecoxib 550mg reduced pain until 6 hours | No drug-related side effects were observed |
| **TENOXICAM (oral route) (1)** | | | | |
| Cheung; Rodrigo, 1992  (n=1) | I: Tenoxicam 40mg (n=15)  C: Acetaminophen 1000mg (n=15) | Pain  (Information not found) | Both acetaminophen and tenoxicam relieved pain, but tenoxicam provided no advantage in terms of duration of action | Not reported |
| **TRAMADOL (oral route) (1)** | | | | |
| Kanto *et al*., 2005  (n=1) | I: Tramadol 100mg (n=20)  C: Placebo (n=20) | Pain and rescue medication  (0.5, 1, and 2 hours) | Tramadol 100mg improved the quality of operating. The effect persisted post operatively on the day of operation as a delayed and lower need for analgesics during the first operation compared with placebo | 3 patients reported nausea with the use of tramadol 100mg |

*Periodontal surgery. **Dental implant surgery.

**REFERENCES used in flies S3 and S4**

ACHAM, S. *et al*. Beneficial effect of methylprednisolone after mandibular third molar surgery: A randomized, double-blind, placebo-controlled split-mouth trial. **Clinical Oral Investigations**, v. 17, n. 7, p. 1693–1700, 2013.

AGOSTINHO, C. N. EWTO. L. F. ELICI. *et al*. The efficacy of 2 different doses of dexamethasone to control postoperative swelling, trismus, and pain after third molar extractions. **General dentistry**, v. 62, n. 6, p. e1–e5, 2014.

AKBULUT, N. *et al*. Comparison of the effect of naproxen, etodolac and diclofenac on postoperative sequels following third molar surgery: A randomised, double-blind, crossover study. **Medicina Oral, Patologia Oral y Cirugia Bucal**, v. 19, n. 2, p. 149–156, 2014.

AL-DAJANI, M. Can Preoperative Intramuscular Single-Dose Dexamethasone Improve Patient-Centered Outcomes Following Third Molar Surgery? **Journal of Oral and Maxillofacial Surgery**, v. 75, n. 8, p. 1616–1626, 2017.

AL-SUKHUN, J. *et al*. Preemptive analgesic effect of low doses of celecoxib is superior to low doses of traditional nonsteroidal anti-inflammatory drugs. **Journal of Craniofacial Surgery**, v. 23, n. 2, p. 526–529, 2012.

ALBUQUERQUE, A. F. M. *et al*. Effect of pre-emptive analgesia on clinical parameters and tissue levels of TNF-α and IL-1β in third molar surgery: a triple-blind, randomized, placebo-controlled study. **International Journal of Oral and Maxillofacial Surgery**, v. 46, n. 12, p. 1615–1625, 2017.

ALCANTARA, C. E. . *et al*. Pre-Emptive Effect of Dexamethasone and Diclofenac Sodium Associated With Codeine on Pain, Swelling, and Trismus After Third Molar Surgery: A Split-Mouth, Randomized, Triple-Blind, Controlled Clinical Trial. **Journal of Oral and Maxillofacial Surgery**, v. 76, n. 1, p. 60–66, 2013.

ANTUNES, A. A. *et al*. Effect of two routes of administration of dexamethasone on pain, edema, and trismus in impacted lower third molar surgery. **Oral and Maxillofacial Surgery**, v. 15, n. 4, p. 217–223, 2011.

AOKI, T. *et al*. Premedication with cyclooxygenase-2 inhibitor meloxicam reduced postoperative pain in patients after oral surgery. **International Journal of Oral and Maxillofacial Surgery**, v. 35, n. 7, p. 613–617, 2006.

BAUER, H. C. *et al*. Assessment of preemptive analgesia with ibuprofen coadministered or not with dexamethasone in third molar surgery: A randomized double-blind controlled clinical trial. **Oral and Maxillofacial Surgery**, v. 17, n. 3, p. 165–171, 2013.

BAXENDALE, B. R.; VATER, M.; LAVERY, K. M. Dexamethasone reduces pain and swelling following extraction of third molar teeth. **Anaesthesia**, v. 48, n. 11, p. 961–964, 1993.

BEIRNE, O. R.; HOLLANDER, B. The effect of methlyprednisolone on pain, trismus, and swelling after removal of third molars. **Oral Surgery, Oral Medicine, Oral Pathology**, v. 61, n. 2, p. 134–138, 1986.

BHARGAVA, D.; SREEKUMAR, K.; DESHPANDE, A. Effects of intra-space injection of Twin mix versus intraoral-submucosal, intramuscular, intravenous and per-oral administration of dexamethasone on post-operative sequelae after mandibular impacted third molar surgery: a preliminary clinical comparative . **Oral and maxillofacial surgery**, v. 18, n. 3, p. 293–296, 2014.

BOONSIRISETH, K. *et al*. Dexamethasone injection into the pterygomandibular space in lower third molar surgery. **International Journal of Oral and Maxillofacial Surgery**, v. 46, n. 7, p. 899–904, 2017.

BOREA, G.; MONOPOLI, R.; COLANTONI, A. Ibuprofen arginine vs naproxen sodium as prophylactic oral treatment of pain due to dental surgery. A randomised double-blind double-dummy placebo-controlled multicentre study. **Clinical Drug Investigation**, v. 11, n. SUPPL. 1, p. 33–40, 1996.

BORTOLUZZI, M. C. *et al*. A Single Dose of Amoxicillin and Dexamethasone for Prevention of Postoperative Complications in Third Molar Surgery: A Randomized, Double-Blind, Placebo Controlled Clinical Trial. **Journal of Clinical Medicine Research**, v. 5, n. 1, p. 26–33, 2013.

CHIU, W. K.; CHEUNG, L. K. Efficacy of preoperative oral rofecoxib in pain control for third molar surgery. **Oral Surgery, Oral Medicine, Oral Pathology, Oral Radiology and Endodontology**, v. 99, n. 6, p. 47–53, 2005.

CHUGH, A. *et al*. Submucosal injection of dexamethasone and methylprednisolone for the control of postoperative sequelae after third molar surgery: randomized controlled trial. **International Journal of Oral and Maxillofacial Surgery**, v. 47, n. 2, p. 228–233, 2018.

CIGERIM, L.; EROGLU, C. N. Comparison of Clinical Efficacies of Preoperatively Initiated Naproxen Sodium–Codeine Phosphate in Combination, Diclofenac Potassium, and Benzydamine Hydrochloride for Pain, Edema, and Trismus After Extraction of Impacted Lower Third Molar: A Randomized D. **Journal of Oral and Maxillofacial Surgery**, v. 76, n. 3, p. 495–502, 2018.

CLASEMAN, T. S. *et al*. A clinical evaluation of the analgesic efficacy of preoperative administration of ketorolac and dexamethasone following surgical removal of third molars. **Anesthesia progress**, v. 45, n. 3, p. 110–6, 1998.

COSTA, F. W. G. *et al*. A split-mouth, randomized, triple-blind, placebo-controlled study to analyze the pre-emptive effect of etoricoxib 120 mg on inflammatory events following removal of unerupted mandibular third molars. **International Journal of Oral and Maxillofacial Surgery**, v. 44, n. 9, p. 1166–1174, 2015.

DARAWADE, D. A. *et al*. In search of a better option: dexamethasone versus methylprednisolone in third molar impaction surgery. **Journal of international oral health : JIOH**, v. 6, n. 6, p. 14–7, 2014.

DE MENEZES, S. A. F.; CURY, P. R. Efficacy of nimesulide versus meloxicam in the control of pain, swelling and trismus following extraction of impacted lower third molar. **International Journal of Oral and Maxillofacial Surgery**, v. 39, n. 6, p. 580–584, 2010.

DE SOUSA SANTOS, J. A. S. *et al*. Comparative study of tramadol combined with dexamethasone and diclofenac sodium in third-molar surgery. **Journal of Cranio-Maxillofacial Surgery**, v. 40, n. 8, p. 694–700, 2012.

DEO, S. P. Single-Dose of Submucosal Injection of Dexamethasone Affects the Post Operative Quality of Life After Third Molar Surgery. **Journal of Maxillofacial and Oral Surgery**, v. 15, n. 3, p. 367–375, 2016.

DESJARDINS, P. J. *et al*. The injectable cyclooxygenase-2-specific inhibitor parecoxib sodium has analgesic efficacy when administered preoperatively. **Anesthesia and Analgesia**, v. 93, n. 3, p. 721–727, 2001.

EHSAN, A. *et al*. Effects of pre-operative submucosal dexamethasone injection on the postoperative swelling and trismus following surgical extraction of mandibular third molar. **Journal of the College of Physicians and Surgeons Pakistan**, v. 24, n. 7, p. 489–492, 2014.

ESEN, E.; TAŞAR, F.; AKHAN, O. Determination of the anti-inflammatory effects of methylprednisolone on the sequelae of third molar surgery. **Journal of Oral and Maxillofacial Surgery**, v. 57, n. 10, p. 1201–1206, 1999.

GOPALRAJU, P. *et al*. Comparative study of intravenous Tramadol versus Ketorolac for preventing postoperative pain after third molar surgery - A prospective randomized study. **Journal of Cranio-Maxillofacial Surgery**, v. 42, n. 5, p. 629–633, 2014.

GROSSI, G. B. *et al*. Effect of Submucosal Injection of Dexamethasone on Postoperative Discomfort After Third Molar Surgery: A Prospective Study. **Journal of Oral and Maxillofacial Surgery**, v. 65, n. 11, p. 2218–2226, 2007.

GUTTA, R.; KOEHN, C. R.; JAMES, L. E. Does ketorolac have a preemptive analgesic effect? A randomized, double-blind, control study. **Journal of Oral and Maxillofacial Surgery**, v. 71, n. 12, p. 2029–2034, 2013.

HOLAND, C. S. M.D.s., F.D.S.R.C.S. **British Journal of Oral and Maxillofacial Surgery**, v. 25, p. 293–299, 1986.

HUNGUND, S.; THAKKAR, R. Effect of pretreatment with ketorolac tromethamine on operative pain during periodontal surgery: A case-control study. **Journal of Indian Society of Periodontology**, v. 15, n. 1, p. 55–58, 2011.

HYRKÄS, T. *et al*. A comparison of diclofenac with and without single-dose intravenous steroid to prevent postoperative pain after third molar removal. **Journal of Oral and Maxillofacial Surgery**, v. 51, n. 6, p. 634–636, 1993.

IBIKUNLE, A. A.; ADEYEMO, W. L.; LADEINDE, A. L. Oral health-related quality of life following third molar surgery with either oral administration or submucosal injection of prednisolone. **Oral and Maxillofacial Surgery**, v. 20, n. 4, p. 343–352, 2016.

ILHAN, O. *et al*. A comparison of the effects of methylprednisolone and tenoxicam on pain, edema, and trismus after impacted lower third molar extraction. **Medical Science Monitor**, v. 20, p. 147–152, 2014.

ISIORDIA-ESPINOZA, M. A. *et al*. Pre-emptive analgesia with the combination of tramadol plus meloxicam for third molar surgery: A pilot study. **British Journal of Oral and Maxillofacial Surgery**, v. 50, n. 7, p. 673–677, 2012a.

ISIORDIA-ESPINOZA, M. A. *et al*. Pre-emptive analgesic effectiveness of meloxicam versus tramadol after mandibular third molar surgery: A pilot study. **Journal of Oral and Maxillofacial Surgery**, v. 70, n. 1, p. 31–36, 2012b.

KANG, S. H. *et al*. Effect of preoperative prednisolone on clinical postoperative symptoms after surgical extractions of mandibular third molars. **Australian Dental Journal**, v. 55, n. 4, p. 462–467, 2010.

KLONGNOI, B. *et al*. Effect of single dose preoperative intramuscular dexamethasone injection on lower impacted third molar surgery. **International Journal of Oral and Maxillofacial Surgery**, v. 41, n. 3, p. 376–379, 2012.

KONUGANTI, K.; RANGARAJ, M.; ELIZABETH, A. Pre-emptive 8 mg dexamethasone and 120 mg etoricoxib for pain prevention after periodontal surgery: A randomised controlled clinical trial. **Journal of Indian Society of Periodontology**, v. 19, n. 4, p. 474–476, 2015.

LAUREANO FILHO, J. R. *et al*. Clinical comparative study of the effectiveness of two dosages of Dexamethasone to control postoperative swelling, trismus and pain after the surgical extraction of mandibular impacted third molars. **Medicina Oral, Patologia Oral y Cirugia Bucal**, v. 13, n. 2, p. 129–132, 2008.

LIASHEIK, J.; DESJARDINS, P. J.; TRIPLETT, R. G. Surgery Pain. **J Oral Maxillofac Surg**, v. 49, p. 99–103, 1987.

LIM, D.; NGEOW, W. C. A Comparative Study on the Efficacy of Submucosal Injection of Dexamethasone Versus Methylprednisolone in Reducing Postoperative Sequelae After Third Molar Surgery. **Journal of Oral and Maxillofacial Surgery**, v. 75, n. 11, p. 2278–2286, 2017.

LISBOA, A. H.; PILATTI, G. L. Pain control with dexamethasone, etoricoxib or ibuprofen associated with arginine in impacted third molar surgery. **Analgesia pós-operatória em exodontias de terceiros molares mandibulares inclusos: estudo comparativo com dexametasona, etoricoxibe e ibuprofeno associado à arginina.**, v. 61, n. 3, p. 335–340, 2013.

LUSTENBERGER, F. D.; GRÄTZ, K. W.; MUTZBAUER, T. S. Efficacy of ibuprofen versus lornoxicam after third molar surgery: A randomized, double-blind, crossover pilot study. **Oral and Maxillofacial Surgery**, v. 15, n. 1, p. 57–62, 2011.

MILLES, M.; DESJARDINS, P. J. Reduction of postoperative facial swelling by low-dose methylprednisolone: An experimental study. **Journal of Oral and Maxillofacial Surgery**, v. 51, n. 9, p. 987–991, 1993.

MITCHELL, D. A.; SEYMOUR, R. A. ibuprofen/codeine. **British Dental Journal**, v. 159, n. 12, p. 78–81, 1985.

MONY, D.; KULKARNI, D.; SHETTY, L. Comparative evaluation of preemptive analgesic effect of injected intramuscular diclofenac and ketorolac after third molar surgery-A randomized controlled trial. **Journal of Clinical and Diagnostic Research**, v. 10, n. 6, p. 102–106, 2016.

MORSE, Z.; TUMP, A.; KEVELHAM, E. Ibuprofen as a pre-emptive analgesic is as effective as rofecoxib for mandibular third molar surgery. **Odontology**, v. 94, n. 1, p. 59–63, 2006.

NEUPERT III, E. A. *et al*. of Postsurgical Sequelae of Third Molar Removal. **J Oral Maxillofac Surg**, v. 50, p. 1177–1182, 1992.

NEYCHEV, D.; CHENCHEV, I.; SIMITCHIEV, K. Analysis of Postoperative Pain After Extraction of Impacted Mandibular Third Molars and Administration of Preemptive Analgesia. **Journal of IMAB - Annual Proceeding (Scientific Papers)**, v. 23, n. 3, p. 1697–1701, 2017.

ONG, K. S.; TAN, J. M. L. WITHDRAWN: Preoperative intravenous tramadol versus ketorolac for preventing postoperative pain after third molar surgery. **International Journal of Oral and Maxillofacial Surgery**, v. 33, p. 274–278, 2004.

OROZCO-SOLÍS, M. *et al*. Single dose of diclofenac or meloxicam for control of pain, facial swelling, and trismus in oral surgery. **Medicina Oral, Patologia Oral y Cirugia Bucal**, v. 21, n. 1, p. 127–134, 2016.

PEDERSEN, A. Decadronphosphate® in the relief of complaints after third molar surgery: A double-blind, controlled trial with bilateral oral surgery. **International Journal of Oral Surgery**, v. 14, n. 3, p. 235–240, 1985.

PEKTAS, Z. O. *et al*. A comparison of pre-emptive analgesic efficacy of diflunisal and lornoxicam for postoperative pain management: a prospective, randomized, single-blind, crossover study. **International Journal of Oral and Maxillofacial Surgery**, v. 36, n. 2, p. 123–127, 2007.

PEREIRA, G. *et al*. Effect of preemptive analgesia with ibuprofen in the control of postoperative pain in dental implant surgeries: A randomized, triple-blind controlled clinical trial. **Journal of Clinical and Experimental Dentistry**, v. 12, n. 1, p. e71–e78, 2020.

SÁNCHEZ-PÉREZ, A. *et al*. Effects of the preoperative administration of dexketoprofen trometamol on pain and swelling after implant surgery: A randomized, double-blind controlled trial. **Journal of Oral Implantology**, v. 44, n. 2, p. 122–129, 2018.

SARAVANAN, K. *et al*. A Single Pre Operative Dose of Sub Mucosal Dexamethasone is Effective in Improving Post Operative Quality of Life in the Surgical Management of Impacted Third Molars: A Comparative Randomised Prospective Study. **Journal of Maxillofacial and Oral Surgery**, v. 15, n. 1, p. 67–71, 2016.

SENER, M. *et al*. Comparison of preemptive analgesic effects of a single dose of nonopioid analgesics for pain management after ambulatory surgery: A prospective, randomized, single-blind studyin Turkish patients. **Current Therapeutic Research - Clinical and Experimental**, v. 66, n. 6, p. 541–551, 2005.

SHAH, A. V. *et al*. Comparative Evaluation of Pre-Emptive Analgesic Efficacy of Intramuscular Ketorolac Versus Tramadol Following Third Molar Surgery. **Journal of Maxillofacial and Oral Surgery**, v. 12, n. 2, p. 197–202, 2013.

SIMONE, J. L. *et al*. Comparative analysis of preemptive analgesic effect of dexamethasone and diclofenac following third molar surgery. **Brazilian Oral Research**, v. 27, n. 3, p. 266–271, 2013.

SINGH, A. *et al*. International prospective register of systematic reviews Comparison between efficacy of preoperative dexamethasone and methylprednisolone in mandibular third molar surgery in reducing post-operative pain , edema and trismus – A systematic review and meta-. p. 1–4, 2020.

SOTTO-MAIOR, B. S.; SENNA, P. M.; ASSIS, N. M. D. S. P. Corticosteroids or cyclooxygenase 2-selective inhibitor medication for the management of pain and swelling after third-molar surgery. **Journal of Craniofacial Surgery**, v. 22, n. 2, p. 758–762, 2011.

STEFFENS, J. P. *et al*. Preemptive Dexamethasone and Etoricoxib for Pain and Discomfort Prevention After Periodontal Surgery: A Double-Masked, Crossover, Controlled Clinical Trial. **Journal of Periodontology**, v. 81, n. 8, p. 1153–1160, 2010.

TROMBELLI, L. *et al*. Effect of pretreatment with ketorolac tromethamine on post-operative pain following periodontal surgery. **Journal of clinical periodontology**, v. 23, n. 2, p. 128–132, 1996.

ÜSTÜN, Y. *et al*. Comparison of the effects of 2 doses of methylprednisolone on pain, swelling, and trismus after third molar surgery. **Oral Surgery, Oral Medicine, Oral Pathology, Oral Radiology, and Endodontics**, v. 96, n. 5, p. 535–539, 2003.
